# Supplementary figures and images for: Rationale design and efficacy of a smartphone application for improving self-awareness of adherence to edoxaban treatment: study protocol for a randomised controlled trial (adhere app)
Source: BMJ Open. 2022 Apr 26;12(4):e048777. doi: 10.1136/bmjopen-2021-048777 (PMC9047822; doi:10.1136/bmjopen-2021-048777)

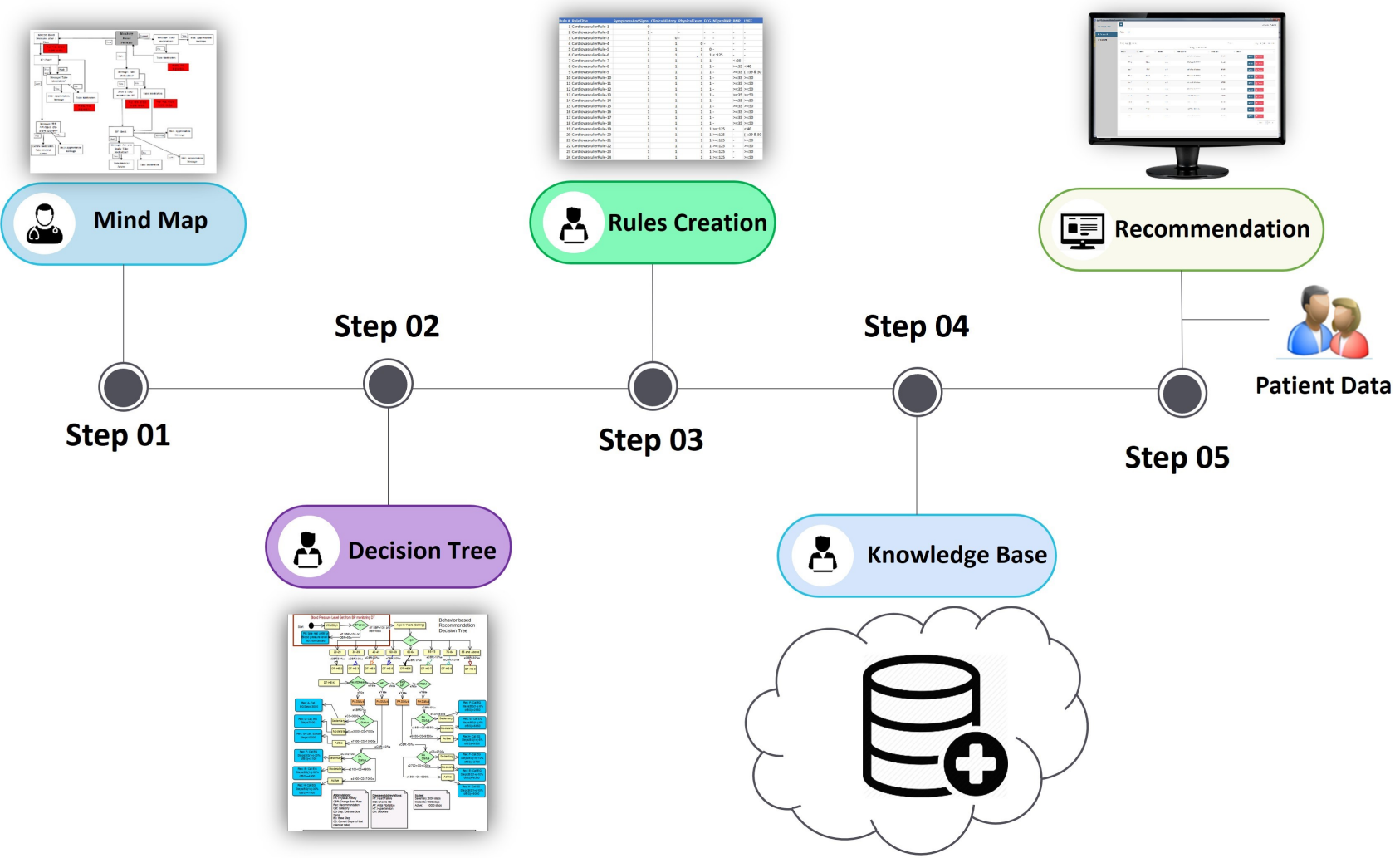

Supplement: Supplementary data [file bmjopen-2021-048777supp001.pdf]

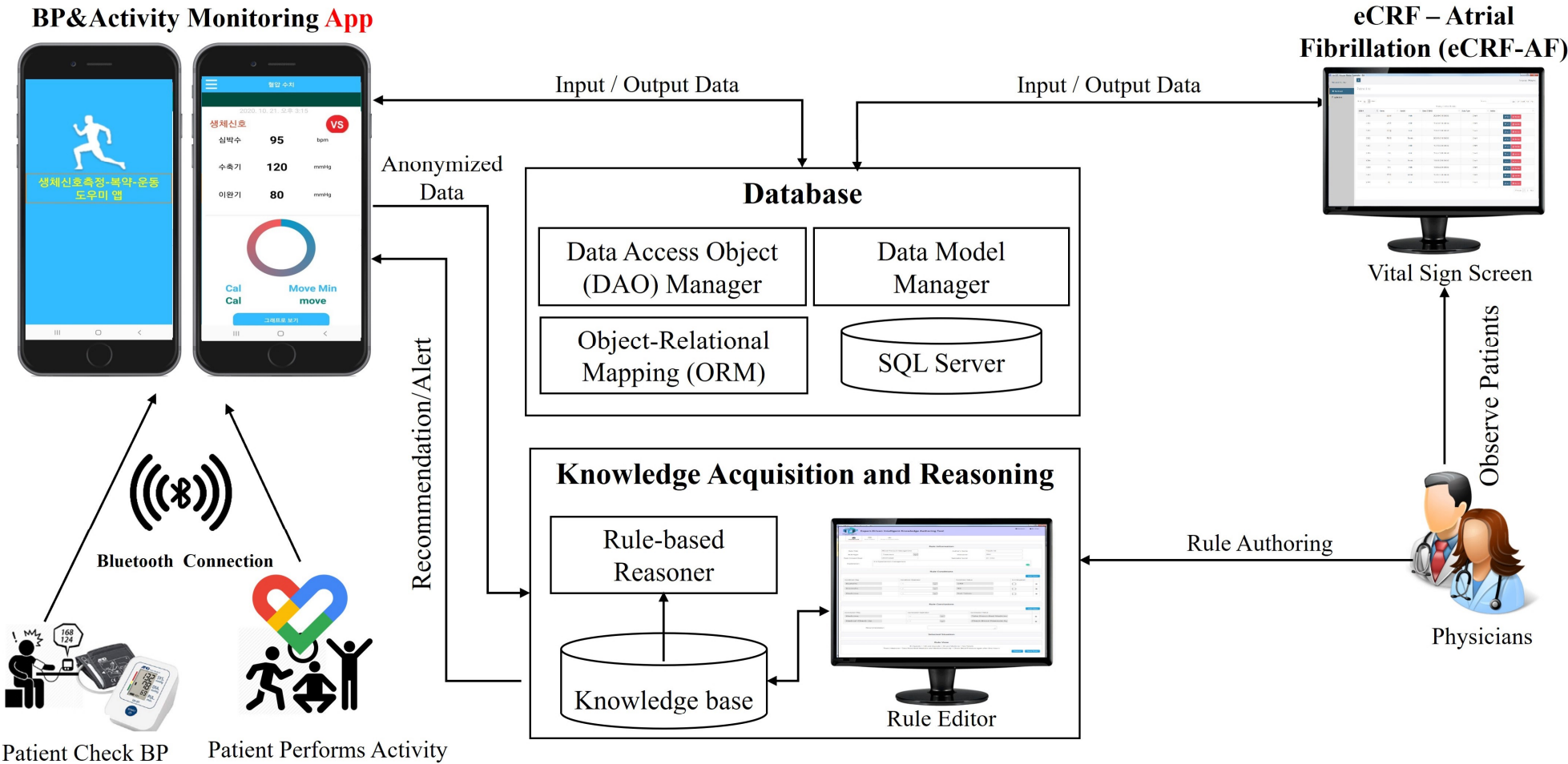

Supplement: Supplementary data [file bmjopen-2021-048777supp002.pdf]

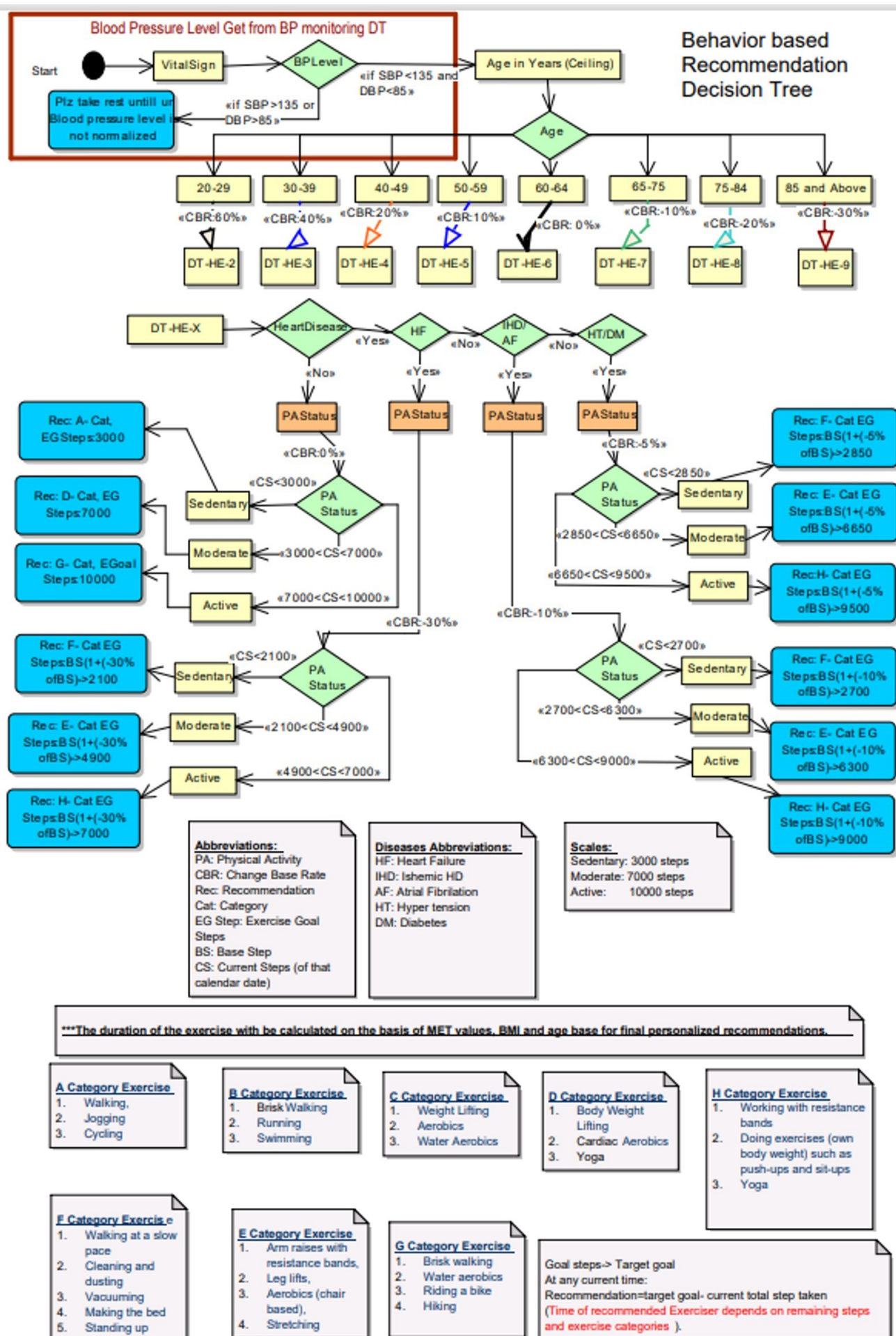

Supplement: Supplementary data [file bmjopen-2021-048777supp003.pdf]

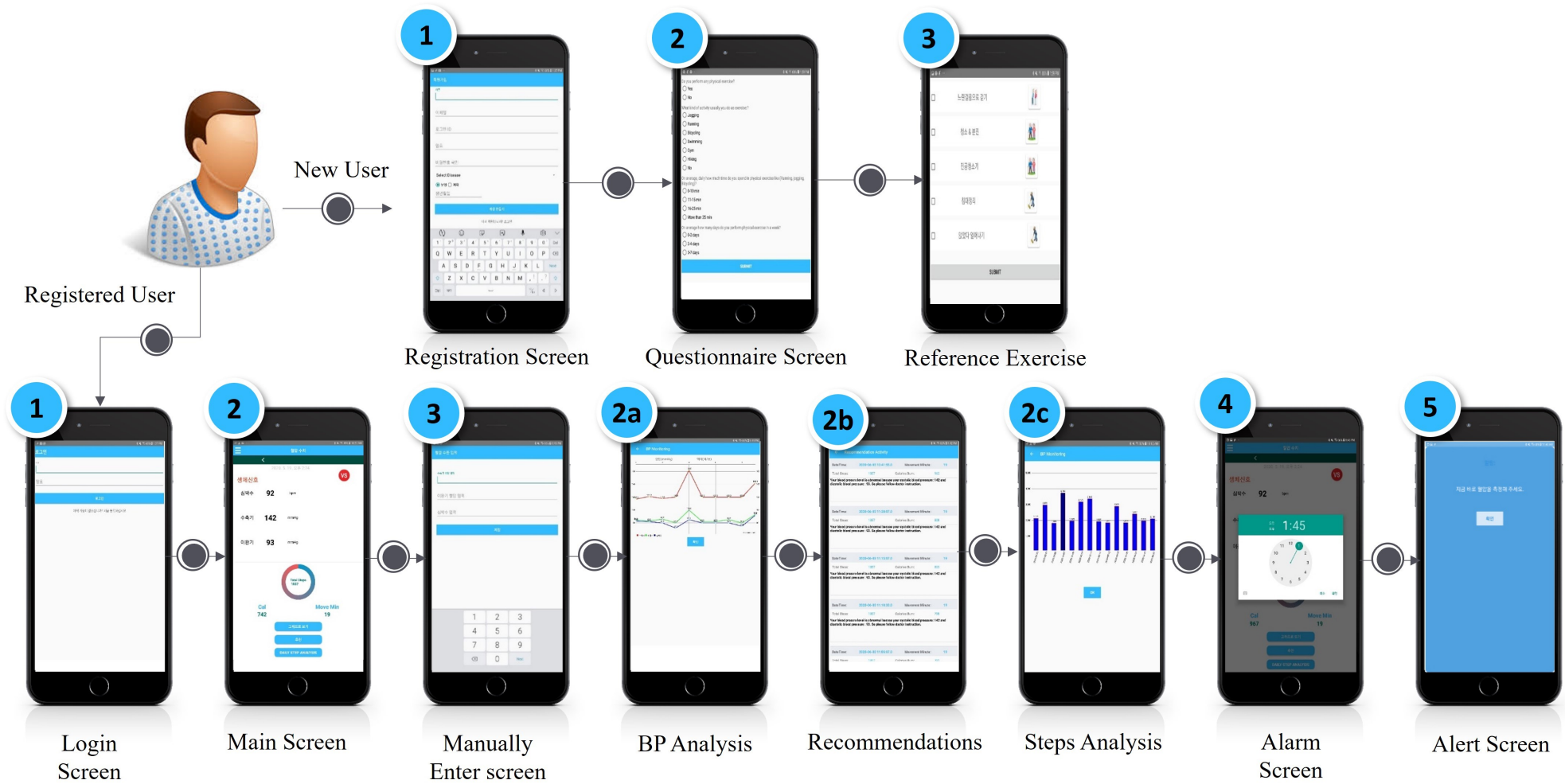

Supplement: Supplementary data [file bmjopen-2021-048777supp004.pdf]
