# Supplementary material for: Rationale design and efficacy of a smartphone application for improving self-awareness of adherence to edoxaban treatment: study protocol for a randomised controlled trial (adhere app)
Source: BMJ Open. 2022 Apr 26;12(4):e048777. doi: 10.1136/bmjopen-2021-048777 (PMC9047822; doi:10.1136/bmjopen-2021-048777)
Supplement: Supplementary data [file bmjopen-2021-048777supp005.pdf]

Supplementary Material

Rationale, Design, and Efficacy of a Smartphone Application for Improving Self-Awareness of Adherence to Edoxaban Treatment: A Randomized Controlled Study (Adhere App)

System Development Process

Previously, we described a five-step process to design and develop a standalone application (app) for patient blood pressure (BP) monitoring and physician's assistance. This app is currently undergoing clinical trial. In this study, we changed the last two steps of the previously described process. After a successful clinical trial (WHO ICRTP registry, ID: KCT0004754), we will evaluate the system. In this study, we developed an app with an additional functionality for monitoring the patient's physical activities. The primary functionalities of the system include patient BP and activity monitoring, medication adherence, and recommendations along with alert generation. The developed apps include a BP&Activity monitoring mobile app, electronic case report form (eCRF)-atrial fibrillation (AF), and Rule Editor. The primary users of the mobile app are registered patients, while the other two apps (eCRF-AF and Rule Editor) are mainly used by physicians to monitor the associated patients' activities and for knowledge creation, respectively. The aforementioned apps were designed and developed based on the five-step process. Supplemental Figure 1 shows the complete system design and development process.

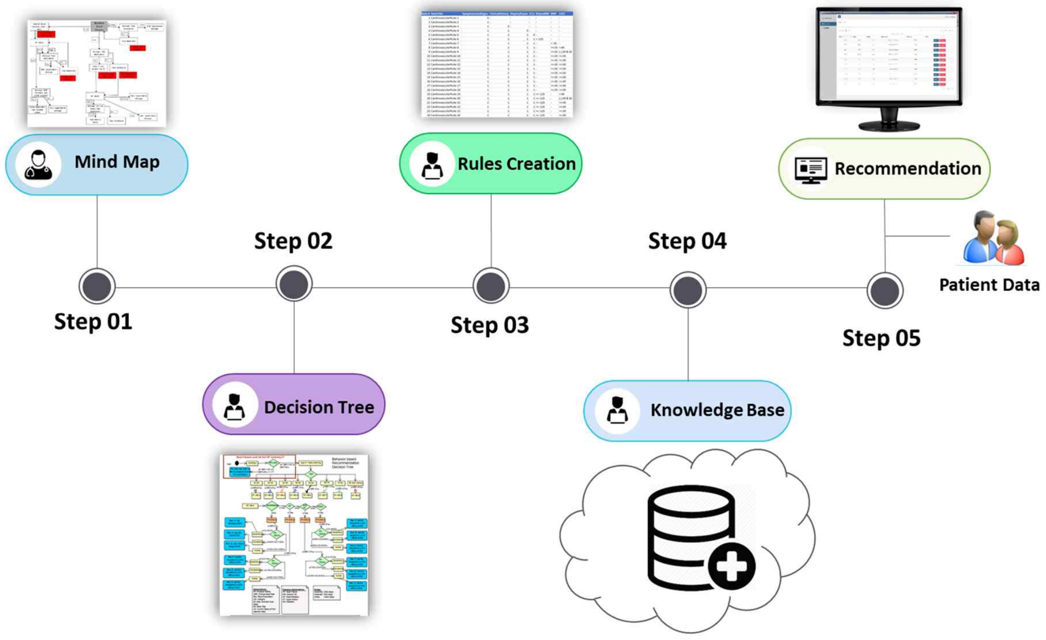

Supplemental Figure 1: System design and development process

- **Mind Map:** First, we represented the physicians' knowledge in an informal format, called the mind map, that is easily understandable to a human but not understandable to a computer. The physicians' knowledge about BP, physical activities, and their correlation was represented in the form of mind map nodes, and the nodes were related among each other based on the conditional relationships. With this analogy, the BP is linked with the age of the patient to identify the recommended activity for the patient. The resultant recommendations about the BP (systolic and diastolic), age, and current status of patient activity are connected to further diagnosis and then treatment of the patient's current condition. In short, physicians' knowledge based on their experience during real practice is represented in the mind map. We modify the initial mind map based on discussion with concerned physicians during a knowledge elicitation phase, and the final mind map is produced after a few knowledge elicitation meetings.
- **Iterative Decision Tree:** In the second phase, we transform the non-formal representation of the mind map into a more formal representation, known as the Iterative Decision Tree (IDT) [1]. The IDT representation is more understandable for physicians and computers; however,

transformation is needed for execution. In the IDT, the contributing factors are highlighted and linked with different tests to cover all the possible paths to reach diagnostic and treatment decisions. In this study, we recommend suitable physical activities to the patients based on their ages. Therefore, the patient first checks their BP, and then the system will generate a recommendation based on the recorded BP and the patient's age. Exercise goal steps (EGS) are calculated and recommended by the system to the patient to achieve the specified goal. Physicians' knowledge regarding BP is correlated with the physical activity, as shown in Supplemental Figure 2. The physicians verified and improved the IDT for all possible cases of that disease in multiple iterations of the knowledge elicitation process.

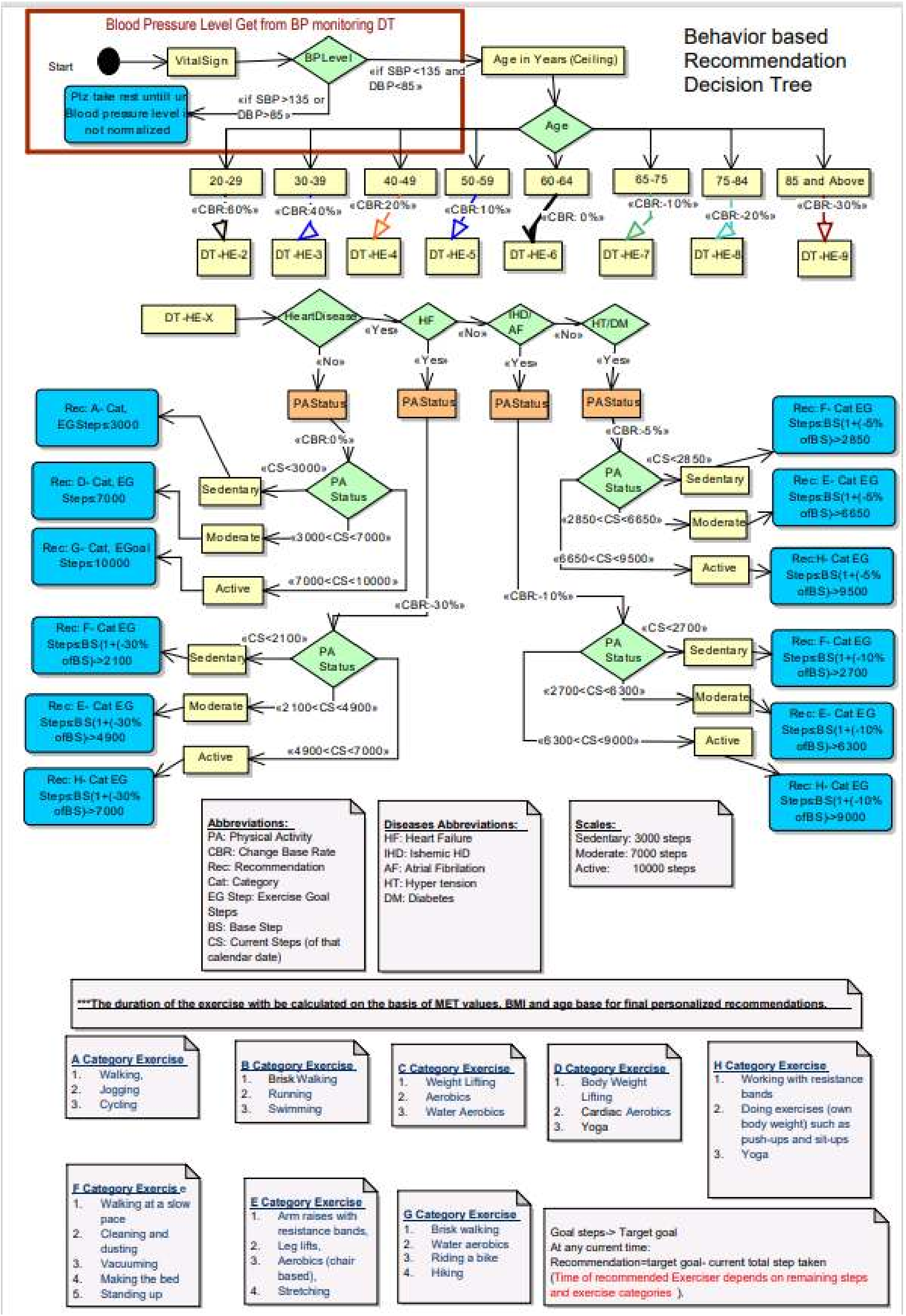

Supplemental Figure 2: Final Iterative Decision Tree (IDT) for blood pressure with physical activity

- **Rule Creation:** As previously mentioned, the IDT representation needs to be transformed into an executable format for the operative recommendations. Therefore, in the rule creation process, we transform the IDT into production rules. The production rules in object-oriented knowledge

bases are an easy way for knowledge execution. In this step, all the IDT branches are transformed into rules by top-down and left-right traversing and reasoning approaches. Our developed knowledge environment, called the Intelligent-Knowledge Authoring Tool (I-KAT), is used to transform rules into production and executable formats.

- **Knowledge Maintenance:** The knowledge maintenance phase provides a sophisticated method for validation and verification of the knowledge base. The transformed rules in the previous step are retrieved for validation and verification using a rule-based reasoning methodology. This aims to find conflict and duplication in the existing rules, if they exist. In this phase, all the rules are stored in the knowledge base in the proper hierarchy of knowledge representation.
- **Recommendation:** The recommendation phase is needed to give recommendations based on the knowledge created in the previous step. Therefore, an app is developed to provide an executable environment, which consists of a rule-based reasoner and a user-friendly interface to handle the input of signs, symptoms, history, and medical test reports to produce a final diagnosis and treatment. A mobile app was developed for BP and physical activity monitoring interventions to support patients. The app prompts the patient to monitor their BP and asks about medication intake. Based on their BP condition and medication intake status, the app recommends a suitable physical activity to perform on right time. If the condition is severe, the app recommends approaching the hospital urgently.

System Architecture and Structure

Supplemental Figure 3 shows the architecture and holistic structure of the system, which consists of four core components: (I) BP&Activity monitoring app, (II) knowledge acquisition and reasoning, (III) database, and (IV) eCRF-AF.

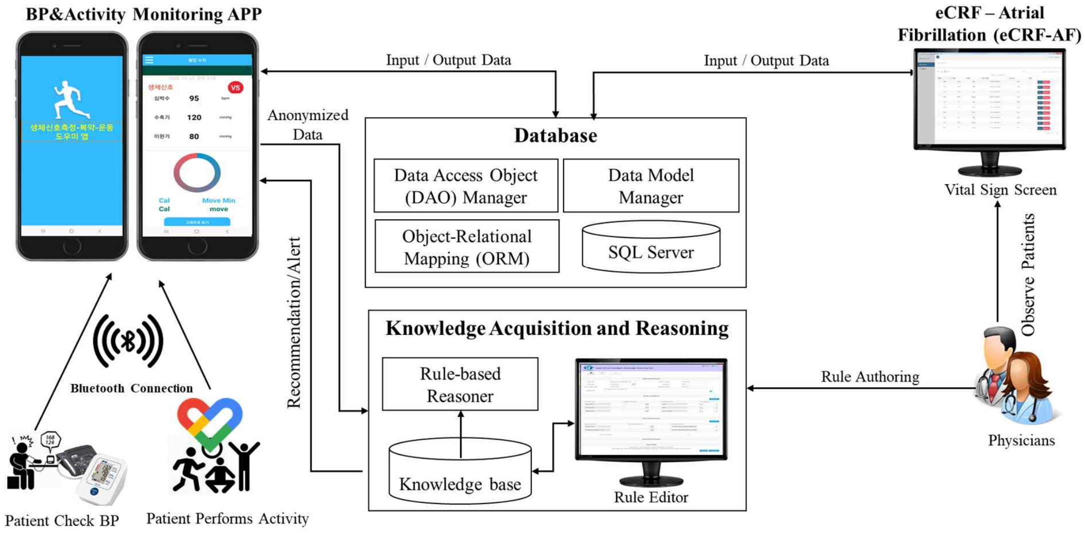

Supplemental Figure 3: System architecture and structural workflow

I. **BP&Activity Monitoring App:** This mobile app is equipped with different functionalities, such as BP and physical activity monitoring, user authentication, user registration, user exercise preferences, dashboard, BP monitoring graph, step-count graph, and recommendation screens. The core screen of the BP&Activity monitoring app is the dashboard, which facilitates users to connect with the BP monitoring device, such as the Bluetooth-enabled BP monitor UA-651BLE (A&D medical, Sydney, Australia). The dashboard screen is responsible for connecting the app with a nearby A&D PB monitoring device via Bluetooth. Similarly, the dashboard has implicit services to count the patients' steps while walking, running, jogging, and performing other physical activities. When the users check their BP, the calculated systolic BP (SBP), diastolic BP (DBP), and heart rate (HR) are sent to the mobile app. The app activates the reasoner to provide recommendations based on the calculated values. In this study, the app also considers patient age and current behavior regarding physical activity.

II. **Knowledge acquisition and reasoning:** In this component, our system provides a knowledge creation environment and reasoning capability. The I-KAT is equipped with a Rule Editor, which facilitates the creation, modification, and management of the knowledge base by physicians. The knowledge base is a core component of the clinical decision support system to

store and maintain clinical knowledge. We are presenting an authoring environment called a Rule Editor that provides an easy-to-use interface for physicians to create clinical knowledge rules. The tool reduces the burden of memorizing and understanding the data model and vocabulary concepts for the physician because the system is equipped with intelli-sense functionality during rule creation. The reasoning capability also handles knowledge validation and verification to find duplicated and conflicted rules within the knowledge base. Finally, the rules persist in the knowledge base. When it receives a patient's recorded BP, the rule-based reasoner is activated to generate recommendations and alerts based on the recorded BP.

III. **Database:** We designed and implemented the database schema to maintain patient demographic data, vital signs (systolic BP, diastolic BP, and HR), physical activity data, and other patient-related data in the database. We implemented two types of database storage: the one for the mobile app is a lightweight database using SQL Lite, which is the default temporary storage for patient data on the smartphone, when the device is not connected to the internet. When the device is connected to the internet, the data are sent to an SQL server for long-term (permanent) storage. Three internal components, data model manager, data access object (DAO) manager, and object relational mapping (ORM) work collaboratively to perform the persistence functionality. The data model manager is responsible for managing the database schema along with data instances to store in the appropriate model. The data access object (DAO) manager is used to access the data objects of the recorded values and prepare the data model to store in the database. The object relational mapping (ORM) is used to convert data between incompatible type systems using object-oriented programming languages. The App Server/Rest API is designed and developed for storing patient information, with patient consensus, for tele-monitoring by the concerned physicians. The patient data are anonymized during communication and persistence. Therefore, only the authorized and concerned physicians can see and analyze only their assigned patients' data.

IV. **eCRF–atrial fibrillation**: Generally, the eCRF system collects patient data during clinical trials. The eCRF system usually collects patient vital sign information. SBP, DBP, and HR are

important vital signs that are managed within the eCRF system. We designed and developed a special purpose eCRF for patients with AF, called eCRF-AF to monitor vital signs (SBP, DBP, and HR) along with physical activities of patients. Therefore, the BP&Activity monitoring app sends the SBP, DBP, and HR and the step counts along with physical activities (sitting, walking, running, jogging, etc.) to the vital sign and physical activity repositories, respectively. These are shown to the physicians on separate analytical screens depending on the nature of the data. The eCRF-AF systems and HMIS systems of different hospitals can be integrated with the BP&Activity monitoring app. We focused and integrated the BP&Activity monitoring app with a lightweight eCRF-AF system developed for managing clinical trials. Physicians can easily observe the BP trends (daily, weekly, monthly, yearly, etc.) along with exercise, using physical activity trends of the concerned patients. If the physicians find an abnormality in the patient data, they will communicate with the respective patient.

System Implementation Outcome: Process Flow

The system comprises three main apps: one is a mobile app (BP&Activity monitoring app), which is a patient-oriented app, while the other two are web apps (eCRF-AF and Rule Editor), which are physician-oriented apps. Here, we focus on the patient-oriented BP&Activity monitoring app, which comprises 11 different screens with multiple functionalities. The BP&Activity monitoring app and its utilization and process flow are depicted in **Supplemental Figure 4**. The BP&Activity monitoring app has two processes: one process for new user registration and a second process for registered patients to check their BPs and physical activities to obtain suitable and correct recommendations based on the recorded BP, age, profile questionnaire, and exercise preferences.

User Registration Process: To register a new user on the system, we follow a three-step process to obtain user information.

- **Step 1:** The user will add profile information, such as name, gender, date of birth, and email address, and other information to register on the system.
- **Step 2:** The questionnaire screen is used to investigate physical behavior in terms of type of

exercise, duration of exercise, and frequency of exercise. All these questions help to calculate the patients' current behavior in terms of physical activity.

- **Step 3:** The preferences screen gives privileges to the user to select the appropriate exercises from the recommended ones to set the final recommendation as a preference.

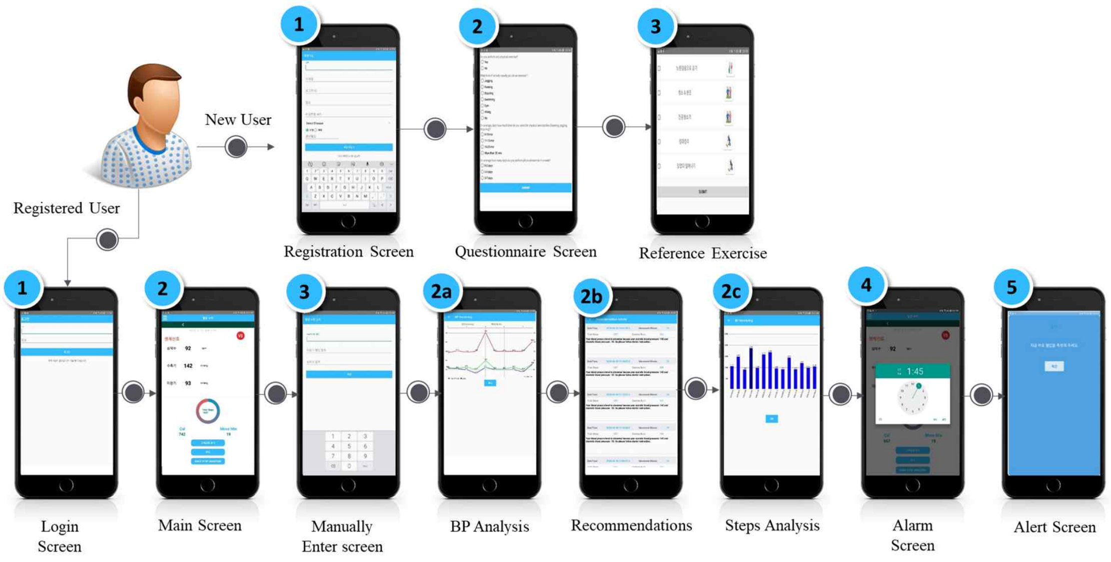

Supplemental Figure 4: Utilization and process flow of BP&Activity monitoring app (Patient-Oriented)

BP and Activity Monitoring Process: After successful registration, the patient can utilize the app to monitor their vital signs (SBP, DBP, and HR) along with their physical activity status through an A&D BP monitoring device and GoogleFit API.

- **Step 1:** The user needs to login to the system with his/her credentials for authentication.
- **Step 2:** After successful authentication, the user can access a dashboard screen. It consists of the current status of their vital signs along with the step count. It also facilitates observation of analytics and recommendation history.
- **Step 3:** The user can enter vital sign details manually when the attached device is not communicating properly. The app accepts SBP, DBP, and HR values.
- **Step 2a:** From the dashboard, the user can visualize historical analytics for vital signs, which

represent the health status in terms of BP and HR.

- **Step 2b:** From the dashboard, the user can visualize historical recommendations regarding exercises based on their physical behavior with respect to time.
- **Step 2c:** From the dashboard, the user can visualize step count historical analytics, with respect to the day, to understand their own physical behavior.
- **Step 4:** The alarm screen facilitates the user to decide and set an alarm for checking their BP. At the selected time, the system will generate the alarm for the user to check their BP and take their medication, as necessary.
- **Step 5:** The system generates recommendations at different stages of BP and activity monitoring based on patient preferences, age, and answers to the questionnaire. The recommendation may issue a message to check BP, walk, run, jog, and rest for a specified time, or to take medication at the specified time.
