# Supplementary material for: Rationale design and efficacy of a smartphone application for improving self-awareness of adherence to edoxaban treatment: study protocol for a randomised controlled trial (adhere app)
Source: BMJ Open. 2022 Apr 26;12(4):e048777. doi: 10.1136/bmjopen-2021-048777 (PMC9047822; doi:10.1136/bmjopen-2021-048777)
Supplement: Author's manuscript [file bmjopen-2021-048777.draft_revisions.pdf]

BMJ Open is committed to open peer review. As part of this commitment we make the peer review history of every article we publish publicly available.

When an article is published we post the peer reviewers' comments and the authors' responses online. We also post the versions of the paper that were used during peer review. These are the versions that the peer review comments apply to.

The versions of the paper that follow are the versions that were submitted during the peer review process. They are not the versions of record or the final published versions. They should not be cited or distributed as the published version of this manuscript.

BMJ Open is an open access journal and the full, final, typeset and author-corrected version of record of the manuscript is available on our site with no access controls, subscription charges or pay-per-view fees (http://bmjopen.bmj.com).

If you have any questions on BMJ Open's open peer review process please email info.bmjopen@bmj.com

BMJ Open

Rationale, Design, and Efficacy of a Smartphone Application for Improving Self-Awareness of Adherence to Edoxaban Treatment: A Randomized Controlled Study (Adhere App)

Journal:	*BMJ Open*
Manuscript ID	bmjopen-2021-048777
Article Type:	Protocol
Date Submitted by the Author:	06-Jan-2021

Complete List of Authors:	Kim, In-Cheol; Keimyung University Dongsan Hospital,<br>Lee, Ji Hyun ; Bundang Seoul National University Hospital Department of Internal Medicine, Cardiology<br>Choi, Dong-Ju; Seoul National University Bundang Hospital, Department of Internal Medicine<br>Park, Sung-Ji ; Samsung Medical Center, Division of Cardiology<br>Lee, Ju-Hee; Chungbuk National University College of Medicine, Department of Internal Medicine<br>Park, Sang Min; Chuncheon Sacred Heart Hospital<br>Kim, Mina; Korea University Anam Hospital<br>Kim, Hack-Lyoung; Seoul National University Seoul Metropolitan Government Boramae Medical Center, Internal Medicine<br>Lee, Sunki; Hallym University College of Medicine<br>Kim, In Jai; CHA University, Department of Internal Medicine<br>Choi, Seonghoon; Kangnam Sacred Heart Hospital<br>Bang, Jaehun; Kyung Hee University, Department of Computer Science and Engineering<br>Ali, Bilal; Kyung Hee University, Department of Computer Science and Engineering<br>Hussain, Musarrat; Kyung Hee University, Department of Computer Science and Engineering<br>Ali, Taqdir; Kyung Hee University, Department of Computer Science and Engineering<br>Lee, Sungyoung; Kyung Hee University, Department of Computer Science and Engineering

Keywords:	MEDICAL EDUCATION & TRAINING, Cardiology < INTERNAL MEDICINE, Stroke < NEUROLOGY

SCHOLARONE™ Manuscripts

Rationale, Design, and Efficacy of a Smartphone Application for Improving Self-Awareness of Adherence to Edoxaban Treatment: A Randomized Controlled Study (Adhere App)

In-Cheol Kim[1], Ji-Hyun Lee[2], Dong-Ju Choi[2], Sung-Ji Park[3], Ju-Hee Lee[4], Sang Min Park[5], Mina Kim[6], Hack-Lyoung Kim[7], Sunki Lee[8], In Jai Kim[9], Seounghoon Choi[10], Jaehun Bang[11], Bilal Ali[11], Musarrat Hussain[11], Taqdir Ali[11], and Sungyoung Lee[11]

1) Division of Cardiology, Department of Internal Medicine, Keimyung University Dongsan Hospital, Keimyung University School of Medicine, Daegu, Korea

2) Division of Cardiology and Cardiovascular Center, Department of Internal Medicine, Seoul National University Bundang Hospital, Seongnam, Korea

3) Division of Cardiology, Department of Internal Medicine, Heart Vascular Stroke Institute, Samsung Medical Center, Sungkyunkwan University School of Medicine, Seoul, Korea.

4) Division of Cardiology, Department of Internal Medicine, Chungbuk National University Hospital, Chungbuk National University College of Medicine, Cheongju, Korea

5) Department of Cardiology, Chuncheon Sacred Heart Hospital, Hallym University, Chuncheon, Korea

6) Department of Cardiology, Korea University College of Medicine, Seoul, Korea

7) Division of Cardiology, Seoul National University Boramae Medical Center, Seoul, Korea

8) Division of Cardiology, Dongtan Sacred Heart Hospital, Hallym University College of Medicine, Gyunggi-do, Korea

For peer review only

9) Division of Cardiology, Department of Internal Medicine, Bundang CHA Medical Center, CHA University, Seongnam, Korea

10) Division of Cardiology, Department of Internal Medicine, Kangnam Sacred Heart Hospital, Hallym University College of Medicine, Seoul, Korea

11) Department of Computer Science and Engineering, Kyung Hee University, Yongin, Korea

* IC Kim and JH Lee contributed equally to this work

Word counts

Abstract: 289 words

Main text (excluding title page, abstract, references, figures, and tables): 2,310 words

Correspondence

Dong-Ju Choi, M.D. Ph.D. (https://orcid.org/0000-0003-0146-2189)

Division of Cardiology and Cardiovascular Center, Department of Internal Medicine, Seoul National University Bundang Hospital, Seongnam, Korea.

82, gumi-ro 173 Beon-gil, Bundang-gu, Seongnam, Gyeonggido, 13620, Korea

Tel: 82-31-787-7007, Fax: 82-31-787-4051

E-mail: djchoi@snubh.org

For peer review only

For peer review only

ABSTRACT

Introduction: High adherence to oral anticoagulants is essential for stroke prevention in patients with atrial fibrillation (AF). We developed a smartphone application (app) that pushes alarms for taking medication and measuring blood pressure (BP) and heart rate (HR) at certain times of the day. In addition to drug alarms, the habit of measuring one's BP and HR may reinforce drug adherence by improving self-awareness of the disease. This pilot study aims to test the feasibility and efficacy of the smartphone app-based intervention for improving drug adherence in patients with AF.

Methods and analysis: A total of 10 university hospitals in Korea will participate in this randomized control trial. Patients with AF, being treated with edoxaban for stroke prevention will be included in this study. The patients will be randomized to the conventional treatment group and the app conditional feedback group. Patients in the app conditional feedback group will use the medication reminder app for medication and BP check alarms. The automatic BP machine will be linked to the smartphone via Bluetooth. The measured BP and HR will be updated automatically on the smartphone app. The primary endpoint is edoxaban adherence by pill count measurement at three and six months of follow-up. Secondary endpoints are clinical composite endpoints including stroke, systemic embolic event, major bleeding requiring hospitalization or transfusion, or death during the six-months. As of 6 November 2020, patient enrollment had not started.

Ethics and dissemination: This study was approved by the Seoul National University Bundang Hospital Institutional Review Board and will be conducted according to the principles of the Declaration of Helsinki. The study results will be published in a reputable journal.

Trial registration: WHO ICRTP registry, ID: KCT0004754. As of 6 November 2020, recruitment had not started.

Key words

atrial fibrillation; drug adherence; smartphone application; edoxaban

For peer review only

Strengths and limitations of this study.

- This randomized control trial investigates the feasibility and efficacy of a smartphone app-based intervention for improving adherence to edoxaban in multiple centers.
- This trial will unveil whether self-care behavior can improve disease awareness which might further reinforce drug adherence.
- The sample size was calculated to show the difference in drug adherence, and not for any clinical events.
- The study results may not be applicable to patients who are not capable of using smartphones.

For peer review only

INTRODUCTION

Stroke prevention with oral anticoagulants is crucial in the management of atrial fibrillation (AF).[1] the Large randomized control trials with AF patients have consistently shown comparable effectiveness and improved safety of non-vitamin K oral anti-coagulants (NOAC) compared with warfarin.[2-6] Based on these trials, the current global guidelines recommend NOACs over warfarin for stroke prevention in patients with AF.[1 7 8]

In contrast to warfarin, NOACs have predictable effectiveness and minimal drug interactions. Consequently, regular blood tests for the monitoring of anticoagulation effects are not necessary with NOAC therapy. The use of NOACs is increasing worldwide because of these advantages.[9 10] However, there are some concerns regarding the relatively short half-life on NOACs. When doses are missed, a prothrombotic status might adversely triggered.[11] Thus, in NOAC therapy, consistent drug adherence is essential to maintain a consistent anticoagulation effect for stroke prevention. Poor drug adherence or failed persistence would increase the risk of stroke, and this may lead to an increase in public health care costs.[12]

Even though there are controversies regarding their efficacy, smartphone applications (apps) are currently being recognized as an effective tool for promoting drug adherence[13-23]. Smartphone apps send notifications or push alarms reminding the patient to take their medication. Moreover, active involvement in measuring their health status – blood pressure (BP) and heart rate (HR) using smartphone app-based feedback system could improve awareness of their underlying condition. This in turn influences their self-care behavior and increases drug adherence[24]. The aim of this study protocol is to investigate the feasibility and efficacy of a smartphone app-based intervention that provides push alarms for taking medication and measuring BP and HR in patients with AF requiring oral anticoagulation therapy.

For peer review only

METHODS AND ANALYSIS

Study design

The Adhere-App (Self-awareness of Drug Adherence to Edoxaban Using an Automatic App. Feedback System) study is a multicenter, randomized, open-label, and group-comparison trial to assess the effect of using a smartphone app to improve drug adherence (**Figure 1**). A total of 10 tertiary university hospitals in Korea will participate in this study. The study participants will be randomized to a smartphone app-conditioned feedback group or a conventional treatment group. Every patient will receive standardized education on AF and the importance of anticoagulation therapy. Additionally, the intervention group will be educated on the use of the smartphone app coupled with BP and HR measurement using an automatic BP measuring machine. Study visits will be performed at baseline, three months, and six months. When clinical visits are not possible, the patient's condition and survey data can be collected by telephone visit. This study was registered in the International Clinical Trials Registry Platform (ICTRP) on 20 February 2020. However, initiation was delayed due to coronavirus disease 2019 (COVID-19) pandemic. As of 6 November 2020, patient enrollment is expected to be initiated in a few weeks and is expected to be completed in late 2022.

Study participants

Adult patients (≥ 19 years old) with non-valvular AF requiring oral anticoagulation therapy (CHA_2DS_2-VASc score ≥ 2 points) for stroke prevention will be enrolled. Edoxaban (Lixiana®, Daiichi Sankyo, Tokyo, Japan) will be administered to patients at an on-label dosage of 60 mg once daily. The dosage will be reduced to 30 mg once daily in patients who meet any of the following criteria: moderate renal impairment (creatinine clearance 30-50 mL/min), body weight less than or equal to 60 kg, or concomitant use of potent P-glycoprotein inhibitors.

For peer review only

Since smartphone usage is crucial in this study, participants should be able to use smartphone well, and be capable of following instructions on how to use the app. Patients with severe renal insufficiency (creatinine clearance < 15 mL/min) and significant mitral valve disease will be excluded. The detailed inclusion and exclusion criteria for the Adhere-App study are listed in **Table 1**.

Table 1. Key inclusion and exclusion criteria for Adhere-App study.

Inclusion criteria<br>• Patients with atrial fibrillation, treated with edoxaban for stroke prevention<br>• Age ≥ 19 years<br>• Capable of using smartphone application<br>• Accepted for the study protocol and willing to participate in the clinical study
Exclusion criteria<br>• Creatinine clearance less than 15 mL/min<br>• Patients with dual antiplatelet therapy<br>• Moderate or severe mitral stenosis<br>• Previous history of mitral valve replacement or mitral valve repair<br>• Previous history of alcohol or drug abuse<br>• Not suitable for the clinical trial enrollment by the judgement of the investigator<br>• History of previous enrollment in another clinical trial using an investigational pharmaceutical product

For peer review only

- Patient unwilling to participate in the clinical study

At baseline, age, sex, details of comorbidities, and laboratory findings will be assessed for all patients. The CHA_2DS_2-VASc score for stroke risk prediction is calculated by the summation of all assigned points for each particular medical condition: one point each for age between 65 and 74 years (A), female sex (Sc), hypertension (H), diabetes mellitus (D), congestive heart failure (C), and vascular disease (V, prior myocardial infarction or peripheral artery disease) and 2 points each for a history of stroke/transient ischemic attack/thromboembolism (S_2) or age of $\geq$ 75 years (A_2).[25]

Patient and public involvement

Patients or the public will not be involved in the design, reporting, or dissemination of our research.

Patient recruitment and randomization

Potential participants will be any patients visiting the clinic for AF management. After a comprehensive interview, written informed consent will be obtained from all eligible participants. Eligible participants will be asked to complete a baseline survey covering demographics and cardiovascular comorbidities. The eligible participants will then be randomly assigned to either the intervention group or the conventional treatment group in a 1:1 ratio (**Figure 2**). Treatment assignments will be performed centrally according to a computer-generated random schedule in permuted blocks within age strata (<60 years and ≥60 years). Due to the nature of the intervention, it is not possible to blind participants to group allocation. All participants will be informed that the information on edoxaban adherence will be collected for analysis.

For peer review only

Intervention (Smartphone app-conditioned feedback)

Study participants in the intervention group will download the app from the Google Play Store. The operating system and sample display of the smartphone app are summarized in **Figure 3**. The participants can set the alarm for taking edoxaban at a certain time of the day. The research coordinator or the physician will set the alarm initially. Patient's exercise information, including interval, exercise type, and duration, will be submitted on the first visit. When the alarm sounds at the pre-specified time, the patient will be asked to take the drug (edoxaban) and measure their BP and HR using an automated electronic manometer (UA-651BLE Bluetooth Blood Pressure monitor, A&D Medical, Sydney, Australia). The measured BP and HR data will be automatically transmitted to the smartphone via Bluetooth, as shown in **Supplemental Figure 2**. To evaluate awareness of disease status, the app will ask whether the measured BP result is optimal, and the patient is required to respond to the question. The app will also check whether the patient took the medication. If the patient does not affirm taking the medication, the app will send an alert message to ensure that the patient takes the medication. The data regarding the time of edoxaban intake, BP, and HR will be automatically updated and stored daily. Finally, the physician can obtain feedback on BP and HR measurement data and drug adherence by checking the app on the follow-up visit. The aforementioned functionalities will be provided by the smartphone app, while a standalone web application has been developed for physicians to monitor associated patients' BP, physical activity, and medication adherence trends. The design, development, and utilization detail of the patient-oriented smartphone app and physician-oriented web app are described in the **Supplemental Materials**.

Control (Conventional treatment)

In the control group, only standard guideline-recommended treatments, including education on the disease and the importance of taking medications will be covered. No other specific interventions will be made. Patients will be followed up in clinics according to the study protocol (three months and six months) and be provided standard care for AF.

For peer review only

Study endpoints and follow up

The primary endpoint is edoxaban adherence by pill count measurement at second (three months) and third (six months) visits. All patients will be asked to bring their remaining pills at each clinical visit. Secondary endpoints are clinical composite endpoints, including stroke, systemic embolic event, major bleeding requiring hospitalization or transfusion, or death during the six months of follow up. An additional pre-defined observational parameter is symptom change related to BP and HR.

Sample size calculation

This is a pilot study to assess the efficacy of a smartphone-based intervention. To date, there has been no study evaluating the use of a smartphone app to improve NOAC adherence in Korea. Thus, a precise sample size calculation was not available. We assumed that drug adherences would be 90% in the control group and 95% in the intervention group. A sample size of 234 patients in each group achieves 95% power to detect a mean difference of 5.0 with a known standard deviation of differences of 15.0 and a significance level (alpha) of 0.05, using a two-sided paired z-test. Considering dropout rate of 4%, we finally planned to enroll 500 patients (250 patients in each group).

Statistical analysis

Categorical variables will be presented as numbers and frequencies, whereas continuous variables will be presented as means ± standard deviations. The student's t-test and Kruskal-Wallis test will be used to compare continuous variables depending on the presence of normally distributed variables. The chi-square test will be used to compare categorical variables. The standard regimen of edoxaban is once daily. The primary endpoint of edoxaban adherence will be calculated by pill count as shown below.

$$\text{Pill count adherence (\%)} = \frac{\text{Number of pills dispensed} - \text{Number of pills remaining}}{\text{Number of days between dispensing date and follow up date}} \times 100$$

For peer review only

Since new drug users might have a lower adherence than continuous users, subgroup analysis will also be performed on the new and continuous users. The secondary endpoints of adverse events will be compared with the Kaplan-Meier curve analysis. A log rank test will be used to evaluate the significance of the difference between the two groups. Statistical analysis will be performed using IBM SPSS Statistics for Windows (IBM Corp., Armonk, N.Y., USA) and R software version 4.0.3.

Ethics and dissemination.

This study was approved by the institutional review board of Seoul National University Bundang Hospital and will be conducted according to the principles of the Declaration of Helsinki. The potential risks to participants are negligible in the present study. The participants will be handed study information sheets and asked to provide written informed consent. The study results will be disseminated via publication in a reputable journal and presentation at scientific meetings. Individual participant data that underlie the results reported (text, tables, figures and appendices) will be shared after deidentification process upon reasonable request.

DISCUSSION

This randomized control trial investigates the feasibility and efficacy of using a smartphone app to improve adherence to anticoagulation therapy (edoxaban). Furthermore, this study will also assess the influence of app-based interventions influences on the reduction of AF-related adverse clinical events. Today, smartphones have became an integral part of everyday life. We believe that our study will be helpful when formulating public health strategies for improving NOAC adherence using smartphones.

All participants in the intervention group will be asked to input the measured BP and HR data on a smartphone, in addition to responding to a push alarm for taking their medication. The recognition of the irregular rhythm of AF during heart rate measurement could remind the participants of the necessity of anticoagulation therapy, which they will be informed about by their physician during the education

For peer review only

session. Feedback questions to remind the patient of their BP control status might further reinforce disease awareness and drug adherence. The stored information could also be used as a reference to set an optimal therapeutic goal for education and medication control.

A similar study was performed with NOACs, using a telemonitoring system accompanied by personal telephone feedback.[12] In that study, although the telemonitoring system achieved high drug adherence (over 90%), it was not cost-effective. The human resource and system set-up costs to operate telemonitoring-based feedback impeded the wide usage of this method in clinical practice. Digital interventions using smartphone apps are becoming an increasingly common way to support medication adherence and self-management in chronic disorders.[26] Smartphones also help to improve access to cardiac rehabilitation and heart failure management for patients unable to attend traditional center-based cardiac rehabilitation[27] We believe that our strategy of using a smartphone app would be an effective way to increase drug adherence at a minimum cost.

As with other current management strategies, apps can evolve into tailored programs. Besides the simple reminders, this strategy can be incorporated into a multimodal strategy that results in sustained improvements in adherence.[28] Reminders primarily focus on unintentional nonadherence, identifying the reasons for nonadherence and developing a scale that assesses unintentional nonadherence.[29-31] This randomized trial will not only evaluate the effectiveness of the reminder app on disease awareness and drug adherence in patients with AF but will also try to find components that may hinder patient adherence to NOACs. This information could be used to develop a strategy to ensure sustained improvement in adherence to current medical therapy.

Strengths and limitations

Several limitations of this study should be addressed. The study participants do not represent the general population with AF. All participants should be able to use smartphone apps without difficulties. Thus, the results of the study may not be applicable to patients who are not capable of using smartphones,

For peer review only

such as the extreme elderly. Second, the study participants who agree to participate in the study might have a higher interest in their own health. Consequently, higher adherence to drug therapy might be observed than that of the general AF population. Third, the sample size was calculated to show the difference in drug adherence. It is not powered to show any differences in clinical events. Despite these limitations, a major strength is that the present study is a multicenter randomized control trial with a relatively large number of participants.

Author Contributions

DJC, ICK, and JHL contributed to the conception of the study. DJC, ICK, JHL, and MK participated in the design of the study. ICK and JHL drafted and finalized the manuscript. SJP, JHL, SMP, MK, HLK, SL, IJK, SC, and DJC revised the manuscript critically for important intellectual content.

Funding statement

This work was supported by a grant from Daiichi Sankyo, Korea (06-2019-233). The funders have no influence on the study design, data collection or the decision to submit reports for publication.

Competing interests statement.

None declared.

For peer review only

References

1. Hindricks G, Potpara T, Dagres N, et al. 2020 ESC Guidelines for the diagnosis and management of atrial fibrillation developed in collaboration with the European Association of Cardio-Thoracic Surgery (EACTS). *Eur Heart J* 2020 doi: 10.1093/eurheartj/ehaa612

2. Ruff CT, Giugliano RP, Braunwald E, et al. Comparison of the efficacy and safety of new oral anticoagulants with warfarin in patients with atrial fibrillation: a meta-analysis of randomised trials. *Lancet* 2014;383(9921):955-62. doi: 10.1016/S0140-6736(13)62343-0

3. Connolly SJ, Ezekowitz MD, Yusuf S, et al. Dabigatran versus warfarin in patients with atrial fibrillation. *N Engl J Med* 2009;361(12):1139-51. doi: 10.1056/NEJMoa0905561

4. Patel MR, Mahaffey KW, Garg J, et al. Rivaroxaban versus warfarin in nonvalvular atrial fibrillation. *N Engl J Med* 2011;365(10):883-91. doi: 10.1056/NEJMoa1009638

5. Granger CB, Alexander JH, McMurray JJ, et al. Apixaban versus warfarin in patients with atrial fibrillation. *N Engl J Med* 2011;365(11):981-92. doi: 10.1056/NEJMoa1107039

6. Giugliano RP, Ruff CT, Braunwald E, et al. Edoxaban versus warfarin in patients with atrial fibrillation. *N Engl J Med* 2013;369(22):2093-104. doi: 10.1056/NEJMoa1310907

7. January CT, Wann LS, Calkins H, et al. 2019 AHA/ACC/HRS Focused Update of the 2014 AHA/ACC/HRS Guideline for the Management of Patients With Atrial Fibrillation: A Report of the American College of Cardiology/American Heart Association Task Force on Clinical Practice Guidelines and the Heart Rhythm Society in Collaboration With the Society of Thoracic Surgeons. *Circulation* 2019;140(2):e125-e51. doi: 10.1161/CIR.0000000000000665

8. Kirchhof P, Benussi S, Kotecha D, et al. 2016 ESC Guidelines for the management of atrial fibrillation developed in collaboration with EACTS. *Eur Heart J* 2016;37(38):2893-962. doi: 10.1093/eurheartj/ehw210

9. Lee SR, Choi EK, Han KD, et al. Temporal trends of antithrombotic therapy for stroke prevention in Korean patients with non-valvular atrial fibrillation in the era of non-vitamin K antagonist oral anticoagulants: A nationwide population-based study. *PLoS One* 2017;12(12):e0189495. doi: 10.1371/journal.pone.0189495 [published Online First: 2017/12/21]

For peer review only

10. Huisman MV, Rothman KJ, Paquette M, et al. The Changing Landscape for Stroke Prevention in AF: Findings From the GLORIA-AF Registry Phase 2. *J Am Coll Cardiol* 2017;69(7):777-85. doi: 10.1016/j.jacc.2016.11.061

11. How CH. Novel oral anticoagulants for atrial fibrillation. *Singapore Med J* 2015;56(12):657-8; quiz 59. doi: 10.11622/smedj.2015184

12. Desteghe L, Vijgen J, Koopman P, et al. Telemonitoring-based feedback improves adherence to non-vitamin K antagonist oral anticoagulants intake in patients with atrial fibrillation. *Eur Heart J* 2018;39(16):1394-403. doi: 10.1093/eurheartj/ehx762

13. Weisman O, Schonherz Y, Harel T, et al. Testing the Efficacy of a Smartphone Application in Improving Medication Adherence, Among Children with ADHD. *Isr J Psychiatry Relat Sci* 2018;55(2):59-63.

14. Morawski K, Ghazinouri R, Krumme A, et al. Association of a Smartphone Application With Medication Adherence and Blood Pressure Control: The MedISAFE-BP Randomized Clinical Trial. *JAMA Intern Med* 2018;178(6):802-09. doi: 10.1001/jamainternmed.2018.0447

15. Huang Z, Tan E, Lum E, et al. A Smartphone App to Improve Medication Adherence in Patients With Type 2 Diabetes in Asia: Feasibility Randomized Controlled Trial. *JMIR Mhealth Uhealth* 2019;7(9):e14914. doi: 10.2196/14914

16. Sarzynski E, Decker B, Thul A, et al. Beta Testing a Novel Smartphone Application to Improve Medication Adherence. *Telemed J E Health* 2017;23(4):339-48. doi: 10.1089/tmj.2016.0100

17. Yoon CH, Ritchie SR, Duffy EJ, et al. Impact of a smartphone app on prescriber adherence to antibiotic guidelines in adult patients with community acquired pneumonia or urinary tract infections. *PLoS One* 2019;14(1):e0211157. doi: 10.1371/journal.pone.0211157

18. Wu YP, Linder LA, Kanokvimankul P, et al. Use of a Smartphone Application for Prompting Oral Medication Adherence Among Adolescents and Young Adults With Cancer. *Oncol Nurs Forum* 2018;45(1):69-76. doi: 10.1188/18.ONF.69-76

19. Yu C, Liu C, Du J, et al. Smartphone-based application to improve medication adherence in patients after surgical coronary revascularization. *Am Heart J* 2020;228:17-26. doi:

10.1016/j.ahj.2020.06.019

20. Himelhoch S, Kreyenbuhl J, Palmer-Bacon J, et al. Pilot feasibility study of Heart2HAART: a smartphone application to assist with adherence among substance users living with HIV. *AIDS Care* 2017;29(7):898-904. doi: 10.1080/09540121.2016.1259454

21. Talmor G, Nguyen B, Keibel A, et al. Use of software applications to improve medication adherence and achieve more integrated disease management in heart failure. *Trends Cardiovasc Med* 2018;28(7):483-88. doi: 10.1016/j.tcm.2018.04.001

22. Johnston N, Bodegard J, Jerstrom S, et al. Effects of interactive patient smartphone support app on drug adherence and lifestyle changes in myocardial infarction patients: A randomized study. *Am Heart J* 2016;178:85-94. doi: 10.1016/j.ahj.2016.05.005

23. Marquez Contreras E, Marquez Rivero S, Rodriguez Garcia E, et al. Specific hypertension smartphone application to improve medication adherence in hypertension: a cluster-randomized trial. *Curr Med Res Opin* 2019;35(1):167-73. doi: 10.1080/03007995.2018.1549026

24. Kelders SM, Kok RN, Ossebaard HC, et al. Persuasive system design does matter: a systematic review of adherence to web-based interventions. *J Med Internet Res* 2012;14(6):e152. doi: 10.2196/jmir.2104 [published Online First: 2012/11/16]

25. Lip GY, Nieuwlaat R, Pisters R, et al. Refining clinical risk stratification for predicting stroke and thromboembolism in atrial fibrillation using a novel risk factor-based approach: the euro heart survey on atrial fibrillation. *Chest* 2010;137(2):263-72. doi: 10.1378/chest.09-1584

26. Morrissey EC, Casey M, Glynn LG, et al. Smartphone apps for improving medication adherence in hypertension: patients' perspectives. *Patient Prefer Adherence* 2018;12:813-22. doi: 10.2147/PPA.S145647 [published Online First: 2018/05/23]

27. Hamilton SJ, Mills B, Birch EM, et al. Smartphones in the secondary prevention of cardiovascular disease: a systematic review. *BMC Cardiovasc Disord* 2018;18(1):25. doi: 10.1186/s12872-018-0764-x [published Online First: 2018/02/09]

28. Haynes RB, Ackloo E, Sahota N, et al. Interventions for enhancing medication adherence. *Cochrane*

For peer review only

Database Syst Rev 2008(2):CD000011. doi: 10.1002/14651858.CD000011.pub3

29. Gadkari AS, McHorney CA. Unintentional non-adherence to chronic prescription medications: how unintentional is it really? *BMC Health Serv Res* 2012;12:98. doi: 10.1186/1472-6963-12-98

30. Dayer L, Heldenbrand S, Anderson P, et al. Smartphone medication adherence apps: potential benefits to patients and providers: response to Aungst. *J Am Pharm Assoc (2003)* 2013;53(4):345. doi: 10.1331/JAPhA.2013.13121

31. Dayer L, Heldenbrand S, Anderson P, et al. Smartphone medication adherence apps: potential benefits to patients and providers. *J Am Pharm Assoc (2003)* 2013;53(2):172-81. doi: 10.1331/JAPhA.2013.12202

For peer review only

Figure legends

Fig. 1 Schematic diagram of expected improvement in NOAC adherence by regular measurement of BP, heart rate and smartphone feedback in patients with atrial fibrillation. NOAC, non-vitamin K oral anti-coagulant; BP, blood pressure; HR, heart rate.

Fig. 2 Study design of AdhereApp study. Patients using edoxaban will be randomized to either an application conditioned-feedback group or a conventional therapy group.

Fig. 3 Operating system and sample display of the smartphone app. When the patient measures BP, the data are automatically transferred to the app via Bluetooth system. A, title page; B, menu bar indicating BP measurement, log-out, alarm setting, manual BP input, and password setting; C, alarm setting page for medication; D, exercise information including interval, exercise type, and duration; E, alarm page to check whether the patient has taken medication; F, BP manual input page; G, page displaying measured BP and HR result; H, feed-back question to check whether the patient has achieved optimal target BP. BP, blood pressure; HR, heart rate.

For peer review only

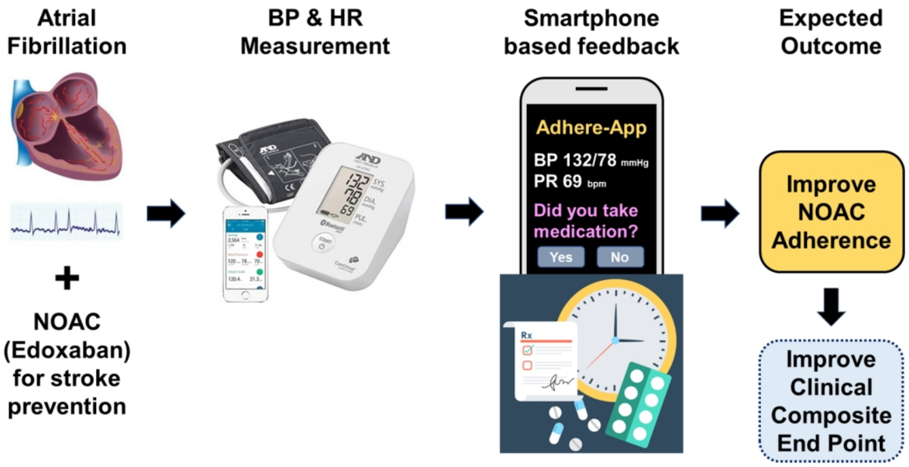

Figure1. Schematic diagram of expected improvement in NOAC adherence by regular measurement of BP, heart rate and smartphone feedback in patients with atrial fibrillation. NOAC, non-vitamin K oral anti-coagulant; BP, blood pressure; HR, heart rate.

308x163mm (150 x 150 DPI)

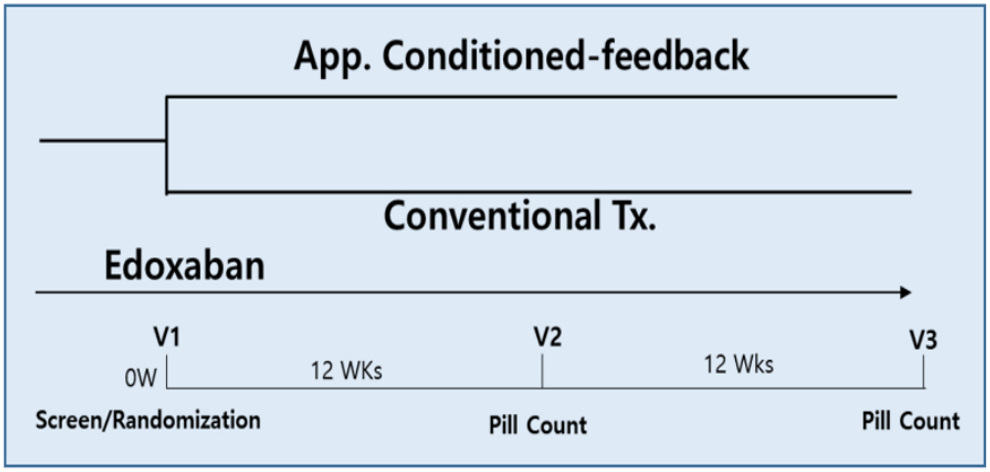

Figure 2. Study design of AdhereApp study. Patients using edoxaban will be randomized to either an application conditioned-feedback group or a conventional therapy group.

233x114mm (96 x 96 DPI)

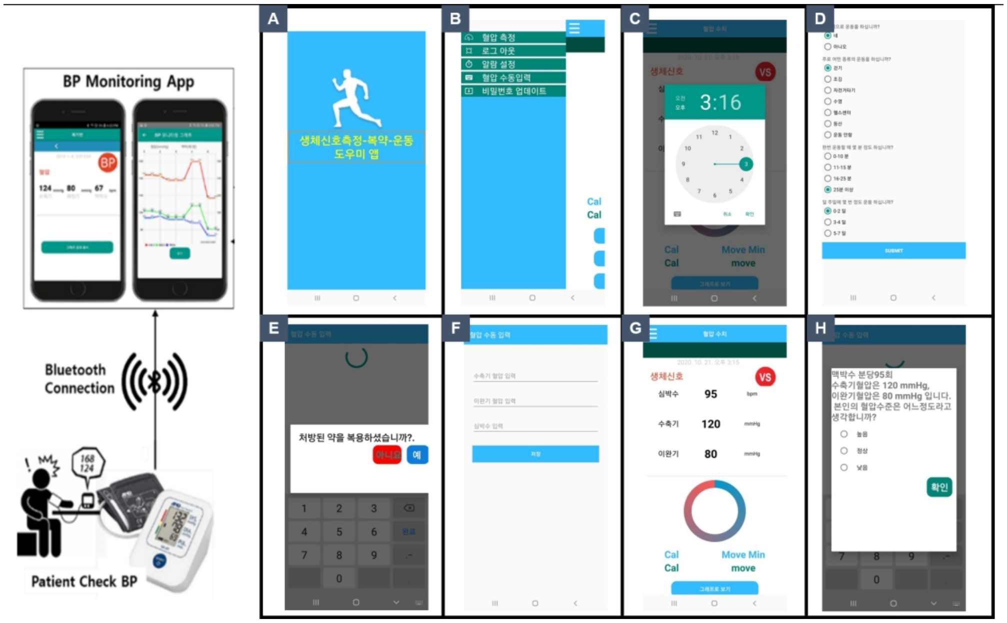

Figure 3. Operating system and sample display of the smartphone app. When the patient measures BP, the data are automatically transferred to the app via Bluetooth system. A, title page; B, menu bar indicating BP measurement, log-out, alarm setting, manual BP input, and password setting; C, alarm setting page for medication; D, exercise information including interval, exercise type, and duration; E, alarm page to check whether the patient has taken medication; F, BP manual input page; G, page displaying measured BP and HR result; H, feed-back question to check whether the patient has achieved optimal target BP. BP, blood pressure; HR, heart rate.

310x190mm (150 x 150 DPI)

Supplementary Material

Rationale, Design, and Efficacy of a Smartphone Application for Improving Self-Awareness of Adherence to Edoxaban Treatment: A Randomized Controlled Study (Adhere App)

System Development Process

Previously, we described a five-step process to design and develop a standalone application (app) for patient blood pressure (BP) monitoring and physician's assistance. This app is currently undergoing clinical trial. In this study, we changed the last two steps of the previously described process. After a successful clinical trial (WHO ICRTP registry, ID: KCT0004754), we will evaluate the system. In this study, we developed an app with an additional functionality for monitoring the patient's physical activities. The primary functionalities of the system include patient BP and activity monitoring, medication adherence, and recommendations along with alert generation. The developed apps include a BP&Activity monitoring mobile app, electronic case report form (eCRF)-atrial fibrillation (AF), and Rule Editor. The primary users of the mobile app are registered patients, while the other two apps (eCRF-AF and Rule Editor) are mainly used by physicians to monitor the associated patients' activities and for knowledge creation, respectively. The aforementioned apps were designed and developed based on the five-step process. Supplemental Figure 1 shows the complete system design and development process.

For peer review only

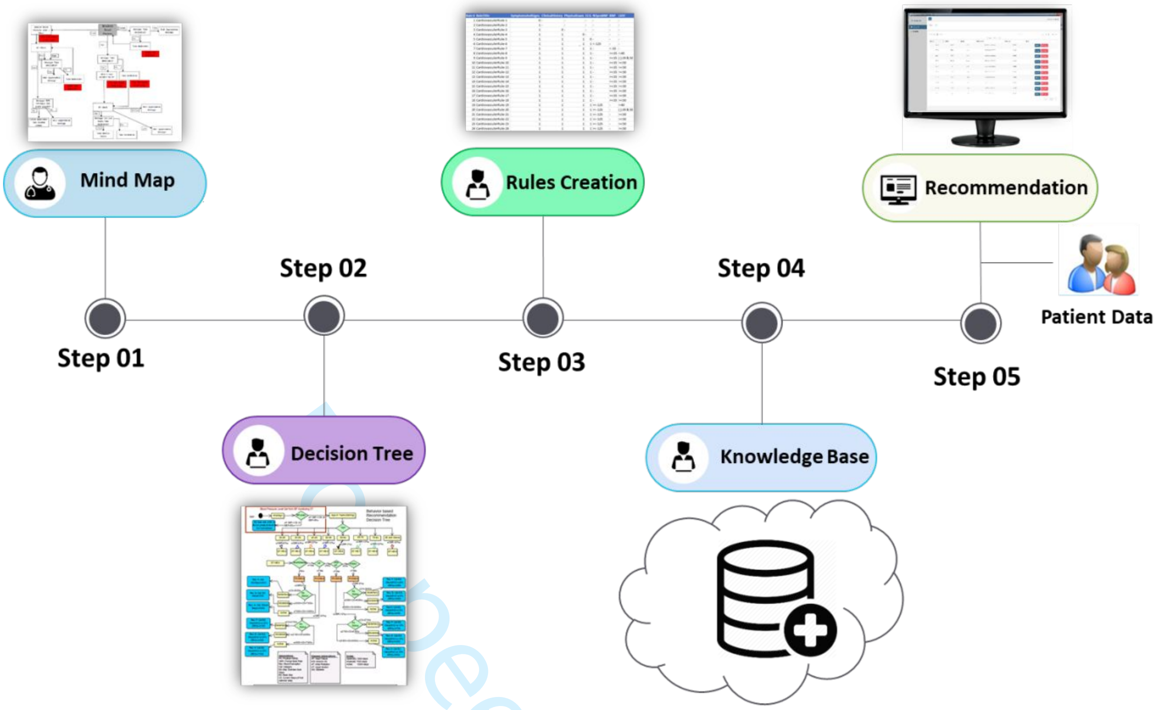

Supplemental Figure 1: System design and development process

- **Mind Map:** First, we represented the physicians' knowledge in an informal format, called the mind map, that is easily understandable to a human but not understandable to a computer. The physicians' knowledge about BP, physical activities, and their correlation was represented in the form of mind map nodes, and the nodes were related among each other based on the conditional relationships. With this analogy, the BP is linked with the age of the patient to identify the recommended activity for the patient. The resultant recommendations about the BP (systolic and diastolic), age, and current status of patient activity are connected to further diagnosis and then treatment of the patient's current condition. In short, physicians' knowledge based on their experience during real practice is represented in the mind map. We modify the initial mind map based on discussion with concerned physicians during a knowledge elicitation phase, and the final mind map is produced after a few knowledge elicitation meetings.
- **Iterative Decision Tree:** In the second phase, we transform the non-formal representation of the mind map into a more formal representation, known as the Iterative Decision Tree (IDT) [1]. The IDT representation is more understandable for physicians and computers; however,

transformation is needed for execution. In the IDT, the contributing factors are highlighted and linked with different tests to cover all the possible paths to reach diagnostic and treatment decisions. In this study, we recommend suitable physical activities to the patients based on their ages. Therefore, the patient first checks their BP, and then the system will generate a recommendation based on the recorded BP and the patient's age. Exercise goal steps (EGS) are calculated and recommended by the system to the patient to achieve the specified goal. Physicians' knowledge regarding BP is correlated with the physical activity, as shown in Supplemental Figure 2. The physicians verified and improved the IDT for all possible cases of that disease in multiple iterations of the knowledge elicitation process.

For peer review only

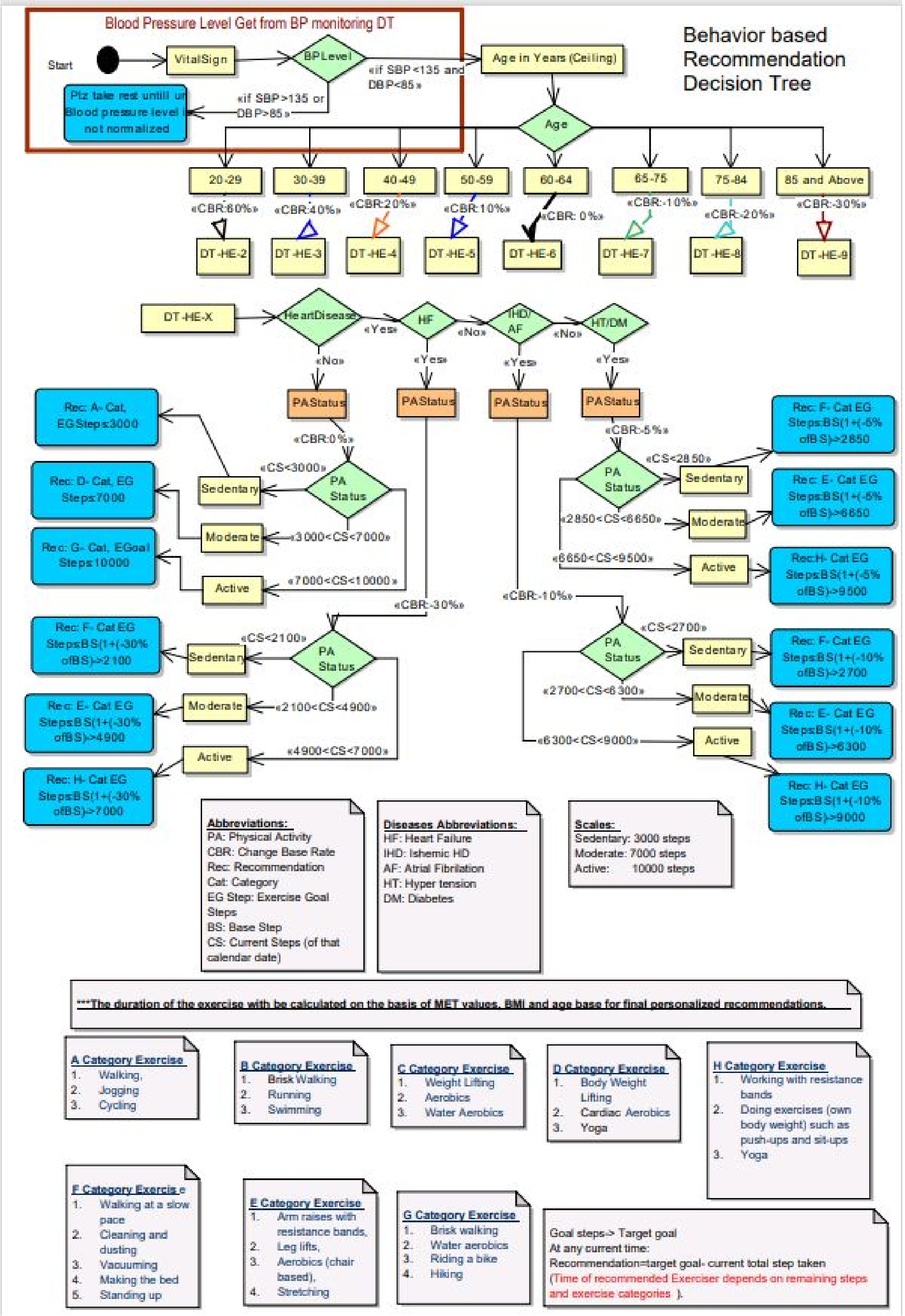

Supplemental Figure 2: Final Iterative Decision Tree (IDT) for blood pressure with physical activity

- **Rule Creation:** As previously mentioned, the IDT representation needs to be transformed into an executable format for the operative recommendations. Therefore, in the rule creation process, we transform the IDT into production rules. The production rules in object-oriented knowledge

bases are an easy way for knowledge execution. In this step, all the IDT branches are transformed into rules by top-down and left-right traversing and reasoning approaches. Our developed knowledge environment, called the Intelligent-Knowledge Authoring Tool (I-KAT), is used to transform rules into production and executable formats.

- **Knowledge Maintenance:** The knowledge maintenance phase provides a sophisticated method for validation and verification of the knowledge base. The transformed rules in the previous step are retrieved for validation and verification using a rule-based reasoning methodology. This aims to find conflict and duplication in the existing rules, if they exist. In this phase, all the rules are stored in the knowledge base in the proper hierarchy of knowledge representation.
- **Recommendation:** The recommendation phase is needed to give recommendations based on the knowledge created in the previous step. Therefore, an app is developed to provide an executable environment, which consists of a rule-based reasoner and a user-friendly interface to handle the input of signs, symptoms, history, and medical test reports to produce a final diagnosis and treatment. A mobile app was developed for BP and physical activity monitoring interventions to support patients. The app prompts the patient to monitor their BP and asks about medication intake. Based on their BP condition and medication intake status, the app recommends a suitable physical activity to perform on right time. If the condition is severe, the app recommends approaching the hospital urgently.

System Architecture and Structure

Supplemental Figure 3 shows the architecture and holistic structure of the system, which consists of four core components: (I) BP&Activity monitoring app, (II) knowledge acquisition and reasoning, (III) database, and (IV) eCRF-AF.

For peer review only

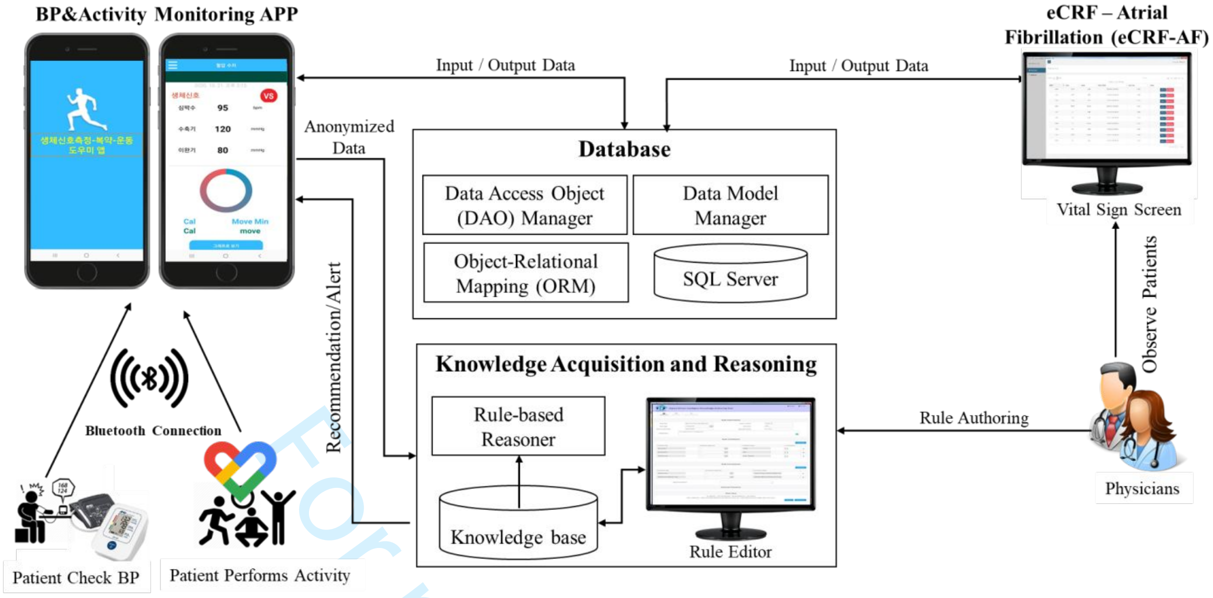

Supplemental Figure 3: System architecture and structural workflow

For peer review only

I. **BP&Activity Monitoring App:** This mobile app is equipped with different functionalities, such as BP and physical activity monitoring, user authentication, user registration, user exercise preferences, dashboard, BP monitoring graph, step-count graph, and recommendation screens. The core screen of the BP&Activity monitoring app is the dashboard, which facilitates users to connect with the BP monitoring device, such as the Bluetooth-enabled BP monitor UA-651BLE (A&D medical, Sydney, Australia). The dashboard screen is responsible for connecting the app with a nearby A&D PB monitoring device via Bluetooth. Similarly, the dashboard has implicit services to count the patients' steps while walking, running, jogging, and performing other physical activities. When the users check their BP, the calculated systolic BP (SBP), diastolic BP (DBP), and heart rate (HR) are sent to the mobile app. The app activates the reasoner to provide recommendations based on the calculated values. In this study, the app also considers patient age and current behavior regarding physical activity.

II. **Knowledge acquisition and reasoning:** In this component, our system provides a knowledge creation environment and reasoning capability. The I-KAT is equipped with a Rule Editor, which facilitates the creation, modification, and management of the knowledge base by physicians. The knowledge base is a core component of the clinical decision support system to

store and maintain clinical knowledge. We are presenting an authoring environment called a Rule Editor that provides an easy-to-use interface for physicians to create clinical knowledge rules. The tool reduces the burden of memorizing and understanding the data model and vocabulary concepts for the physician because the system is equipped with intelli-sense functionality during rule creation. The reasoning capability also handles knowledge validation and verification to find duplicated and conflicted rules within the knowledge base. Finally, the rules persist in the knowledge base. When it receives a patient's recorded BP, the rule-based reasoner is activated to generate recommendations and alerts based on the recorded BP.

III. **Database:** We designed and implemented the database schema to maintain patient demographic data, vital signs (systolic BP, diastolic BP, and HR), physical activity data, and other patient-related data in the database. We implemented two types of database storage: the one for the mobile app is a lightweight database using SQL Lite, which is the default temporary storage for patient data on the smartphone, when the device is not connected to the internet. When the device is connected to the internet, the data are sent to an SQL server for long-term (permanent) storage. Three internal components, data model manager, data access object (DAO) manager, and object relational mapping (ORM) work collaboratively to perform the persistence functionality. The data model manager is responsible for managing the database schema along with data instances to store in the appropriate model. The data access object (DAO) manager is used to access the data objects of the recorded values and prepare the data model to store in the database. The object relational mapping (ORM) is used to convert data between incompatible type systems using object-oriented programming languages. The App Server/Rest API is designed and developed for storing patient information, with patient consensus, for tele-monitoring by the concerned physicians. The patient data are anonymized during communication and persistence. Therefore, only the authorized and concerned physicians can see and analyze only their assigned patients' data.

IV. **eCRF–atrial fibrillation**: Generally, the eCRF system collects patient data during clinical trials. The eCRF system usually collects patient vital sign information. SBP, DBP, and HR are

For peer review only

important vital signs that are managed within the eCRF system. We designed and developed a special purpose eCRF for patients with AF, called eCRF-AF to monitor vital signs (SBP, DBP, and HR) along with physical activities of patients. Therefore, the BP&Activity monitoring app sends the SBP, DBP, and HR and the step counts along with physical activities (sitting, walking, running, jogging, etc.) to the vital sign and physical activity repositories, respectively. These are shown to the physicians on separate analytical screens depending on the nature of the data. The eCRF-AF systems and HMIS systems of different hospitals can be integrated with the BP&Activity monitoring app. We focused and integrated the BP&Activity monitoring app with a lightweight eCRF-AF system developed for managing clinical trials. Physicians can easily observe the BP trends (daily, weekly, monthly, yearly, etc.) along with exercise, using physical activity trends of the concerned patients. If the physicians find an abnormality in the patient data, they will communicate with the respective patient.

System Implementation Outcome: Process Flow

The system comprises three main apps: one is a mobile app (BP&Activity monitoring app), which is a patient-oriented app, while the other two are web apps (eCRF-AF and Rule Editor), which are physician-oriented apps. Here, we focus on the patient-oriented BP&Activity monitoring app, which comprises 11 different screens with multiple functionalities. The BP&Activity monitoring app and its utilization and process flow are depicted in **Supplemental Figure 4**. The BP&Activity monitoring app has two processes: one process for new user registration and a second process for registered patients to check their BPs and physical activities to obtain suitable and correct recommendations based on the recorded BP, age, profile questionnaire, and exercise preferences.

User Registration Process: To register a new user on the system, we follow a three-step process to obtain user information.

- **Step 1:** The user will add profile information, such as name, gender, date of birth, and email address, and other information to register on the system.
- **Step 2:** The questionnaire screen is used to investigate physical behavior in terms of type of

For peer review only

exercise, duration of exercise, and frequency of exercise. All these questions help to calculate the patients' current behavior in terms of physical activity.

- **Step 3:** The preferences screen gives privileges to the user to select the appropriate exercises from the recommended ones to set the final recommendation as a preference.

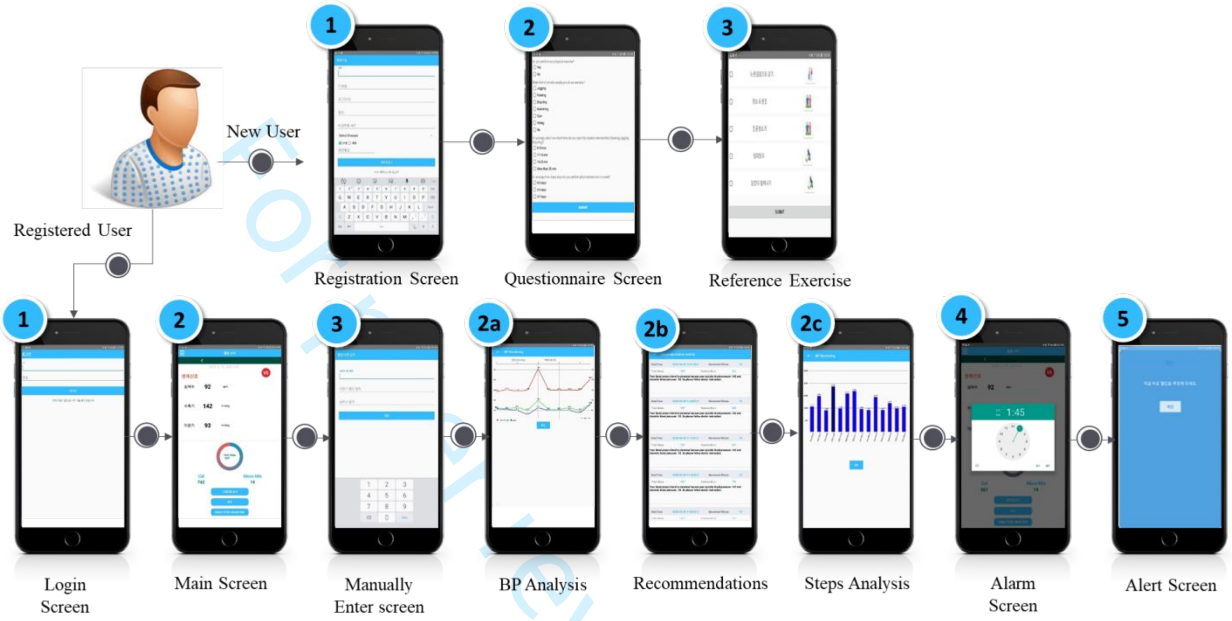

Supplemental Figure 4: Utilization and process flow of BP&Activity monitoring app (Patient-Oriented)

BP and Activity Monitoring Process: After successful registration, the patient can utilize the app to monitor their vital signs (SBP, DBP, and HR) along with their physical activity status through an A&D BP monitoring device and GoogleFit API.

- **Step 1:** The user needs to login to the system with his/her credentials for authentication.
- **Step 2:** After successful authentication, the user can access a dashboard screen. It consists of the current status of their vital signs along with the step count. It also facilitates observation of analytics and recommendation history.
- **Step 3:** The user can enter vital sign details manually when the attached device is not communicating properly. The app accepts SBP, DBP, and HR values.

- **Step 2a:** From the dashboard, the user can visualize historical analytics for vital signs, which represent the health status in terms of BP and HR.
- **Step 2b:** From the dashboard, the user can visualize historical recommendations regarding exercises based on their physical behavior with respect to time.
- **Step 2c:** From the dashboard, the user can visualize step count historical analytics, with respect to the day, to understand their own physical behavior.
- **Step 4:** The alarm screen facilitates the user to decide and set an alarm for checking their BP. At the selected time, the system will generate the alarm for the user to check their BP and take their medication, as necessary.
- **Step 5:** The system generates recommendations at different stages of BP and activity monitoring based on patient preferences, age, and answers to the questionnaire. The recommendation may issue a message to check BP, walk, run, jog, and rest for a specified time, or to take medication at the specified time.

For peer review only

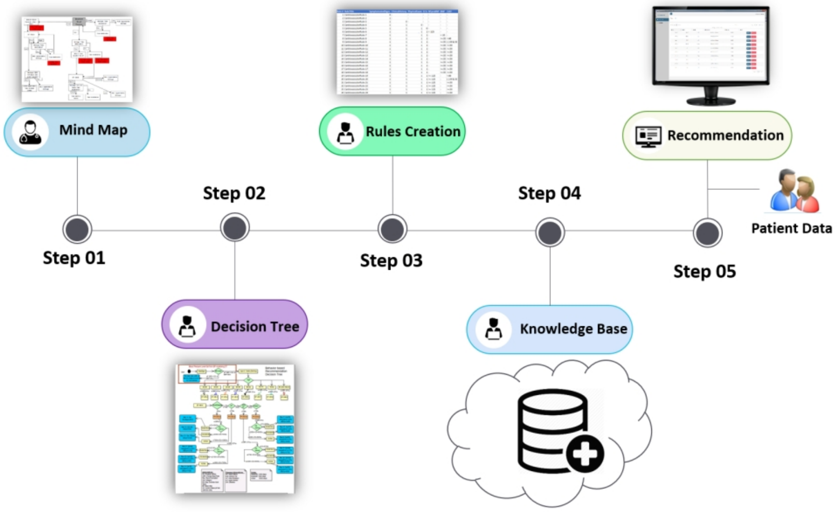

311x169mm (150 x 150 DPI)

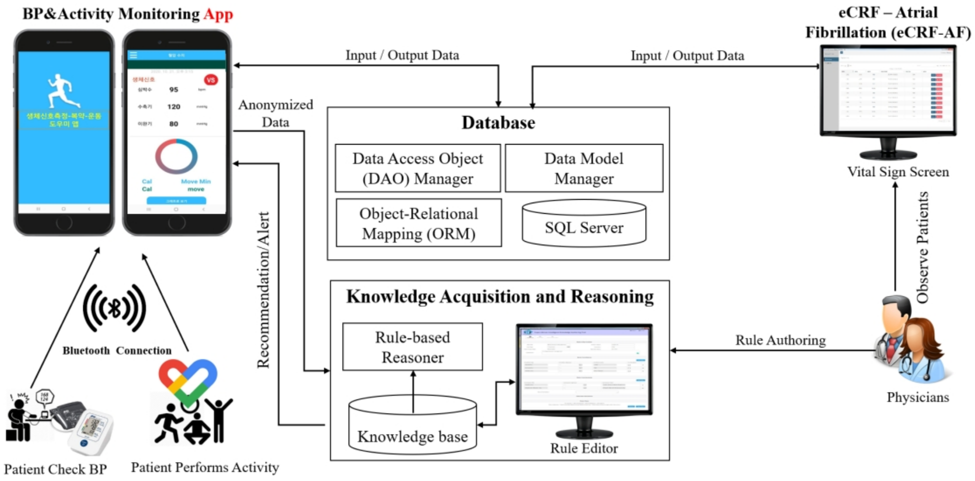

338x172mm (150 x 150 DPI)

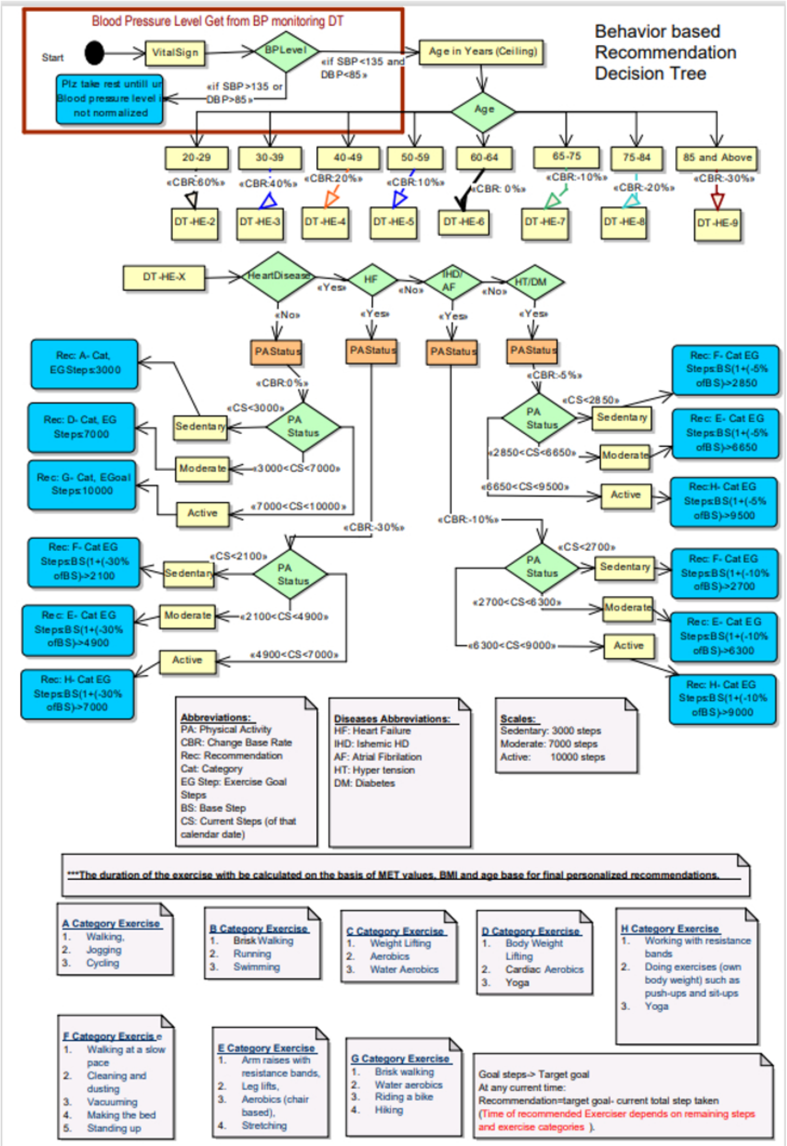

141x187mm (150 x 150 DPI)

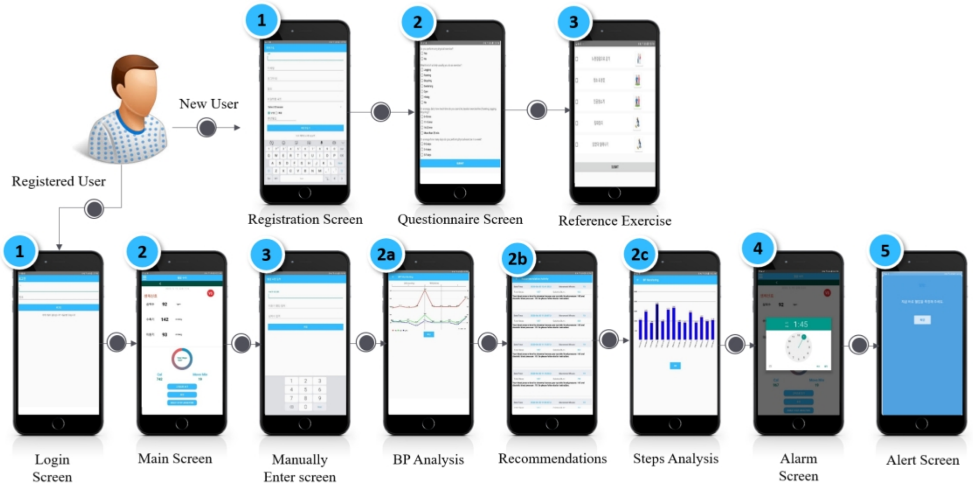

338x182mm (150 x 150 DPI)

BMJ Open

Rationale, Design, and Efficacy of a Smartphone Application for Improving Self-Awareness of Adherence to Edoxaban Treatment: A Randomized Controlled Study (Adhere App)

Journal:	*BMJ Open*
Manuscript ID	bmjopen-2021-048777.R1
Article Type:	Protocol
Date Submitted by the Author:	26-Jul-2021
Complete List of Authors:	Kim, In-Cheol; Keimyung University Dongsan Hospital,<br>Lee, Ji Hyun ; Bundang Seoul National University Hospital Department of Internal Medicine, Cardiology<br>Choi, Dong-Ju; Seoul National University Bundang Hospital, Department of Internal Medicine<br>Park, Sung-Ji ; Samsung Medical Center, Division of Cardiology<br>Lee, Ju-Hee; Chungbuk National University College of Medicine, Department of Internal Medicine<br>Park, Sang Min; Chuncheon Sacred Heart Hospital<br>Kim, Mina; Korea University Anam Hospital<br>Kim, Hack-Lyoung; Seoul National University Seoul Metropolitan Government Boramae Medical Center, Internal Medicine<br>Lee, Sunki; Hallym University College of Medicine<br>Kim, In Jai; CHA University, Department of Internal Medicine<br>Choi, Seonghoon; Kangnam Sacred Heart Hospital<br>Bang, Jaehun; Kyung Hee University, Department of Computer Science and Engineering<br>Ali, Bilal; Kyung Hee University, Department of Computer Science and Engineering<br>Hussain, Musarrat; Kyung Hee University, Department of Computer Science and Engineering<br>Ali, Taqdir; Kyung Hee University, Department of Computer Science and Engineering<br>Lee, Sungyoung; Kyung Hee University, Department of Computer Science and Engineering
**Primary Subject Heading**:	Cardiovascular medicine
Secondary Subject Heading:	Medical management, Health informatics
Keywords:	MEDICAL EDUCATION & TRAINING, Cardiology < INTERNAL MEDICINE, Stroke < NEUROLOGY

Rationale, Design, and Efficacy of a Smartphone Application for Improving Self-Awareness of Adherence to Edoxaban Treatment: A Randomized Controlled Study (Adhere App)

In-Cheol Kim[1], Ji Hyun Lee[2], Dong-Ju Choi[2], Sung-Ji Park[3], Ju-Hee Lee[4], Sang Min Park[5], Mina Kim[6], Hack-Lyoung Kim[7], Sunki Lee[8], In Jai Kim[9], Seounghoon Choi[10], Jaehun Bang[11], Bilal Ali[11], Musarrat Hussain[11], Taqdir Ali[11], and Sungyoung Lee[11]

1) Division of Cardiology, Department of Internal Medicine, Keimyung University Dongsan Hospital, Keimyung University School of Medicine, Daegu, Korea

2) Division of Cardiology and Cardiovascular Center, Department of Internal Medicine, Seoul National University Bundang Hospital, Seongnam, Korea

3) Division of Cardiology, Department of Internal Medicine, Heart Vascular Stroke Institute, Samsung Medical Center, Sungkyunkwan University School of Medicine, Seoul, Korea.

4) Division of Cardiology, Department of Internal Medicine, Chungbuk National University Hospital, Chungbuk National University College of Medicine, Cheongju, Korea

5) Department of Cardiology, Chuncheon Sacred Heart Hospital, Hallym University, Chuncheon, Korea

6) Department of Cardiology, Korea University College of Medicine, Seoul, Korea

7) Division of Cardiology, Seoul National University Boramae Medical Center, Seoul, Korea

8) Division of Cardiology, Dongtan Sacred Heart Hospital, Hallym University College of Medicine, Gyunggi-do, Korea

For peer review only

9) Division of Cardiology, Department of Internal Medicine, Bundang CHA Medical Center, CHA University, Seongnam, Korea

10) Division of Cardiology, Department of Internal Medicine, Kangnam Sacred Heart Hospital, Hallym University College of Medicine, Seoul, Korea

11) Department of Computer Science and Engineering, Kyung Hee University, Yongin, Korea

* IC Kim and JH Lee contributed equally to this work

Word counts

Abstract: 301 words

Main text (excluding title page, abstract, references, figures, and tables): 2,352 words

Correspondence

Dong-Ju Choi, M.D. Ph.D. (https://orcid.org/0000-0003-0146-2189)

Division of Cardiology and Cardiovascular Center, Department of Internal Medicine, Seoul National University Bundang Hospital, Seongnam, Korea.

82, gumi-ro 173 Beon-gil, Bundang-gu, Seongnam, Gyeonggido, 13620, Korea

Tel: 82-31-787-7007, Fax: 82-31-787-4051

E-mail: djchoi@snubh.org

For peer review only

ABSTRACT

Introduction: High adherence to oral anticoagulants is essential for stroke prevention in patients with atrial fibrillation (AF). We developed a smartphone application (app) that pushes alarms for taking medication and measuring blood pressure (BP) and heart rate (HR) at certain times of the day. In addition to drug alarms, the habit of measuring one's BP and HR may reinforce drug adherence by improving self-awareness of the disease. This pilot study aims to test the feasibility and efficacy of the smartphone app-based intervention for improving drug adherence in patients with AF.

Methods and analysis: A total of 10 university hospitals in Korea will participate in this randomized control trial. Patients with AF, being treated with edoxaban for stroke prevention will be included in this study. Total of 500 patients will be included and the patients will be randomized to the conventional treatment group (250 patients) and the app conditional feedback group (250 patients). Patients in the app conditional feedback group will use the medication reminder app for medication and BP check alarms. The automatic BP machine will be linked to the smartphone via Bluetooth. The measured BP and HR will be updated automatically on the smartphone app. The primary endpoint is edoxaban adherence by pill count measurement at three and six months of follow-up. Secondary endpoints are clinical composite endpoints including stroke, systemic embolic event, major bleeding requiring hospitalization or transfusion, or death during the six-months. As of 6 November 2020, patient enrollment had not started.

Ethics and dissemination: This study was approved by the Seoul National University Bundang Hospital Institutional Review Board (B-1904/532-301) and will be conducted according to the principles of the Declaration of Helsinki. The study results will be published in a reputable journal.

Trial registration: WHO ICRTP registry, ID: KCT0004754. As of 6 November 2020, recruitment had not started.

Key words

atrial fibrillation; drug adherence; smartphone application; edoxaban

For peer review only

Strengths and limitations of this study.

- This randomized control trial investigates the feasibility and efficacy of a smartphone app-based intervention for improving adherence to edoxaban in multiple centers.
- This trial will unveil whether self-care behavior can improve disease awareness which might further reinforce drug adherence.
- The sample size was calculated to show the difference in drug adherence, and not for any clinical events.
- The study results may not be applicable to patients who are not capable of using smartphones.

For peer review only

INTRODUCTION

Stroke prevention with oral anticoagulants is crucial in the management of atrial fibrillation (AF).[1] the Large randomized control trials with AF patients have consistently shown comparable effectiveness and improved safety of non-vitamin K oral anti-coagulants (NOAC) compared with warfarin.[2-6] Based on these trials, the current global guidelines recommend NOACs over warfarin for stroke prevention in patients with AF.[1 7 8]

In contrast to warfarin, NOACs have predictable effectiveness and minimal drug interactions. Consequently, regular blood tests for the monitoring of anticoagulation effects are not necessary with NOAC therapy. The use of NOACs is increasing worldwide because of these advantages.[9 10] However, there are some concerns regarding the relatively short half-life on NOACs. When doses are missed, a prothrombotic status might adversely triggered.[11] Thus, in NOAC therapy, consistent drug adherence is essential to maintain a consistent anticoagulation effect for stroke prevention. Poor drug adherence or failed persistence would increase the risk of stroke, and this may lead to an increase in public health care costs.[12]

Even though there are controversies regarding their efficacy, smartphone applications (apps) are currently being recognized as an effective tool for promoting drug adherence[13-23]. Smartphone apps send notifications or push alarms reminding the patient to take their medication. Moreover, active involvement in measuring their health status – blood pressure (BP) and heart rate (HR) using smartphone app-based feedback system could improve awareness of their underlying condition. This in turn influences their self-care behavior and increases drug adherence[24]. The aim of this study protocol is to investigate the feasibility and efficacy of a smartphone app-based intervention that provides push alarms for taking medication and measuring BP and HR in patients with AF requiring oral anticoagulation therapy.

For peer review only

METHODS AND ANALYSIS

Study design

The Adhere-App (Self-awareness of Drug Adherence to Edoxaban Using an Automatic App. Feedback System) study is a multicenter, randomized, open-label, and group-comparison trial to assess the effect of using a smartphone app to improve drug adherence (**Figure 1**). A total of 10 tertiary university hospitals in Korea will participate in this study. The study participants will be randomized to a smartphone app-conditioned feedback group or a conventional treatment group. Every patient will receive standardized education on AF and the importance of anticoagulation therapy. Additionally, the intervention group will be educated on the use of the smartphone app coupled with BP and HR measurement using an automatic BP measuring machine. Study visits will be performed at baseline, three months, and six months. When clinical visits are not possible, the patient's condition and survey data can be collected by telephone visit. This study was registered in the International Clinical Trials Registry Platform (ICTRP) on 20 February 2020. However, initiation was delayed due to coronavirus disease 2019 (COVID-19) pandemic. As of 6 November 2020, patient enrollment is expected to be initiated in a few weeks and is expected to be completed in late 2022.

Study participants

Adult patients (≥ 19 years old) with non-valvular AF requiring oral anticoagulation therapy (CHA_2DS_2-VASC score ≥ 2 points) for stroke prevention will be enrolled. Edoxaban (Lixiana®, Daiichi Sankyo, Tokyo, Japan) will be administered to patients at an on-label dosage of 60 mg once daily. The dosage will be reduced to 30 mg once daily in patients who meet any of the following criteria: moderate renal impairment (creatinine clearance 30-50 mL/min), body weight less than or equal to 60 kg, or concomitant use of potent P-glycoprotein inhibitors.

Since smartphone usage is crucial in this study, participants should be able to use smartphone well, and be capable of following instructions on how to use the app in Korean language. Patients with severe

For peer review only

renal insufficiency (creatinine clearance < 15 mL/min) and significant mitral valve disease will be excluded. The detailed inclusion and exclusion criteria for the Adhere-App study are listed in **Table 1**.

Table 1. Key inclusion and exclusion criteria for Adhere-App study.

Inclusion criteria<br><br>• Patients with atrial fibrillation, treated with edoxaban for stroke prevention<br>• Age ≥ 19 years<br>• Capable of using smartphone application<br>• Accepted for the study protocol and willing to participate in the clinical study
Exclusion criteria<br><br>• Creatinine clearance less than 15 mL/min<br>• Patients with dual antiplatelet therapy<br>• Moderate or severe mitral stenosis<br>• Previous history of mitral valve replacement or mitral valve repair<br>• Previous history of alcohol or drug abuse<br>• Not suitable for the clinical trial enrollment by the judgement of the investigator<br>• History of previous enrollment in another clinical trial using an investigational pharmaceutical product<br>• Patient unwilling to participate in the clinical study

For peer review only

At baseline, age, sex, details of comorbidities, and laboratory findings will be assessed for all patients. The CHA_2DS_2-VASc score for stroke risk prediction is calculated by the summation of all assigned points for each particular medical condition: one point each for age between 65 and 74 years (A), female sex (Sc), hypertension (H), diabetes mellitus (D), congestive heart failure (C), and vascular disease (V, prior myocardial infarction or peripheral artery disease) and 2 points each for a history of stroke/transient ischemic attack/thromboembolism (S_2) or age of ≥ 75 years (A_2).[25]

Patient and public involvement

Patients or the public will not be involved in the design, reporting, or dissemination of our research.

Patient recruitment and randomization

Potential participants will be any patients visiting the clinic for AF management. After a comprehensive interview, written informed consent will be obtained from all eligible participants. Eligible participants will be asked to complete a baseline survey covering demographics and cardiovascular comorbidities. The eligible participants will then be randomly assigned to either the intervention group or the conventional treatment group in a 1:1 ratio using computer generated random list. (**Figure 2**). Due to the nature of the intervention, it is not possible to blind participants to group allocation. All participants will be informed that the information on edoxaban adherence will be collected for analysis.

Intervention (Smartphone app-conditioned feedback)

Study participants in the intervention group will install the study app provided by clinical research coordinators on their own smartphone. The operating system and sample display of the smartphone app are summarized in **Figure 3**. The participants can set the alarm for taking edoxaban at a certain time of

For peer review only

the day. The research coordinator or the physician will set the alarm initially. Patient's exercise information, including interval, exercise type, and duration, will be submitted on the first visit. When the alarm sounds at the pre-specified time, the patient will be asked to take the drug (edoxaban) and measure their BP and HR using an automated electronic manometer (UA-651BLE Bluetooth Blood Pressure monitor, A&D Medical, Sydney, Australia). The measured BP and HR data will be automatically transmitted to the smartphone via Bluetooth, as shown in **Supplemental Figure 1, 2, 3 and 4**. To evaluate awareness of disease status, the app will ask whether the measured BP result is optimal, and the patient is required to respond to the question. The app will also check whether the patient took the medication. If the patient does not affirm taking the medication, the app will send an alert message to ensure that the patient takes the medication. The data regarding the time of edoxaban intake, BP, and HR will be automatically updated and stored daily. Finally, the physician can obtain feedback on BP and HR measurement data and drug adherence by checking the app on the follow-up visit. The aforementioned functionalities will be provided by the smartphone app, while a standalone web application has been developed for physicians to monitor associated patients' BP, physical activity, and medication adherence trends. The design, development, and utilization detail of the patient-oriented smartphone app and physician-oriented web app are described in the **Supplemental Materials**.

Control (Conventional treatment)

In the control group, only standard guideline-recommended treatments, including education on the disease and the importance of taking medications will be covered. No other specific interventions will be made. Patients will be followed up in clinics according to the study protocol (three months and six months) and be provided standard care for AF.

Study endpoints and follow up

For peer review only

The primary endpoint is edoxaban adherence by pill count measurement at second (three months) and third (six months) visits. All patients will be asked to bring their remaining pills at each clinical visit. Secondary endpoints are clinical composite endpoints, including stroke, systemic embolic event, major bleeding requiring hospitalization or transfusion, or death during the six months of follow up. The primary and secondary endpoints will be compared between Smartphone app-conditioned feedback group and control group. An additional pre-defined observational parameter is symptom change related to BP (both systolic and diastolic) and HR. Clinical research coordinators will ask symptoms as an open question and each investigator will determine whether the symptom is related with BP and HR change.

Sample size calculation

This is a pilot study to assess the efficacy of a smartphone-based intervention. To date, there has been no study evaluating the use of a smartphone app to improve NOAC adherence in Korea. Thus, a precise sample size calculation was not available. We assumed that drug adherences would be 90% in the control group and 95% in the intervention group. A sample size of 234 patients in each group achieves 95% power to detect a mean difference of 5.0 with a known standard deviation of differences of 15.0 and a significance level (alpha) of 0.05, using a two-sided paired z-test. Considering dropout rate of 4%, we finally planned to enroll 500 patients (250 patients in each group). We expect this pilot study will provide valuable information regarding the appropriate sample size, recruitment rate, study period and data management process etc., for assessing the feasibility of the full-scale study we are planning further. And it also would allow to identify potential practical problems of whole research process.

Statistical analysis

Categorical variables will be presented as numbers and frequencies, whereas continuous variables will be presented as means ± standard deviations. The student's t-test and Kruskal-Wallis test will be used to compare continuous variables depending on the presence of normally distributed variables. The chi-

For peer review only

square test will be used to compare categorical variables. A p-value < 0.05 was considered significant. The analysis would be performed according to the intention-to-treat principle. The standard regimen of edoxaban is once daily. The primary endpoint of edoxaban adherence will be calculated by pill count as shown below.

$$\text{Pill count adherence (\%)} = \frac{\text{Number of pills dispensed} - \text{Number of pills remaining}}{\text{Number of days between dispensing date and follow up date}} \times 100$$

Since new drug users might have a lower adherence than continuous users, subgroup analysis will also be performed on the new and continuous users. To evaluate the feasibility and efficacy of the smart phone application, further analysis can be performed according to the frequency of the smart phone application use. The secondary endpoints of adverse events will be compared with the Kaplan-Meier curve analysis. A log rank test will be used to evaluate the significance of the difference between the two groups. Statistical analysis will be performed using IBM SPSS Statistics for Windows (IBM Corp., Armonk, N.Y., USA) and R software version 4.0.3.

Ethics and dissemination.

This study was approved by the institutional review board of Seoul National University Bundang Hospital and will be conducted according to the principles of the Declaration of Helsinki. The potential risks to participants are negligible in the present study. The participants will be handed study information sheets and asked to provide written informed consent. The study results will be disseminated via publication in a reputable journal and presentation at scientific meetings. Individual participant data that underlie the results reported (text, tables, figures and appendices) will be shared after deidentification process upon reasonable request.

DISCUSSION

This randomized control trial investigates the feasibility and efficacy of using a smartphone app to improve adherence to anticoagulation therapy (edoxaban). Furthermore, this study will also assess the

For peer review only

influence of app-based interventions influences on the reduction of AF-related adverse clinical events. Today, smartphones have became an integral part of everyday life. We believe that our study will be helpful when formulating public health strategies for improving NOAC adherence using smartphones.

All participants in the intervention group will be asked to input the measured BP and HR data on a smartphone, in addition to responding to a push alarm for taking their medication. The recognition of the irregular rhythm of AF during heart rate measurement could remind the participants of the necessity of anticoagulation therapy, which they will be informed about by their physician during the education session. Feedback questions to remind the patient of their BP control status might further reinforce disease awareness and drug adherence. The stored information could also be used as a reference to set an optimal therapeutic goal for education and medication control.

A similar study was performed with NOACs, using a telemonitoring system accompanied by personal telephone feedback.[12] In that study, although the telemonitoring system achieved high drug adherence (over 90%), it was not cost-effective. The human resource and system set-up costs to operate telemonitoring-based feedback impeded the wide usage of this method in clinical practice. Digital interventions using smartphone apps are becoming an increasingly common way to support medication adherence and self-management in chronic disorders.[26] Smartphones also help to improve access to cardiac rehabilitation and heart failure management for patients unable to attend traditional center-based cardiac rehabilitation[27] We believe that our strategy of using a smartphone app would be an effective way to increase drug adherence at a minimum cost.

As with other current management strategies, apps can evolve into tailored programs. Besides the simple reminders, this strategy can be incorporated into a multimodal strategy that results in sustained improvements in adherence.[28] Reminders primarily focus on unintentional nonadherence, identifying the reasons for nonadherence and developing a scale that assesses unintentional nonadherence.[29-31] This randomized trial will not only evaluate the effectiveness of the reminder app on disease awareness and drug adherence in patients with AF but will also try to find components that may hinder patient adherence to NOACs. This information could be used to develop a strategy to ensure sustained

For peer review only

improvement in adherence to current medical therapy.

Strengths and limitations

Several limitations of this study should be addressed. The study participants do not represent the general population with AF. All participants should be able to use smartphone apps without difficulties. Thus, the results of the study may not be applicable to patients who are not capable of using smartphones, such as the extreme elderly. Second, the study participants who agree to participate in the study might have a higher interest in their own health. Consequently, higher adherence to drug therapy might be observed than that of the general AF population. Third, the sample size was calculated to show the difference in drug adherence. It is not powered to show any differences in clinical events. Despite these limitations, a major strength is that the present study is a multicenter randomized control trial with a relatively large number of participants.

Author Contributions

DJC, ICK, and JHL contributed to the conception of the study. DJC, ICK, JHL, and MK participated in the design of the study. ICK and JHL drafted and finalized the manuscript. SJP, JHL, SMP, MK, HLK, SL, IJK, SC, JB, BA, MH, TA, SL and DJC revised the manuscript critically for important intellectual content.

Funding statement

This work was supported by a grant from Daiichi Sankyo, Korea (06-2019-233). The funders have no influence on the study design, data collection or the decision to submit reports for publication.

For peer review only

Competing interests statement.

None declared.

For peer review only

References

1. Hindricks G, Potpara T, Dagres N, et al. 2020 ESC Guidelines for the diagnosis and management of atrial fibrillation developed in collaboration with the European Association of Cardio-Thoracic Surgery (EACTS). *Eur Heart J* 2020 doi: 10.1093/eurheartj/ehaa612

2. Ruff CT, Giugliano RP, Braunwald E, et al. Comparison of the efficacy and safety of new oral anticoagulants with warfarin in patients with atrial fibrillation: a meta-analysis of randomised trials. *Lancet* 2014;383(9921):955-62. doi: 10.1016/S0140-6736(13)62343-0

3. Connolly SJ, Ezekowitz MD, Yusuf S, et al. Dabigatran versus warfarin in patients with atrial fibrillation. *N Engl J Med* 2009;361(12):1139-51. doi: 10.1056/NEJMoa0905561

4. Patel MR, Mahaffey KW, Garg J, et al. Rivaroxaban versus warfarin in nonvalvular atrial fibrillation. *N Engl J Med* 2011;365(10):883-91. doi: 10.1056/NEJMoa1009638

5. Granger CB, Alexander JH, McMurray JJ, et al. Apixaban versus warfarin in patients with atrial fibrillation. *N Engl J Med* 2011;365(11):981-92. doi: 10.1056/NEJMoa1107039

6. Giugliano RP, Ruff CT, Braunwald E, et al. Edoxaban versus warfarin in patients with atrial fibrillation. *N Engl J Med* 2013;369(22):2093-104. doi: 10.1056/NEJMoa1310907

7. January CT, Wann LS, Calkins H, et al. 2019 AHA/ACC/HRS Focused Update of the 2014 AHA/ACC/HRS Guideline for the Management of Patients With Atrial Fibrillation: A Report of the American College of Cardiology/American Heart Association Task Force on Clinical Practice Guidelines and the Heart Rhythm Society in Collaboration With the Society of Thoracic Surgeons. *Circulation* 2019;140(2):e125-e51. doi: 10.1161/CIR.0000000000000665 [published Online First: 2019/01/29]

8. Kirchhof P, Benussi S, Kotecha D, et al. 2016 ESC Guidelines for the management of atrial fibrillation developed in collaboration with EACTS. *Eur Heart J* 2016;37(38):2893-962. doi: 10.1093/eurheartj/ehw210 [published Online First: 2016/08/28]

9. Lee SR, Choi EK, Han KD, et al. Temporal trends of antithrombotic therapy for stroke prevention in Korean patients with non-valvular atrial fibrillation in the era of non-vitamin K antagonist oral anticoagulants: A nationwide population-based study. *PLoS One* 2017;12(12):e0189495. doi: 10.1371/journal.pone.0189495 [published Online First: 2017/12/21]

10. Huisman MV, Rothman KJ, Paquette M, et al. The Changing Landscape for Stroke Prevention in AF: Findings From the GLORIA-AF Registry Phase 2. *J Am Coll Cardiol* 2017;69(7):777-85. doi:

For peer review only

10.1016/j.jacc.2016.11.061

11. How CH. Novel oral anticoagulants for atrial fibrillation. *Singapore Med J* 2015;56(12):657-8; quiz 59. doi: 10.11622/smedj.2015184
12. Desteghe L, Vijgen J, Koopman P, et al. Telemonitoring-based feedback improves adherence to non-vitamin K antagonist oral anticoagulants intake in patients with atrial fibrillation. *Eur Heart J* 2018;39(16):1394-403. doi: 10.1093/eurheartj/ehx762 [published Online First: 2018/01/05]
13. Weisman O, Schonherz Y, Harel T, et al. Testing the Efficacy of a Smartphone Application in Improving Medication Adherence, Among Children with ADHD. *Isr J Psychiatry Relat Sci* 2018;55(2):59-63.
14. Morawski K, Ghazinouri R, Krumme A, et al. Association of a Smartphone Application With Medication Adherence and Blood Pressure Control: The MedISAFE-BP Randomized Clinical Trial. *JAMA Intern Med* 2018;178(6):802-09. doi: 10.1001/jamainternmed.2018.0447
15. Huang Z, Tan E, Lum E, et al. A Smartphone App to Improve Medication Adherence in Patients With Type 2 Diabetes in Asia: Feasibility Randomized Controlled Trial. *JMIR Mhealth Uhealth* 2019;7(9):e14914. doi: 10.2196/14914
16. Sarzynski E, Decker B, Thul A, et al. Beta Testing a Novel Smartphone Application to Improve Medication Adherence. *Telemed J E Health* 2017;23(4):339-48. doi: 10.1089/tmj.2016.0100
17. Yoon CH, Ritchie SR, Duffy EJ, et al. Impact of a smartphone app on prescriber adherence to antibiotic guidelines in adult patients with community acquired pneumonia or urinary tract infections. *PLoS One* 2019;14(1):e0211157. doi: 10.1371/journal.pone.0211157
18. Wu YP, Linder LA, Kanokvimankul P, et al. Use of a Smartphone Application for Prompting Oral Medication Adherence Among Adolescents and Young Adults With Cancer. *Oncol Nurs Forum* 2018;45(1):69-76. doi: 10.1188/18.ONF.69-76
19. Yu C, Liu C, Du J, et al. Smartphone-based application to improve medication adherence in patients after surgical coronary revascularization. *Am Heart J* 2020;228:17-26. doi: 10.1016/j.ahj.2020.06.019
20. Himelhoch S, Kreyenbuhl J, Palmer-Bacon J, et al. Pilot feasibility study of Heart2HAART: a smartphone application to assist with adherence among substance users living with HIV. *AIDS Care* 2017;29(7):898-904. doi: 10.1080/09540121.2016.1259454
21. Talmor G, Nguyen B, Keibel A, et al. Use of software applications to improve medication adherence and achieve more integrated disease management in heart failure. *Trends Cardiovasc Med* 2018;28(7):483-88. doi: 10.1016/j.tcm.2018.04.001

For peer review only

22. Johnston N, Bodegard J, Jerstrom S, et al. Effects of interactive patient smartphone support app on drug adherence and lifestyle changes in myocardial infarction patients: A randomized study. *Am Heart J* 2016;178:85-94. doi: 10.1016/j.ahj.2016.05.005

23. Marquez Contreras E, Marquez Rivero S, Rodriguez Garcia E, et al. Specific hypertension smartphone application to improve medication adherence in hypertension: a cluster-randomized trial. *Curr Med Res Opin* 2019;35(1):167-73. doi: 10.1080/03007995.2018.1549026

24. Kelders SM, Kok RN, Ossebaard HC, et al. Persuasive system design does matter: a systematic review of adherence to web-based interventions. *J Med Internet Res* 2012;14(6):e152. doi: 10.2196/jmir.2104 [published Online First: 2012/11/16]

25. Lip GY, Nieuwlaat R, Pisters R, et al. Refining clinical risk stratification for predicting stroke and thromboembolism in atrial fibrillation using a novel risk factor-based approach: the euro heart survey on atrial fibrillation. *Chest* 2010;137(2):263-72. doi: 10.1378/chest.09-1584

26. Morrissey EC, Casey M, Glynn LG, et al. Smartphone apps for improving medication adherence in hypertension: patients' perspectives. *Patient Prefer Adherence* 2018;12:813-22. doi: 10.2147/PPA.S145647 [published Online First: 2018/05/23]

27. Hamilton SJ, Mills B, Birch EM, et al. Smartphones in the secondary prevention of cardiovascular disease: a systematic review. *BMC Cardiovasc Disord* 2018;18(1):25. doi: 10.1186/s12872-018-0764-x [published Online First: 2018/02/09]

28. Haynes RB, Ackloo E, Sahota N, et al. Interventions for enhancing medication adherence. *Cochrane Database Syst Rev* 2008(2):CD000011. doi: 10.1002/14651858.CD000011.pub3

29. Gadkari AS, McHorney CA. Unintentional non-adherence to chronic prescription medications: how unintentional is it really? *BMC Health Serv Res* 2012;12:98. doi: 10.1186/1472-6963-12-98

30. Dayer L, Heldenbrand S, Anderson P, et al. Smartphone medication adherence apps: potential benefits to patients and providers: response to Aungst. *J Am Pharm Assoc (2003)* 2013;53(4):345. doi: 10.1331/JAPhA.2013.13121

31. Dayer L, Heldenbrand S, Anderson P, et al. Smartphone medication adherence apps: potential benefits to patients and providers. *J Am Pharm Assoc (2003)* 2013;53(2):172-81. doi: 10.1331/JAPhA.2013.12202

For peer review only

Figure legends

Fig. 1 Schematic diagram of expected improvement in NOAC adherence by regular measurement of BP, heart rate and smartphone feedback in patients with atrial fibrillation. NOAC, non-vitamin K oral anti-coagulant; BP, blood pressure; HR, heart rate.

Fig. 2 Study design of AdhereApp study. Patients using edoxaban will be randomized to either an application conditioned-feedback group or a conventional therapy group.

Fig. 3 Operating system and sample display of the smartphone app. When the patient measures BP, the data are automatically transferred to the app via Bluetooth system. A, title page; B, menu bar indicating BP measurement, log-out, alarm setting, manual BP input, and password setting; C, alarm setting page for medication; D, exercise information including interval, exercise type, and duration; E, alarm page to check whether the patient has taken medication; F, BP manual input page; G, page displaying measured BP and HR result; H, feed-back question to check whether the patient has achieved optimal target BP. BP, blood pressure; HR, heart rate.

For peer review only

Figure. 1 Schematic diagram of expected improvement in NOAC adherence by regular measurement of BP, heart rate and smartphone feedback in patients with atrial fibrillation. NOAC, non-vitamin K oral anti-coagulant; BP, blood pressure; HR, heart rate.

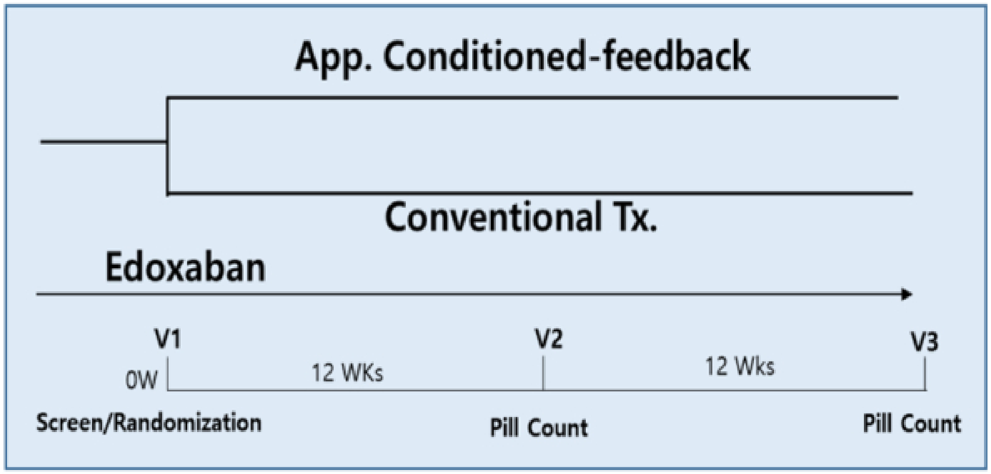

Figure. 2 Study design of AdhereApp study. Patients using edoxaban will be randomized to either an application conditioned-feedback group or a conventional therapy group.

158x77mm (96 x 96 DPI)

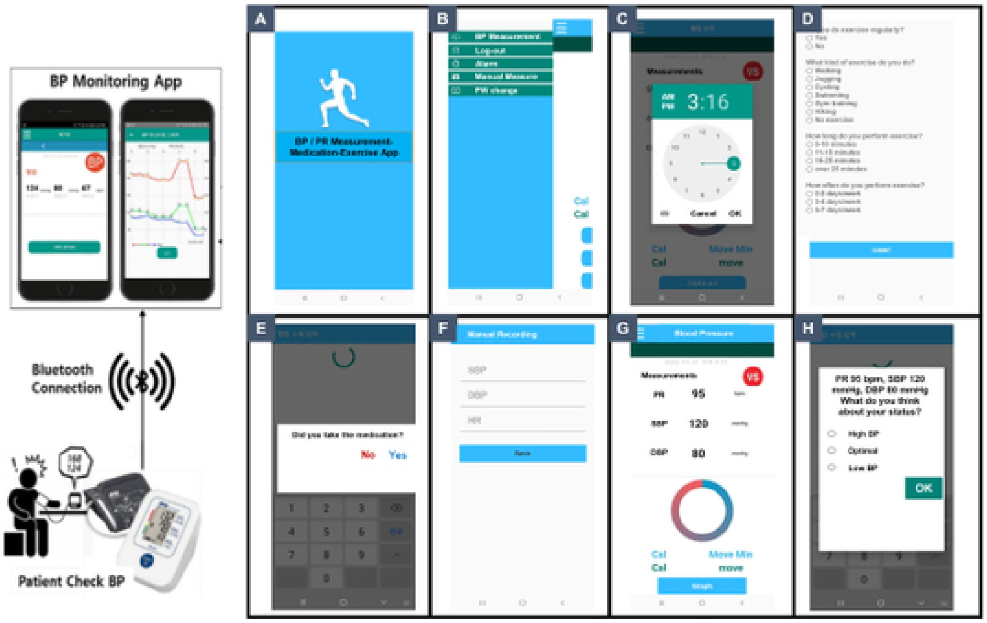

Figure. 3 Operating system and sample display of the smartphone app. When the patient measures BP, the data are automatically transferred to the app via Bluetooth system. A, title page; B, menu bar indicating BP measurement, log-out, alarm setting, manual BP input, and password setting; C, alarm setting page for medication; D, exercise information including interval, exercise type, and duration; E, alarm page to check whether the patient has taken medication; F, BP manual input page; G, page displaying measured BP and HR result; H, feed-back question to check whether the patient has achieved optimal target BP. BP, blood pressure; HR, heart rate.

46x28mm (300 x 300 DPI)

Supplementary Material

Rationale, Design, and Efficacy of a Smartphone Application for Improving Self-Awareness of Adherence to Edoxaban Treatment: A Randomized Controlled Study (Adhere App)

System Development Process

Previously, we described a five-step process to design and develop a standalone application (app) for patient blood pressure (BP) monitoring and physician's assistance. This app is currently undergoing clinical trial. In this study, we changed the last two steps of the previously described process. After a successful clinical trial (WHO ICRTP registry, ID: KCT0004754), we will evaluate the system. In this study, we developed an app with an additional functionality for monitoring the patient's physical activities. The primary functionalities of the system include patient BP and activity monitoring, medication adherence, and recommendations along with alert generation. The developed apps include a BP&Activity monitoring mobile app, electronic case report form (eCRF)-atrial fibrillation (AF), and Rule Editor. The primary users of the mobile app are registered patients, while the other two apps (eCRF-AF and Rule Editor) are mainly used by physicians to monitor the associated patients' activities and for knowledge creation, respectively. The aforementioned apps were designed and developed based on the five-step process. Supplemental Figure 1 shows the complete system design and development process.

For peer review only

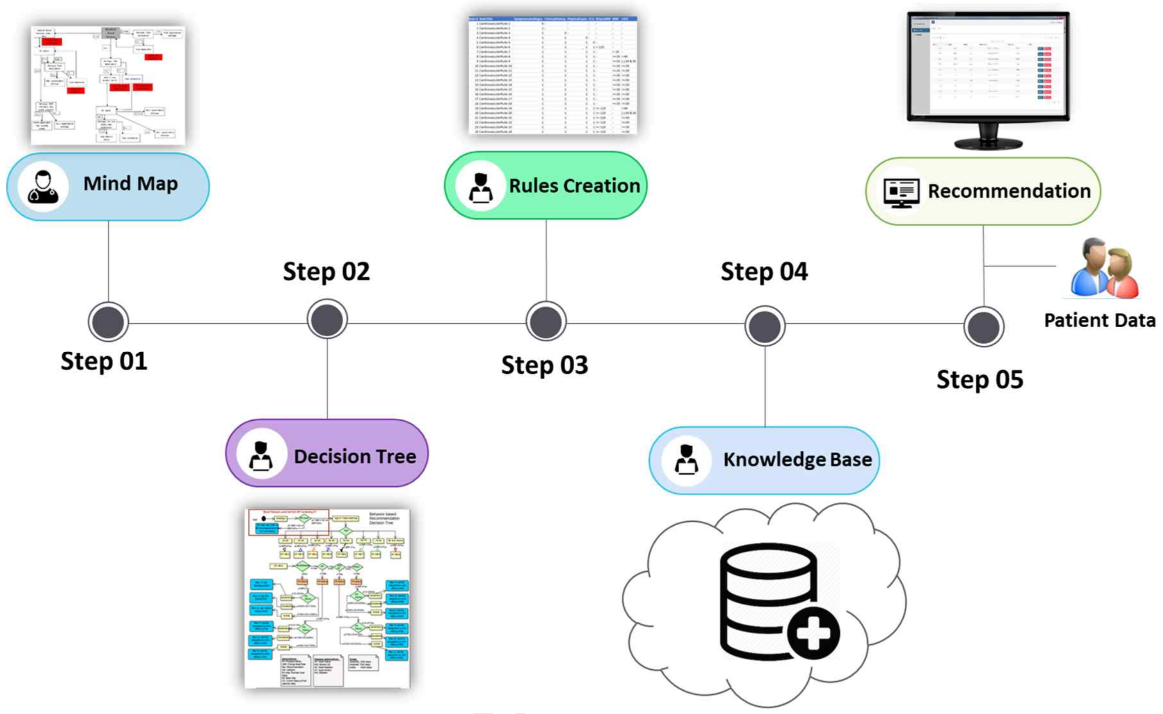

Supplemental Figure 1: System design and development process

- **Mind Map:** First, we represented the physicians' knowledge in an informal format, called the mind map, that is easily understandable to a human but not understandable to a computer. The physicians' knowledge about BP, physical activities, and their correlation was represented in the form of mind map nodes, and the nodes were related among each other based on the conditional relationships. With this analogy, the BP is linked with the age of the patient to identify the recommended activity for the patient. The resultant recommendations about the BP (systolic and diastolic), age, and current status of patient activity are connected to further diagnosis and then treatment of the patient's current condition. In short, physicians' knowledge based on their experience during real practice is represented in the mind map. We modify the initial mind map based on discussion with concerned physicians during a knowledge elicitation phase, and the final mind map is produced after a few knowledge elicitation meetings.
- **Iterative Decision Tree:** In the second phase, we transform the non-formal representation of the mind map into a more formal representation, known as the Iterative Decision Tree (IDT) [1]. The IDT representation is more understandable for physicians and computers; however,

transformation is needed for execution. In the IDT, the contributing factors are highlighted and linked with different tests to cover all the possible paths to reach diagnostic and treatment decisions. In this study, we recommend suitable physical activities to the patients based on their ages. Therefore, the patient first checks their BP, and then the system will generate a recommendation based on the recorded BP and the patient's age. Exercise goal steps (EGS) are calculated and recommended by the system to the patient to achieve the specified goal. Physicians' knowledge regarding BP is correlated with the physical activity, as shown in Supplemental Figure 2. The physicians verified and improved the IDT for all possible cases of that disease in multiple iterations of the knowledge elicitation process.

For peer review only

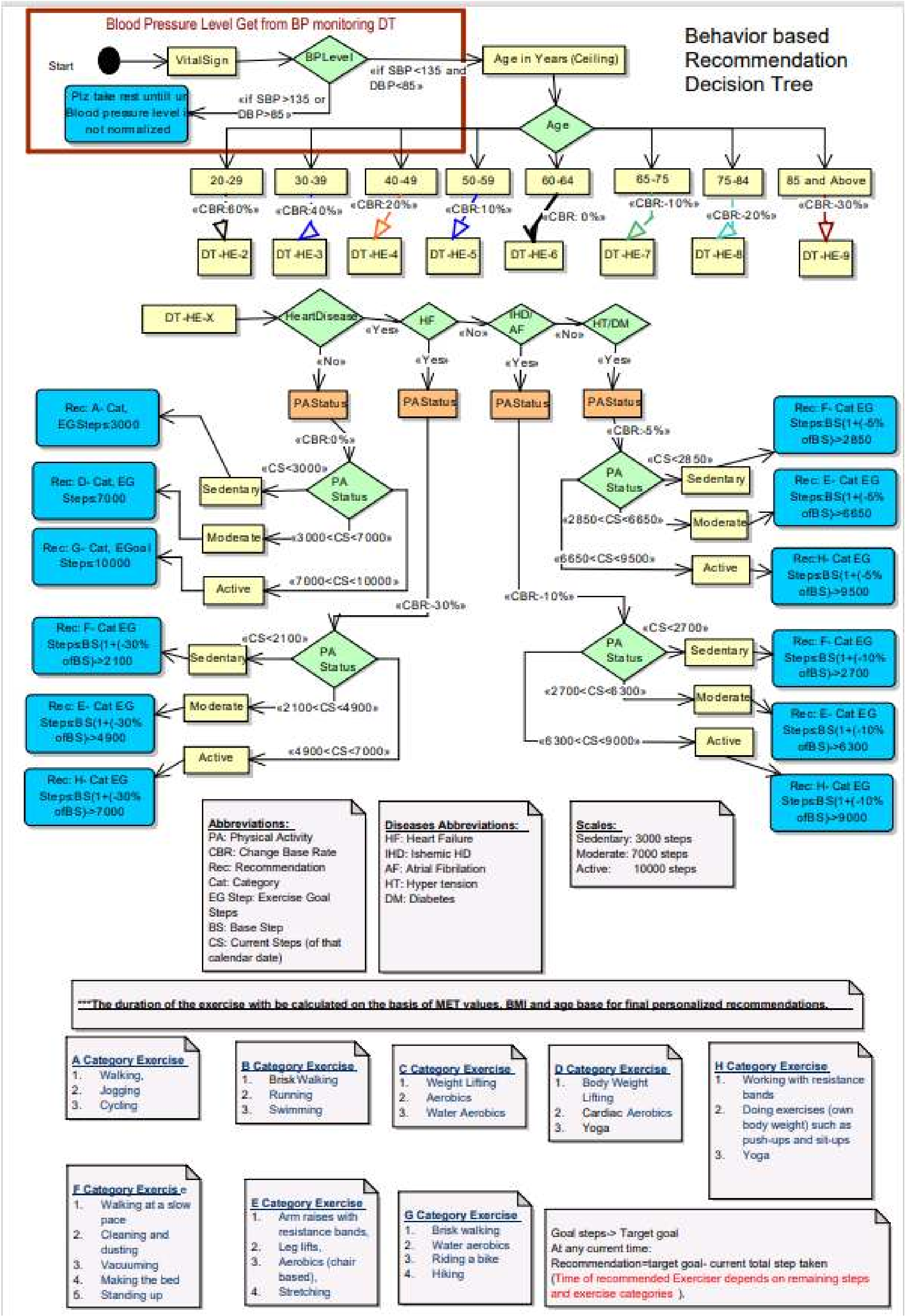

Supplemental Figure 2: Final Iterative Decision Tree (IDT) for blood pressure with physical activity

- **Rule Creation:** As previously mentioned, the IDT representation needs to be transformed into an executable format for the operative recommendations. Therefore, in the rule creation process, we transform the IDT into production rules. The production rules in object-oriented knowledge

bases are an easy way for knowledge execution. In this step, all the IDT branches are transformed into rules by top-down and left-right traversing and reasoning approaches. Our developed knowledge environment, called the Intelligent-Knowledge Authoring Tool (I-KAT), is used to transform rules into production and executable formats.

- **Knowledge Maintenance:** The knowledge maintenance phase provides a sophisticated method for validation and verification of the knowledge base. The transformed rules in the previous step are retrieved for validation and verification using a rule-based reasoning methodology. This aims to find conflict and duplication in the existing rules, if they exist. In this phase, all the rules are stored in the knowledge base in the proper hierarchy of knowledge representation.
- **Recommendation:** The recommendation phase is needed to give recommendations based on the knowledge created in the previous step. Therefore, an app is developed to provide an executable environment, which consists of a rule-based reasoner and a user-friendly interface to handle the input of signs, symptoms, history, and medical test reports to produce a final diagnosis and treatment. A mobile app was developed for BP and physical activity monitoring interventions to support patients. The app prompts the patient to monitor their BP and asks about medication intake. Based on their BP condition and medication intake status, the app recommends a suitable physical activity to perform on right time. If the condition is severe, the app recommends approaching the hospital urgently.

System Architecture and Structure

Supplemental Figure 3 shows the architecture and holistic structure of the system, which consists of four core components: (I) BP&Activity monitoring app, (II) knowledge acquisition and reasoning, (III) database, and (IV) eCRF-AF.

For peer review only

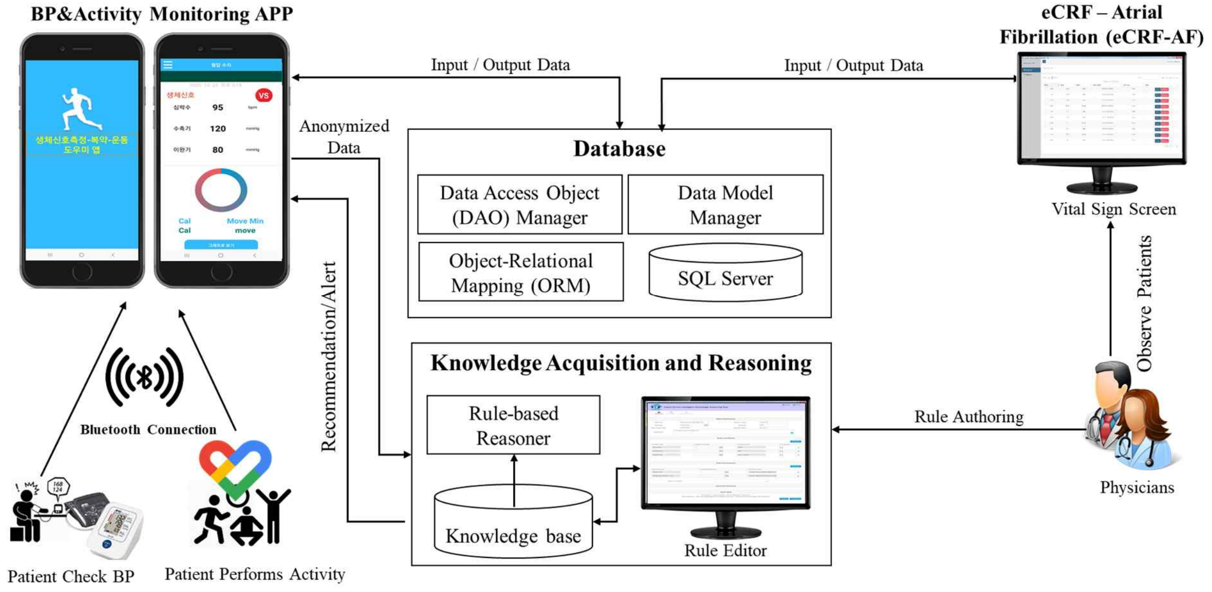

Supplemental Figure 3: System architecture and structural workflow

I. **BP&Activity Monitoring App:** This mobile app is equipped with different functionalities, such as BP and physical activity monitoring, user authentication, user registration, user exercise preferences, dashboard, BP monitoring graph, step-count graph, and recommendation screens. The core screen of the BP&Activity monitoring app is the dashboard, which facilitates users to connect with the BP monitoring device, such as the Bluetooth-enabled BP monitor UA-651BLE (A&D medical, Sydney, Australia). The dashboard screen is responsible for connecting the app with a nearby A&D PB monitoring device via Bluetooth. Similarly, the dashboard has implicit services to count the patients' steps while walking, running, jogging, and performing other physical activities. When the users check their BP, the calculated systolic BP (SBP), diastolic BP (DBP), and heart rate (HR) are sent to the mobile app. The app activates the reasoner to provide recommendations based on the calculated values. In this study, the app also considers patient age and current behavior regarding physical activity.

II. **Knowledge acquisition and reasoning:** In this component, our system provides a knowledge creation environment and reasoning capability. The I-KAT is equipped with a Rule Editor, which facilitates the creation, modification, and management of the knowledge base by physicians. The knowledge base is a core component of the clinical decision support system to

peer review only

store and maintain clinical knowledge. We are presenting an authoring environment called a Rule Editor that provides an easy-to-use interface for physicians to create clinical knowledge rules. The tool reduces the burden of memorizing and understanding the data model and vocabulary concepts for the physician because the system is equipped with intelli-sense functionality during rule creation. The reasoning capability also handles knowledge validation and verification to find duplicated and conflicted rules within the knowledge base. Finally, the rules persist in the knowledge base. When it receives a patient's recorded BP, the rule-based reasoner is activated to generate recommendations and alerts based on the recorded BP.

III. **Database:** We designed and implemented the database schema to maintain patient demographic data, vital signs (systolic BP, diastolic BP, and HR), physical activity data, and other patient-related data in the database. We implemented two types of database storage: the one for the mobile app is a lightweight database using SQL Lite, which is the default temporary storage for patient data on the smartphone, when the device is not connected to the internet. When the device is connected to the internet, the data are sent to an SQL server for long-term (permanent) storage. Three internal components, data model manager, data access object (DAO) manager, and object relational mapping (ORM) work collaboratively to perform the persistence functionality. The data model manager is responsible for managing the database schema along with data instances to store in the appropriate model. The data access object (DAO) manager is used to access the data objects of the recorded values and prepare the data model to store in the database. The object relational mapping (ORM) is used to convert data between incompatible type systems using object-oriented programming languages. The App Server/Rest API is designed and developed for storing patient information, with patient consensus, for tele-monitoring by the concerned physicians. The patient data are anonymized during communication and persistence. Therefore, only the authorized and concerned physicians can see and analyze only their assigned patients' data.

IV. **eCRF–atrial fibrillation**: Generally, the eCRF system collects patient data during clinical trials. The eCRF system usually collects patient vital sign information. SBP, DBP, and HR are

For peer review only

important vital signs that are managed within the eCRF system. We designed and developed a special purpose eCRF for patients with AF, called eCRF-AF to monitor vital signs (SBP, DBP, and HR) along with physical activities of patients. Therefore, the BP&Activity monitoring app sends the SBP, DBP, and HR and the step counts along with physical activities (sitting, walking, running, jogging, etc.) to the vital sign and physical activity repositories, respectively. These are shown to the physicians on separate analytical screens depending on the nature of the data. The eCRF-AF systems and HMIS systems of different hospitals can be integrated with the BP&Activity monitoring app. We focused and integrated the BP&Activity monitoring app with a lightweight eCRF-AF system developed for managing clinical trials. Physicians can easily observe the BP trends (daily, weekly, monthly, yearly, etc.) along with exercise, using physical activity trends of the concerned patients. If the physicians find an abnormality in the patient data, they will communicate with the respective patient.

System Implementation Outcome: Process Flow

The system comprises three main apps: one is a mobile app (BP&Activity monitoring app), which is a patient-oriented app, while the other two are web apps (eCRF-AF and Rule Editor), which are physician-oriented apps. Here, we focus on the patient-oriented BP&Activity monitoring app, which comprises 11 different screens with multiple functionalities. The BP&Activity monitoring app and its utilization and process flow are depicted in **Supplemental Figure 4**. The BP&Activity monitoring app has two processes: one process for new user registration and a second process for registered patients to check their BPs and physical activities to obtain suitable and correct recommendations based on the recorded BP, age, profile questionnaire, and exercise preferences.

User Registration Process: To register a new user on the system, we follow a three-step process to obtain user information.

- **Step 1:** The user will add profile information, such as name, gender, date of birth, and email address, and other information to register on the system.
- **Step 2:** The questionnaire screen is used to investigate physical behavior in terms of type of

For peer review only

exercise, duration of exercise, and frequency of exercise. All these questions help to calculate the patients' current behavior in terms of physical activity.

- **Step 3:** The preferences screen gives privileges to the user to select the appropriate exercises from the recommended ones to set the final recommendation as a preference.

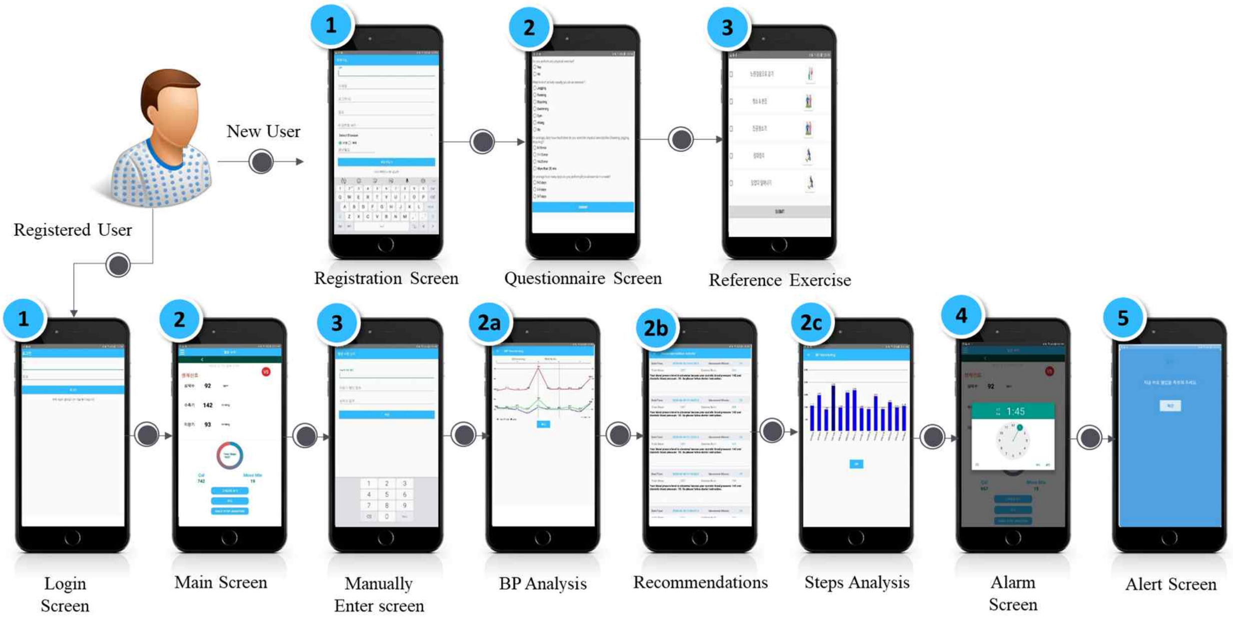

Supplemental Figure 4: Utilization and process flow of BP&Activity monitoring app (Patient-Oriented)

BP and Activity Monitoring Process: After successful registration, the patient can utilize the app to monitor their vital signs (SBP, DBP, and HR) along with their physical activity status through an A&D BP monitoring device and GoogleFit API.

- **Step 1:** The user needs to login to the system with his/her credentials for authentication.
- **Step 2:** After successful authentication, the user can access a dashboard screen. It consists of the current status of their vital signs along with the step count. It also facilitates observation of analytics and recommendation history.
- **Step 3:** The user can enter vital sign details manually when the attached device is not communicating properly. The app accepts SBP, DBP, and HR values.
- **Step 2a:** From the dashboard, the user can visualize historical analytics for vital signs, which

view only

represent the health status in terms of BP and HR.

- **Step 2b:** From the dashboard, the user can visualize historical recommendations regarding exercises based on their physical behavior with respect to time.
- **Step 2c:** From the dashboard, the user can visualize step count historical analytics, with respect to the day, to understand their own physical behavior.
- **Step 4:** The alarm screen facilitates the user to decide and set an alarm for checking their BP. At the selected time, the system will generate the alarm for the user to check their BP and take their medication, as necessary.
- **Step 5:** The system generates recommendations at different stages of BP and activity monitoring based on patient preferences, age, and answers to the questionnaire. The recommendation may issue a message to check BP, walk, run, jog, and rest for a specified time, or to take medication at the specified time.

For peer review only

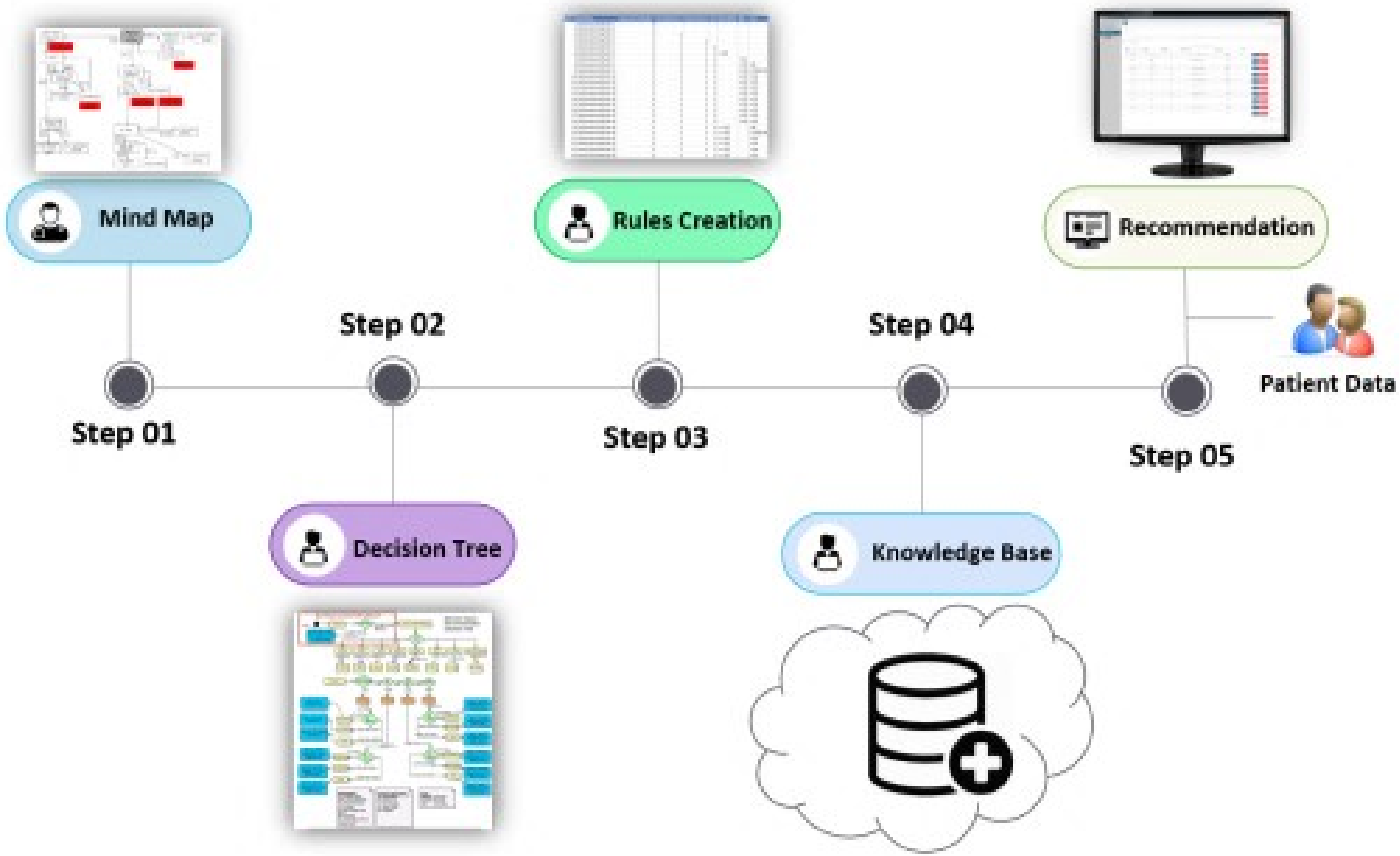
Mind Map
Rules Creation
Recommendation
Step 02
Step 04
Patient Data
Step 01
Step 03
Step 05
Decision Tree
Knowledge Base

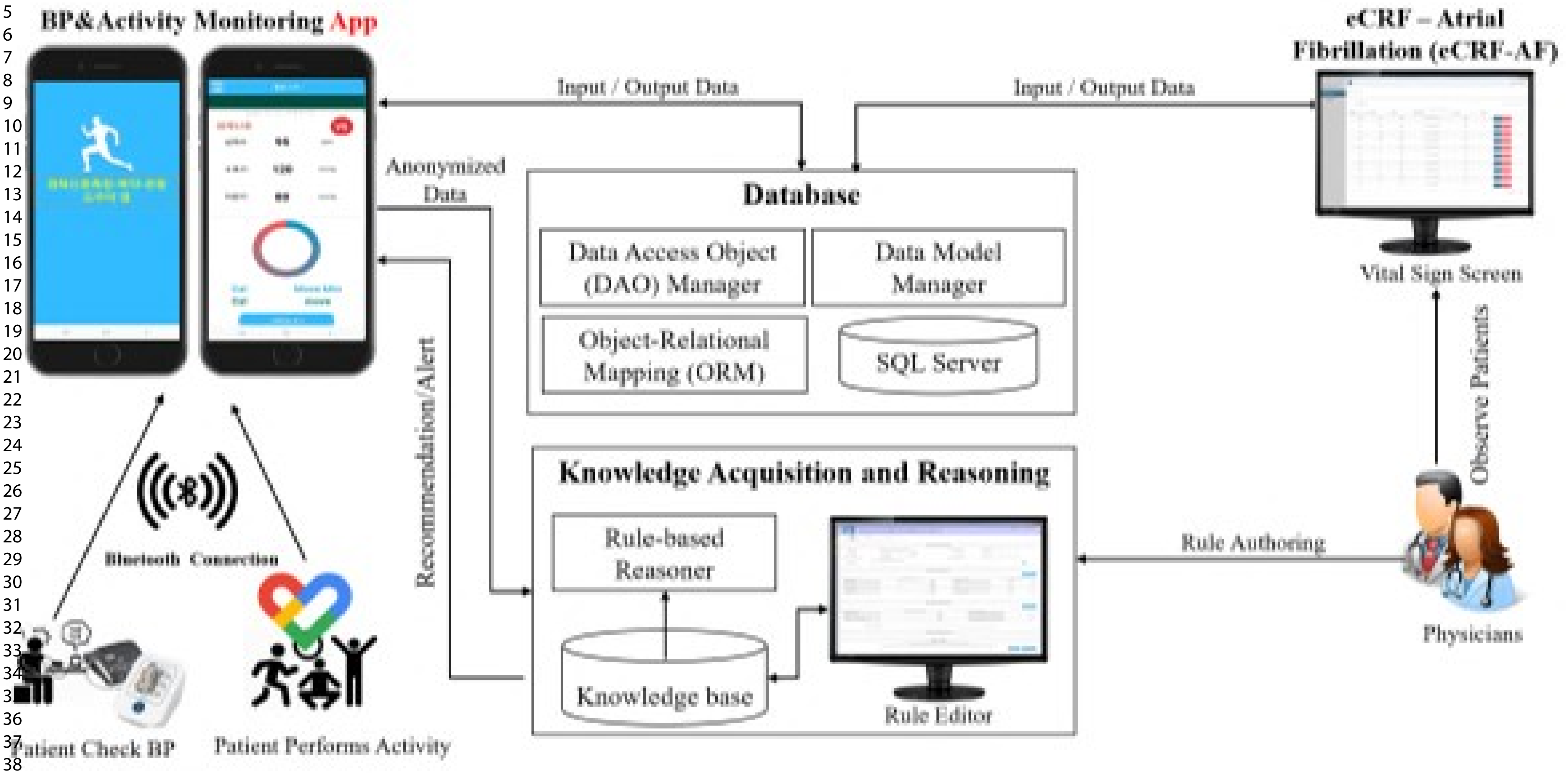
BP&Activity Monitoring App
eCRF – Atrial Fibrillation (eCRF-AF)
Input / Output Data
Input / Output Data
Anonymized Data
Database
Data Access Object (DAO) Manager
Data Model Manager
Object-Relational Mapping (ORM)
SQL Server
Vital Sign Screen
Observe Patients
Recommendation/Alert
Knowledge Acquisition and Reasoning
Rule-based Reasoner
Knowledge base
Rule Editor
Rule Authoring
Physicians
Bluetooth Connection
Patient Check BP
Patient Performs Activity

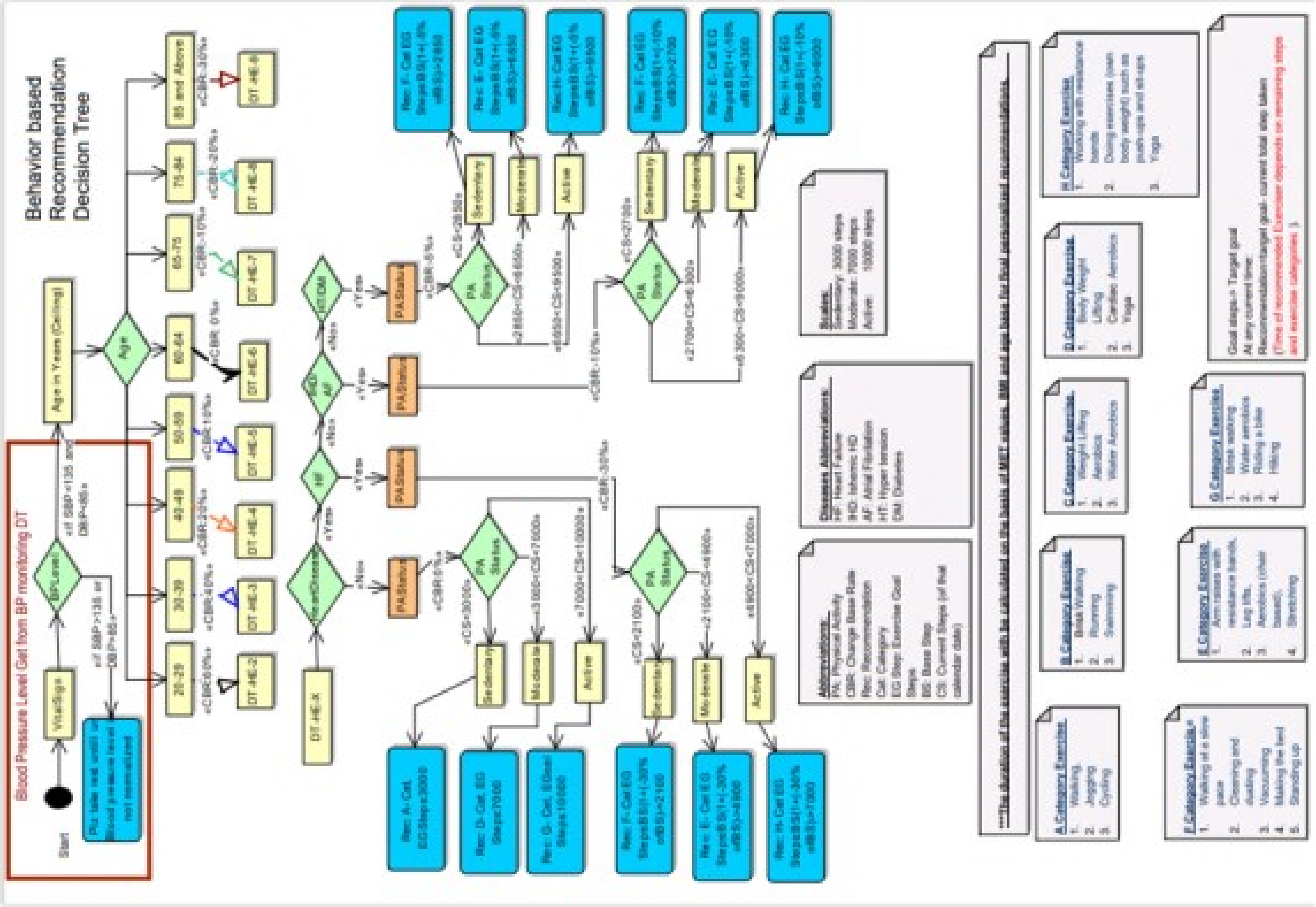
Behavior based Recommendation Decision Tree
Blood Pressure Level Get from BP monitoring DT
Start
VitalSign
BPLevel
Age in Years (Ceiling)
Age
85 and Above
75-84
65-74
60-64
50-59
40-49
30-39
20-29
DT-HE-X
PA Status
Sedentary
Moderate
Active
Abbreviations:
Disease Abbreviations:
Scale:

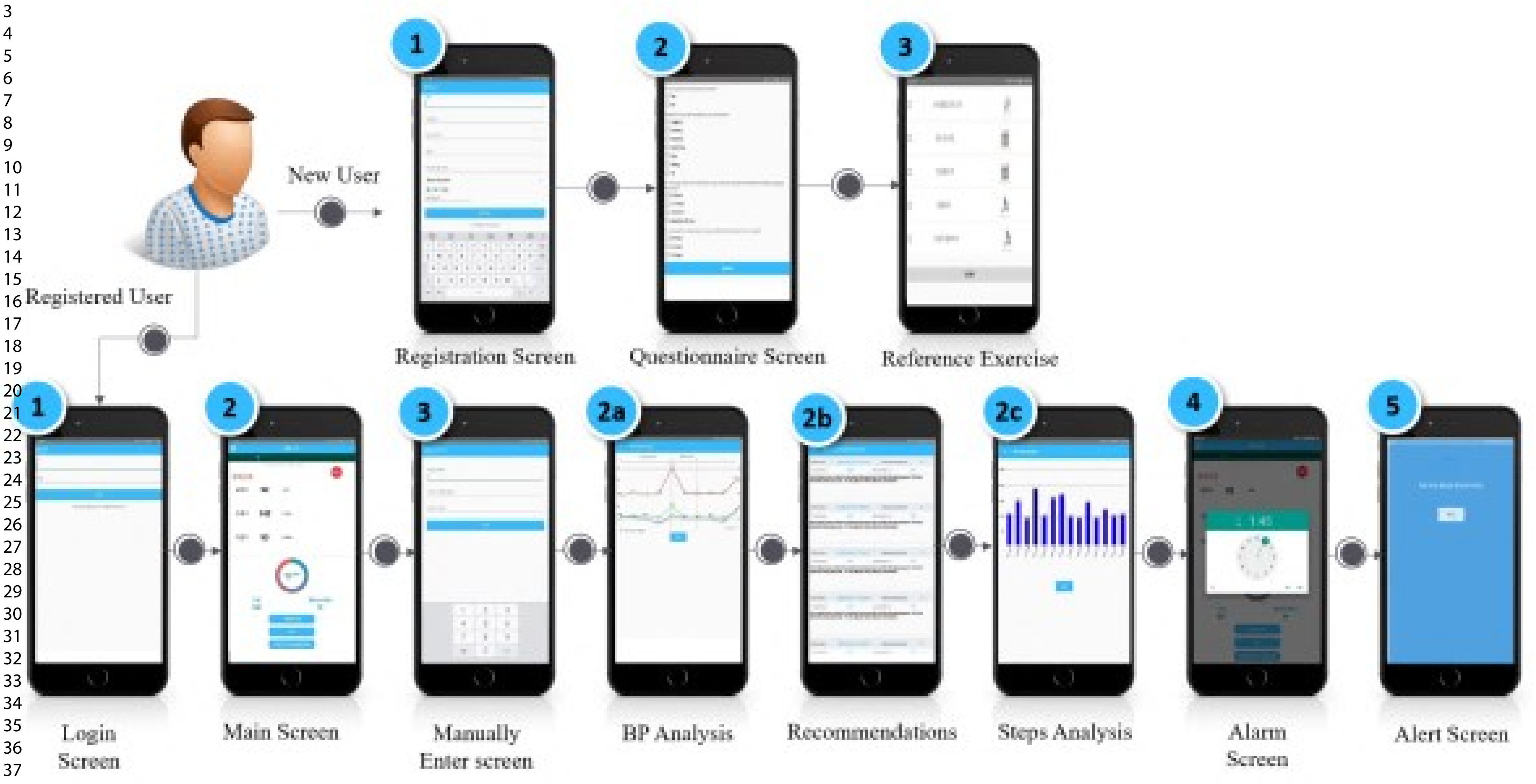
New User
Registered User
1
2
3
Registration Screen
Questionnaire Screen
Reference Exercise
1
2
3
2a
2b
2c
4
5
Login Screen
Main Screen
Manually Enter screen
BP Analysis
Recommendations
Steps Analysis
Alarm Screen
Alert Screen

Reporting checklist for protocol of a clinical trial.

Based on the SPIRIT guidelines.

Instructions to authors

Complete this checklist by entering the page numbers from your manuscript where readers will find each of the items listed below.

Your article may not currently address all the items on the checklist. Please modify your text to include the missing information. If you are certain that an item does not apply, please write "n/a" and provide a short explanation.

Upload your completed checklist as an extra file when you submit to a journal.

In your methods section, say that you used the SPIRITreporting guidelines, and cite them as:

Chan A-W, Tetzlaff JM, Gøtzsche PC, Altman DG, Mann H, Berlin J, Dickersin K, Hróbjartsson A, Schulz KF, Parulekar WR, Krleža-Jerić K, Laupacis A, Moher D. SPIRIT 2013 Explanation and Elaboration: Guidance for protocols of clinical trials. BMJ. 2013;346:e7586

		Reporting Item	Page Number
**Administrative information**			
Title	#1	Descriptive title identifying the study design, population, interventions, and, if applicable, trial acronym	1
Trial registration	#2a	Trial identifier and registry name. If not yet registered, name of intended registry	1
Trial registration: data set	#2b	All items from the World Health Organization Trial Registration Data Set	1
Protocol version	#3	Date and version identifier	8
Funding	#4	Sources and types of financial, material, and other support	16
Roles and responsibilities: contributorship	#5a	Names, affiliations, and roles of protocol contributors	15

For peer review only

Roles and responsibilities: sponsor contact information	#5b	Name and contact information for the trial sponsor	16
Roles and responsibilities: sponsor and funder	#5c	Role of study sponsor and funders, if any, in study design; collection, management, analysis, and interpretation of data; writing of the report; and the decision to submit the report for publication, including whether they will have ultimate authority over any of these activities	16
Roles and responsibilities: committees	#5d	Composition, roles, and responsibilities of the coordinating centre, steering committee, endpoint adjudication committee, data management team, and other individuals or groups overseeing the trial, if applicable (see Item 21a for data monitoring committee)	11
**Introduction**			
Background and rationale	#6a	Description of research question and justification for undertaking the trial, including summary of relevant studies (published and unpublished) examining benefits and harms for each intervention	6
Background and rationale: choice of comparators	#6b	Explanation for choice of comparators	6
Objectives	#7	Specific objectives or hypotheses	6
Trial design	#8	Description of trial design including type of trial (eg, parallel group, crossover, factorial, single group), allocation ratio, and framework (eg, superiority, equivalence, non-inferiority, exploratory)	7
**Methods: Participants, interventions, and outcomes**			
Study setting	#9	Description of study settings (eg, community clinic, academic hospital) and list of countries where data will be collected. Reference to where list of study sites can be obtained	7
Eligibility criteria	#10	Inclusion and exclusion criteria for participants. If applicable, eligibility criteria for study centres and individuals who will	8

For peer review only

		perform the interventions (eg, surgeons, psychotherapists)	
Interventions: description	#11a	Interventions for each group with sufficient detail to allow replication, including how and when they will be administered	10
Interventions: modifications	#11b	Criteria for discontinuing or modifying allocated interventions for a given trial participant (eg, drug dose change in response to harms, participant request, or improving / worsening disease)	11
Interventions: adherance	#11c	Strategies to improve adherence to intervention protocols, and any procedures for monitoring adherence (eg, drug tablet return; laboratory tests)	10
Interventions: concomitant care	#11d	Relevant concomitant care and interventions that are permitted or prohibited during the trial	10
Outcomes	#12	Primary, secondary, and other outcomes, including the specific measurement variable (eg, systolic blood pressure), analysis metric (eg, change from baseline, final value, time to event), method of aggregation (eg, median, proportion), and time point for each outcome. Explanation of the clinical relevance of chosen efficacy and harm outcomes is strongly recommended	11
Participant timeline	#13	Time schedule of enrolment, interventions (including any run-ins and washouts), assessments, and visits for participants. A schematic diagram is highly recommended (see Figure)	22
Sample size	#14	Estimated number of participants needed to achieve study objectives and how it was determined, including clinical and statistical assumptions supporting any sample size calculations	12
Recruitment	#15	Strategies for achieving adequate participant enrolment to reach target sample size	10
**Methods: Assignment of interventions (for controlled trials)**			
Allocation: sequence generation	#16a	Method of generating the allocation sequence (eg, computer-generated random numbers), and list of any factors for stratification. To reduce predictability of a random sequence, details of any planned restriction (eg, blocking) should be provided in a separate document that is unavailable to those who enrol participants or assign interventions	10

For peer review only

Allocation concealment mechanism	#16b	Mechanism of implementing the allocation sequence (eg, central telephone; sequentially numbered, opaque, sealed envelopes), describing any steps to conceal the sequence until interventions are assigned	10
Allocation: implementation	#16c	Who will generate the allocation sequence, who will enrol participants, and who will assign participants to interventions	10
Blinding (masking)	#17a	Who will be blinded after assignment to interventions (eg, trial participants, care providers, outcome assessors, data analysts), and how	10
Blinding (masking): emergency unblinding	#17b	If blinded, circumstances under which unblinding is permissible, and procedure for revealing a participant's allocated intervention during the trial	10
**Methods: Data collection, management, and analysis**			
Data collection plan	#18a	Plans for assessment and collection of outcome, baseline, and other trial data, including any related processes to promote data quality (eg, duplicate measurements, training of assessors) and a description of study instruments (eg, questionnaires, laboratory tests) along with their reliability and validity, if known. Reference to where data collection forms can be found, if not in the protocol	9
Data collection plan: retention	#18b	Plans to promote participant retention and complete follow-up, including list of any outcome data to be collected for participants who discontinue or deviate from intervention protocols	11
Data management	#19	Plans for data entry, coding, security, and storage, including any related processes to promote data quality (eg, double data entry; range checks for data values). Reference to where details of data management procedures can be found, if not in the protocol	11
Statistics: outcomes	#20a	Statistical methods for analysing primary and secondary outcomes. Reference to where other details of the statistical analysis plan can be found, if not in the protocol	11
Statistics: additional analyses	#20b	Methods for any additional analyses (eg, subgroup and adjusted analyses)	12

For peer review only

Statistics: analysis population and missing data	#20c	Definition of analysis population relating to protocol non-adherence (eg, as randomised analysis), and any statistical methods to handle missing data (eg, multiple imputation)	12
**Methods: Monitoring**			
Data monitoring: formal committee	#21a	Composition of data monitoring committee (DMC); summary of its role and reporting structure; statement of whether it is independent from the sponsor and competing interests; and reference to where further details about its charter can be found, if not in the protocol. Alternatively, an explanation of why a DMC is not needed	15
Data monitoring: interim analysis	#21b	Description of any interim analyses and stopping guidelines, including who will have access to these interim results and make the final decision to terminate the trial	15
Harms	#22	Plans for collecting, assessing, reporting, and managing solicited and spontaneously reported adverse events and other unintended effects of trial interventions or trial conduct	13
Auditing	#23	Frequency and procedures for auditing trial conduct, if any, and whether the process will be independent from investigators and the sponsor	13
**Ethics and dissemination**			
Research ethics approval	#24	Plans for seeking research ethics committee / institutional review board (REC / IRB) approval	13
Protocol amendments	#25	Plans for communicating important protocol modifications (eg, changes to eligibility criteria, outcomes, analyses) to relevant parties (eg, investigators, REC / IRBs, trial participants, trial registries, journals, regulators)	13
Consent or assent	#26a	Who will obtain informed consent or assent from potential trial participants or authorised surrogates, and how (see Item 32)	13
Consent or assent: ancillary studies	#26b	Additional consent provisions for collection and use of participant data and biological specimens in ancillary studies, if applicable	13
Confidentiality	#27	How personal information about potential and enrolled participants will be collected, shared, and maintained in order to protect confidentiality before, during, and after the trial	9

For peer review only

Declaration of interests	#28	Financial and other competing interests for principal investigators for the overall trial and each study site	16
Data access	#29	Statement of who will have access to the final trial dataset, and disclosure of contractual agreements that limit such access for investigators	13
Ancillary and post trial care	#30	Provisions, if any, for ancillary and post-trial care, and for compensation to those who suffer harm from trial participation	13
Dissemination policy: trial results	#31a	Plans for investigators and sponsor to communicate trial results to participants, healthcare professionals, the public, and other relevant groups (eg, via publication, reporting in results databases, or other data sharing arrangements), including any publication restrictions	13
Dissemination policy: authorship	#31b	Authorship eligibility guidelines and any intended use of professional writers	13
Dissemination policy: reproducible research	#31c	Plans, if any, for granting public access to the full protocol, participant-level dataset, and statistical code	13
**Appendices**			
Informed consent materials	#32	Model consent form and other related documentation given to participants and authorised surrogates	10
Biological specimens	#33	Plans for collection, laboratory evaluation, and storage of biological specimens for genetic or molecular analysis in the current trial and for future use in ancillary studies, if applicable	11

The SPIRIT Explanation and Elaboration paper is distributed under the terms of the Creative Commons Attribution License CC-BY-NC. This checklist was completed on 21. July 2021 using https://www.goodreports.org/, a tool made by the EQUATOR Network in collaboration with Penelope.ai

For peer review only

BMJ Open

Rationale, Design, and Efficacy of a Smartphone Application for Improving Self-Awareness of Adherence to Edoxaban Treatment: Study Protocol for a Randomized Controlled Trial (Adhere App)

Journal:	*BMJ Open*
Manuscript ID	bmjopen-2021-048777.R2
Article Type:	Protocol
Date Submitted by the Author:	11-Dec-2021
Complete List of Authors:	Kim, In-Cheol; Keimyung University Dongsan Hospital,<br>Lee, Ji Hyun ; Bundang Seoul National University Hospital Department of Internal Medicine, Cardiology<br>Choi, Dong-Ju; Seoul National University Bundang Hospital, Department of Internal Medicine<br>Park, Sung-Ji ; Samsung Medical Center, Division of Cardiology<br>Lee, Ju-Hee; Chungbuk National University College of Medicine, Department of Internal Medicine<br>Park, Sang Min; Chuncheon Sacred Heart Hospital<br>Kim, Mina; Korea University Anam Hospital<br>Kim, Hack-Lyoung; Seoul National University Seoul Metropolitan Government Boramae Medical Center, Internal Medicine<br>Lee, Sunki; Hallym University College of Medicine<br>Kim, In Jai; CHA University, Department of Internal Medicine<br>Choi, Seonghoon; Kangnam Sacred Heart Hospital<br>Bang, Jaehun; Kyung Hee University, Department of Computer Science and Engineering<br>Ali, Bilal; Kyung Hee University, Department of Computer Science and Engineering<br>Hussain, Musarrat; Kyung Hee University, Department of Computer Science and Engineering<br>Ali, Taqdir; Kyung Hee University, Department of Computer Science and Engineering<br>Lee, Sungyoung; Kyung Hee University, Department of Computer Science and Engineering
**Primary Subject Heading**:	Cardiovascular medicine
Secondary Subject Heading:	Medical management, Health informatics
Keywords:	MEDICAL EDUCATION & TRAINING, Cardiology < INTERNAL MEDICINE, Stroke < NEUROLOGY

Rationale, Design, and Efficacy of a Smartphone Application for Improving Self-Awareness of Adherence to Edoxaban Treatment: Study Protocol for a Randomized Controlled Trial (Adhere App)

In-Cheol Kim[1], Ji Hyun Lee[2], Dong-Ju Choi[2], Sung-Ji Park[3], Ju-Hee Lee[4], Sang Min Park[5], Mina Kim[6], Hack-Lyoung Kim[7], Sunki Lee[8], In Jai Kim[9], Seonghoon Choi[10], Jaehun Bang[11], Bilal Ali[11], Musarrat Hussain[11], Taqdir Ali[11], and Sungyoung Lee[11]

1) Division of Cardiology, Department of Internal Medicine, Keimyung University Dongsan Hospital, Keimyung University School of Medicine, Daegu, Korea

2) Division of Cardiology and Cardiovascular Center, Department of Internal Medicine, Seoul National University Bundang Hospital, Seongnam, Korea

3) Division of Cardiology, Department of Internal Medicine, Heart Vascular Stroke Institute, Samsung Medical Center, Sungkyunkwan University School of Medicine, Seoul, Korea.

4) Division of Cardiology, Department of Internal Medicine, Chungbuk National University Hospital, Chungbuk National University College of Medicine, Cheongju, Korea

5) Department of Cardiology, Chuncheon Sacred Heart Hospital, Hallym University, Chuncheon, Korea

6) Department of Cardiology, Korea University College of Medicine, Seoul, Korea

7) Division of Cardiology, Seoul National University Boramae Medical Center, Seoul, Korea

8) Division of Cardiology, Dongtan Sacred Heart Hospital, Hallym University College of Medicine, Gyunggi-do, Korea

For peer review only

9) Division of Cardiology, Department of Internal Medicine, Bundang CHA Medical Center, CHA University, Seongnam, Korea

10) Division of Cardiology, Department of Internal Medicine, Kangnam Sacred Heart Hospital, Hallym University College of Medicine, Seoul, Korea

11) Department of Computer Science and Engineering, Kyung Hee University, Yongin, Korea

* IC Kim and JH Lee contributed equally to this work

For peer review only

Word counts

Abstract: 301 words

Main text (excluding title page, abstract, references, figures, and tables): 2,530 words

Correspondence

Dong-Ju Choi, M.D. Ph.D. (https://orcid.org/0000-0003-0146-2189)

Division of Cardiology and Cardiovascular Center, Department of Internal Medicine, Seoul National University Bundang Hospital, Seongnam, Korea.

82, gumi-ro 173 Beon-gil, Bundang-gu, Seongnam, Gyeonggido, 13620, Korea

Tel: 82-31-787-7007, Fax: 82-31-787-4051

E-mail: djchoi@snubh.org

ABSTRACT

Introduction: High adherence to oral anticoagulants is essential for stroke prevention in patients with atrial fibrillation (AF). We developed a smartphone application (app) that pushes alarms for taking medication and measuring blood pressure (BP) and heart rate (HR) at certain times of the day. In addition to drug alarms, the habit of measuring one's BP and HR may reinforce drug adherence by improving self-awareness of the disease. This pilot study aims to test the feasibility and efficacy of the smartphone app-based intervention for improving drug adherence in patients with AF.

Methods and analysis: A total of 10 university hospitals in Korea will participate in this randomized control trial. Patients with AF, being treated with edoxaban for stroke prevention will be included in this study. Total of 500 patients will be included and the patients will be randomized to the conventional treatment group (250 patients) and the app conditional feedback group (250 patients). Patients in the app conditional feedback group will use the medication reminder app for medication and BP check alarms. The automatic BP machine will be linked to the smartphone via Bluetooth. The measured BP and HR will be updated automatically on the smartphone app. The primary endpoint is edoxaban adherence by pill count measurement at three and six months of follow-up. Secondary endpoints are clinical composite endpoints including stroke, systemic embolic event, major bleeding requiring hospitalization or transfusion, or death during the six-months. As of 24th of November 2021, 80 patients were enrolled.

Ethics and dissemination: This study was approved by the Seoul National University Bundang Hospital Institutional Review Board and will be conducted according to the principles of the Declaration of Helsinki. The study results will be published in a reputable journal.

Trial registration: WHO ICRTP registry, ID: KCT0004754. As of 24th of November 2021, 80 patients were enrolled.

Key words

For peer review only

atrial fibrillation; drug adherence; smartphone application; edoxaban

For peer review only

Strengths and limitations of this study.

- This randomized control trial investigates the feasibility and efficacy of a smartphone app-based intervention for improving adherence to edoxaban in multiple centers.
- The patient gets alarm to take medication and measure blood pressure with heart rate using an automated electronic manometer according to the pre-specified schedule on smartphone app.
- The physician can obtain the information regarding the blood pressure and heart rate data and drug adherence by checking the app on the follow-up visit.
- The sample size was calculated to show the difference in drug adherence, and not for any clinical events.
- The study results may not be applicable to patients who are not capable of using smartphones.

For peer review only

INTRODUCTION

Stroke prevention with oral anticoagulants is crucial in the management of atrial fibrillation (AF).[1] the Large randomized control trials with AF patients have consistently shown comparable effectiveness and improved safety of non-vitamin K oral anti-coagulants (NOAC) compared with warfarin.[2-6] Based on these trials, the current global guidelines recommend NOACs over warfarin for stroke prevention in patients with AF.[1 7 8]

In contrast to warfarin, NOACs have predictable effectiveness and minimal drug interactions. Consequently, regular blood tests for the monitoring of anticoagulation effects are not necessary with NOAC therapy. The use of NOACs is increasing worldwide because of these advantages.[9 10] However, there are some concerns regarding the relatively short half-life on NOACs. When doses are missed, a prothrombotic status might adversely triggered.[11] Thus, in NOAC therapy, consistent drug adherence is essential to maintain a consistent anticoagulation effect for stroke prevention. Poor drug adherence or failed persistence would increase the risk of stroke, and this may lead to an increase in public health care costs.[12]

Even though there are controversies regarding their efficacy, smartphone applications (apps) are currently being recognized as an effective tool for promoting drug adherence[13-23]. Smartphone apps send notifications or push alarms reminding the patient to take their medication. Moreover, active involvement in measuring their health status – blood pressure (BP) and heart rate (HR) using smartphone app-based feedback system could improve awareness of their underlying condition. This in turn influences their self-care behavior and increases drug adherence[24]. The aim of this study protocol is to test whether smartphone app-based intervention that provides push alarms for taking medication and measuring BP and HR, would increase the drug adherence compared to usual care in AF patients requiring oral anticoagulation therapy.

For peer review only

METHODS AND ANALYSIS

Study design

The Adhere-App (Self-awareness of Drug Adherence to Edoxaban Using an Automatic App. Feedback System) study is a multicenter, randomized, open-label, and group-comparison trial to assess the effect of using a smartphone app to improve drug adherence (**Figure 1**). A total of 10 tertiary university hospitals in Korea will participate in this study. The study participants will be randomized to a smartphone app-conditioned feedback group or a conventional treatment group. Every patient will receive education on AF and the importance of anticoagulation therapy. Additionally, the intervention group will be educated on the use of the smartphone app coupled with BP and HR measurement using an automatic BP measuring machine. Study visits will be performed at baseline, three months, and six months (**Figure 2**). When clinical visits are not possible due to the inevitable situation, the patient's condition and survey data can be collected by telephone visit. This study was registered in the International Clinical Trials Registry Platform (ICTRP) on 20 February 2020. However, initiation was delayed due to coronavirus disease 2019 (COVID-19) pandemic. As of 24th of November 2021, 80 patients were enrolled.

Study participants

Adult patients (≥ 19 years old) with non-valvular AF requiring oral anticoagulation therapy (CHA_2DS_2-VASC score ≥ 2 points) for stroke prevention will be enrolled. Edoxaban (Lixiana®, Daiichi Sankyo, Tokyo, Japan) will be administered to patients at an on-label dosage of 60 mg once daily. The dosage will be reduced to 30 mg once daily in patients who meet any of the following criteria: moderate renal impairment (creatinine clearance 30-50 mL/min), body weight less than or equal to 60 kg, or concomitant use of potent P-glycoprotein inhibitors.

Since smartphone usage is crucial in this study, participants should be able to use smartphone well, and be capable of following instructions on how to use the app in Korean language. Patients with severe

For peer review only

renal insufficiency (creatinine clearance < 15 mL/min) and significant mitral valve disease will be excluded. The detailed inclusion and exclusion criteria for the Adhere-App study are listed in **Table 1**.

Table 1. Key inclusion and exclusion criteria for Adhere-App study.

Inclusion criteria<br>• Patients with atrial fibrillation, treated with edoxaban for stroke prevention<br>• Age ≥ 19 years<br>• Capable of using smartphone application<br>• Competency in Korean language<br>• Accepted for the study protocol and willing to participate in the clinical study
Exclusion criteria<br>• Creatinine clearance less than 15 mL/min<br>• Patients with dual antiplatelet therapy<br>• Moderate or severe mitral stenosis<br>• Previous history of mitral valve replacement or mitral valve repair<br>• Previous history of alcohol or drug abuse<br>• Not suitable for the clinical trial enrollment by the judgement of the investigator<br>• History of previous enrollment in another clinical trial using an investigational pharmaceutical product<br>• Patient unwilling to participate in the clinical study

For peer review only

At baseline, age, sex, details of comorbidities, and laboratory findings will be assessed for all patients. The CHA_2DS_2-VASc score for stroke risk prediction is calculated by the summation of all assigned points for each particular medical condition: one point each for age between 65 and 74 years (A), female sex (Sc), hypertension (H), diabetes mellitus (D), congestive heart failure (C), and vascular disease (V, prior myocardial infarction or peripheral artery disease) and 2 points each for a history of stroke/transient ischemic attack/thromboembolism (S_2) or age of ≥ 75 years (A_2).[25]

Patient and public involvement

Patients or the public will not be involved in the design, reporting, or dissemination of our research.

Patient recruitment and randomization

Potential participants will be any patients visiting the clinic for AF management. After a comprehensive interview, written informed consent will be obtained from all eligible participants. Eligible participants will be asked to complete a baseline survey covering demographics and cardiovascular comorbidities. The eligible participants will then be randomly assigned to either the intervention group or the conventional treatment group in a 1:1 ratio using a web-based central randomization service (http://matrixmdr.com/). Allocation concealment would be ensured, as the service would not release the randomisation results until the patient has been enrolled into the trial. Due to the nature of the intervention, it is not possible to blind participants to group allocation. All participants will be informed that the information on edoxaban adherence will be collected for analysis.

Intervention (Smartphone app-conditioned feedback)

For peer review only

Study participants in the intervention group will install the study app provided by clinical research coordinators on their own smartphone. Since the app is not an open application to the public, the control group can't access to application. The operating system and sample display of the smartphone app are summarized in **Figure 3**. The participants can set the alarm for taking edoxaban at a certain time of the day. The research coordinator or the physician will set the alarm initially. Patient's exercise information, including interval, exercise type, and duration, will be submitted on the first visit. When the alarm sounds at the pre-specified time, the patient will be asked to take the drug (edoxaban) and measure their BP and HR using an automated electronic manometer (UA-651BLE Bluetooth Blood Pressure monitor, A&D Medical, Sydney, Australia). The measured BP and HR data will be automatically transmitted to the smartphone via Bluetooth, as shown in **Supplemental Figure 1, 2, 3 and 4**. To evaluate awareness of disease status, the app will ask whether the measured BP result is optimal, and the patient is required to respond to the question. The app will also check whether the patient took the medication. If the patient does not affirm taking the medication, the app will send an alert message to ensure that the patient takes the medication. The data regarding the time of edoxaban intake, BP, and HR will be automatically updated and stored daily. Finally, the physician can obtain feedback on BP and HR measurement data and drug adherence by checking the app on the follow-up visit. The aforementioned functionalities will be provided by the smartphone app, while a standalone web application has been developed for physicians to monitor associated patients' BP, physical activity, and medication adherence trends. The design, development, and utilization detail of the patient-oriented smartphone app and physician-oriented web app are described in the **Supplemental Materials**.

Control (Conventional treatment)

In the control group, only usual guideline-recommended treatments, including education on the disease and the importance of taking medications by physicians at every routine visit will be covered. No other specific interventions will be made. Patients will be followed up in clinics according to the study protocol (three months and six months) and be provided usual care for AF.

For peer review only

Study endpoints and follow up

The primary endpoint is edoxaban adherence by pill count measurement at second (three months) and third (six months) visits. All patients will be asked to bring their remaining pills at each clinical visit. Secondary endpoints are clinical composite endpoints, including stroke, systemic embolic event, major bleeding requiring hospitalization or transfusion, or death during the six months of follow up by electronic medical records. The primary and secondary endpoints will be compared between smartphone app-conditioned feedback group and control group. An additional pre-defined observational parameter is symptom change related to BP (both systolic and diastolic) and HR. Clinical research coordinators will ask symptoms as an open question and each investigator will determine whether the symptom is related with BP and HR change. The study participants would be followed up relatively short period (6 months) and the protocol of current research carries only minimal risk. Thus, data monitoring committee and interim analysis plan would not be established.

Sample size calculation

This is a pilot study to assess the efficacy of a smartphone-based intervention. To date, there has been no study evaluating the use of a smartphone app to improve NOAC adherence in Korea. Thus, a precise sample size calculation was not available. We assumed that drug adherences would be 90% in the control group and 95% in the intervention group according to the previous study[26]. A sample size of 234 patients in each group achieves 95% power to detect a mean difference of 5.0 with a known standard deviation of differences of 15.0 and a significance level (alpha) of 0.05, using a two-sided paired z-test. Considering dropout rate of 4%, we finally planned to enroll 500 patients (250 patients in each group). We expect this pilot study will provide valuable information regarding the appropriate sample size, recruitment rate, study period and data management process etc., for assessing the feasibility of the full-

For peer review only

scale study we are planning further. And it also would allow to identify potential practical problems of whole research process.

Statistical analysis

Categorical variables will be presented as numbers and frequencies, whereas continuous variables will be presented as means ± standard deviations. The student's t-test and Kruskal-Wallis test will be used to compare continuous variables depending on the presence of normally distributed variables. The chi-square test will be used to compare categorical variables. A p-value < 0.05 was considered significant. The analysis would be performed according to the intention-to-treat principle. The standard regimen of edoxaban is once daily. The primary endpoint of edoxaban adherence will be calculated by pill count as shown below.

$$\text{Pill count adherence (\%)} = \frac{\text{Number of pills dispensed} - \text{Number of pills remaining}}{\text{Number of days between dispensing date and follow up date}} \times 100$$

Since new drug users might have a lower adherence than continuous users, subgroup analysis will also be performed on the new and continuous users. To evaluate the feasibility and efficacy of the smart phone application, further analysis can be performed according to the frequency of the smart phone application use. The secondary endpoints of adverse events will be compared with the Kaplan-Meier curve analysis. A log rank test will be used to evaluate the significance of the difference between the two groups. Statistical analysis will be performed using IBM SPSS Statistics for Windows (IBM Corp., Armonk, N.Y., USA) and R software version 4.0.3.

Ethics and dissemination.

This study was approved by the institutional review board of Seoul National University Bundang Hospital and will be conducted according to the principles of the Declaration of Helsinki. The potential risks to participants are negligible in the present study. The participants will be handed study information sheets and asked to provide written informed consent. The study results will be

For peer review only

disseminated via publication in a reputable journal and presentation at scientific meetings. Individual participant data that underlie the results reported (text, tables, figures and appendices) will be shared after deidentification process upon reasonable request.

DISCUSSION

This randomized control trial investigates the feasibility and efficacy of using a smartphone app to improve adherence to anticoagulation therapy (edoxaban). Furthermore, this study will also assess the influence of app-based interventions influences on the reduction of AF-related adverse clinical events. Today, smartphones have became an integral part of everyday life. We believe that our study will be helpful when formulating public health strategies for improving NOAC adherence using smartphones.

All participants in the intervention group will be asked to input the measured BP and HR data on a smartphone, in addition to responding to a push alarm for taking their medication. The recognition of the irregular rhythm of AF during heart rate measurement could remind the participants of the necessity of anticoagulation therapy, which they will be informed about by their physician during the education session. Feedback questions to remind the patient of their BP control status might further reinforce disease awareness and drug adherence. The stored information could also be used as a reference to set an optimal therapeutic goal for education and medication control.

A similar study was performed with NOACs, using a telemonitoring system accompanied by personal telephone feedback.[12] In that study, although the telemonitoring system achieved high drug adherence (over 90%), it was not cost-effective. The human resource and system set-up costs to operate telemonitoring-based feedback impeded the wide usage of this method in clinical practice. Digital interventions using smartphone apps are becoming an increasingly common way to support medication adherence and self-management in chronic disorders.[27] Smartphones also help to improve access to cardiac rehabilitation and heart failure management for patients unable to attend traditional center-based cardiac rehabilitation[28] We believe that our strategy of using a smartphone app would be an effective

For peer review only

way to increase drug adherence at a minimum cost.

As with other current management strategies, apps can evolve into tailored programs. Besides the simple reminders, this strategy can be incorporated into a multimodal strategy that results in sustained improvements in adherence.[29] Reminders primarily focus on unintentional nonadherence, identifying the reasons for nonadherence and developing a scale that assesses unintentional nonadherence.[30-32] This randomized trial will not only evaluate the effectiveness of the reminder app on disease awareness and drug adherence in patients with AF but will also try to find components that may hinder patient adherence to NOACs. This information could be used to develop a strategy to ensure sustained improvement in adherence to current medical therapy.

Strengths and limitations

Several limitations of this study should be addressed. The study participants do not represent the general population with AF. All participants should be able to use smartphone apps without difficulties. Thus, the results of the study may not be applicable to patients who are not capable of using smartphones, such as the extreme elderly. Second, the study participants who agree to participate in the study might have a higher interest in their own health. Consequently, higher adherence to drug therapy might be observed than that of the general AF population. Third, the sample size was calculated to show the difference in drug adherence. It is not powered to show any differences in clinical events. Despite these limitations, a major strength is that the present study is a multicenter randomized control trial with a relatively large number of participants.

Author Contributions

DJC, ICK, and JHL contributed to the conception of the study. DJC, ICK, JHL, and MK participated in the design of the study. ICK and JHL drafted and finalized the manuscript. SJP, JHL, SMP, MK, HLK,

For peer review only

SL, IJK, SC, JB, BA, MH, TA, SL and DJC revised the manuscript critically for important intellectual content.

Funding statement

This work was supported by a grant from Daiichi Sankyo, Korea (06-2019-233). The funders have no influence on the study design, data collection or the decision to submit reports for publication.

Competing interests statement.

None declared.

For peer review only

References

1. Hindricks G, Potpara T, Dagres N, et al. 2020 ESC Guidelines for the diagnosis and management of atrial fibrillation developed in collaboration with the European Association of Cardio-Thoracic Surgery (EACTS). *Eur Heart J* 2020 doi: 10.1093/eurheartj/ehaa612

2. Ruff CT, Giugliano RP, Braunwald E, et al. Comparison of the efficacy and safety of new oral anticoagulants with warfarin in patients with atrial fibrillation: a meta-analysis of randomised trials. *Lancet* 2014;383(9921):955-62. doi: 10.1016/S0140-6736(13)62343-0

3. Connolly SJ, Ezekowitz MD, Yusuf S, et al. Dabigatran versus warfarin in patients with atrial fibrillation. *N Engl J Med* 2009;361(12):1139-51. doi: 10.1056/NEJMoa0905561

4. Patel MR, Mahaffey KW, Garg J, et al. Rivaroxaban versus warfarin in nonvalvular atrial fibrillation. *N Engl J Med* 2011;365(10):883-91. doi: 10.1056/NEJMoa1009638

5. Granger CB, Alexander JH, McMurray JJ, et al. Apixaban versus warfarin in patients with atrial fibrillation. *N Engl J Med* 2011;365(11):981-92. doi: 10.1056/NEJMoa1107039

6. Giugliano RP, Ruff CT, Braunwald E, et al. Edoxaban versus warfarin in patients with atrial fibrillation. *N Engl J Med* 2013;369(22):2093-104. doi: 10.1056/NEJMoa1310907

7. January CT, Wann LS, Calkins H, et al. 2019 AHA/ACC/HRS Focused Update of the 2014 AHA/ACC/HRS Guideline for the Management of Patients With Atrial Fibrillation: A Report of the American College of Cardiology/American Heart Association Task Force on Clinical Practice Guidelines and the Heart Rhythm Society in Collaboration With the Society of Thoracic Surgeons. *Circulation* 2019;140(2):e125-e51. doi: 10.1161/CIR.0000000000000665 [published Online First: 2019/01/29]

8. Kirchhof P, Benussi S, Kotecha D, et al. 2016 ESC Guidelines for the management of atrial fibrillation developed in collaboration with EACTS. *Eur Heart J* 2016;37(38):2893-962. doi: 10.1093/eurheartj/ehw210 [published Online First: 2016/08/28]

9. Lee SR, Choi EK, Han KD, et al. Temporal trends of antithrombotic therapy for stroke prevention in Korean patients with non-valvular atrial fibrillation in the era of non-vitamin K antagonist oral anticoagulants: A nationwide population-based study. *PLoS One* 2017;12(12):e0189495. doi:

For peer review only

10.1371/journal.pone.0189495 [published Online First: 2017/12/21]

10. Huisman MV, Rothman KJ, Paquette M, et al. The Changing Landscape for Stroke Prevention in AF: Findings From the GLORIA-AF Registry Phase 2. *J Am Coll Cardiol* 2017;69(7):777-85. doi: 10.1016/j.jacc.2016.11.061

11. How CH. Novel oral anticoagulants for atrial fibrillation. *Singapore Med J* 2015;56(12):657-8; quiz 59. doi: 10.11622/smedj.2015184

12. Desteghe L, Vijgen J, Koopman P, et al. Telemonitoring-based feedback improves adherence to non-vitamin K antagonist oral anticoagulants intake in patients with atrial fibrillation. *Eur Heart J* 2018;39(16):1394-403. doi: 10.1093/eurheartj/ehx762 [published Online First: 2018/01/05]

13. Weisman O, Schonherz Y, Harel T, et al. Testing the Efficacy of a Smartphone Application in Improving Medication Adherence, Among Children with ADHD. *Isr J Psychiatry Relat Sci* 2018;55(2):59-63.

14. Morawski K, Ghazinouri R, Krumme A, et al. Association of a Smartphone Application With Medication Adherence and Blood Pressure Control: The MedISAFE-BP Randomized Clinical Trial. *JAMA Intern Med* 2018;178(6):802-09. doi: 10.1001/jamainternmed.2018.0447

15. Huang Z, Tan E, Lum E, et al. A Smartphone App to Improve Medication Adherence in Patients With Type 2 Diabetes in Asia: Feasibility Randomized Controlled Trial. *JMIR Mhealth Uhealth* 2019;7(9):e14914. doi: 10.2196/14914

16. Sarzynski E, Decker B, Thul A, et al. Beta Testing a Novel Smartphone Application to Improve Medication Adherence. *Telemed J E Health* 2017;23(4):339-48. doi: 10.1089/tmj.2016.0100

17. Yoon CH, Ritchie SR, Duffy EJ, et al. Impact of a smartphone app on prescriber adherence to antibiotic guidelines in adult patients with community acquired pneumonia or urinary tract infections. *PLoS One* 2019;14(1):e0211157. doi: 10.1371/journal.pone.0211157

18. Wu YP, Linder LA, Kanokvimankul P, et al. Use of a Smartphone Application for Prompting Oral Medication Adherence Among Adolescents and Young Adults With Cancer. *Oncol Nurs Forum* 2018;45(1):69-76. doi: 10.1188/18.ONF.69-76

For peer review only

19. Yu C, Liu C, Du J, et al. Smartphone-based application to improve medication adherence in patients after surgical coronary revascularization. *Am Heart J* 2020;228:17-26. doi: 10.1016/j.ahj.2020.06.019

20. Himelhoch S, Kreyenbuhl J, Palmer-Bacon J, et al. Pilot feasibility study of Heart2HAART: a smartphone application to assist with adherence among substance users living with HIV. *AIDS Care* 2017;29(7):898-904. doi: 10.1080/09540121.2016.1259454

21. Talmor G, Nguyen B, Keibel A, et al. Use of software applications to improve medication adherence and achieve more integrated disease management in heart failure. *Trends Cardiovasc Med* 2018;28(7):483-88. doi: 10.1016/j.tcm.2018.04.001

22. Johnston N, Bodegard J, Jerstrom S, et al. Effects of interactive patient smartphone support app on drug adherence and lifestyle changes in myocardial infarction patients: A randomized study. *Am Heart J* 2016;178:85-94. doi: 10.1016/j.ahj.2016.05.005

23. Marquez Contreras E, Marquez Rivero S, Rodriguez Garcia E, et al. Specific hypertension smartphone application to improve medication adherence in hypertension: a cluster-randomized trial. *Curr Med Res Opin* 2019;35(1):167-73. doi: 10.1080/03007995.2018.1549026

24. Kelders SM, Kok RN, Ossebaard HC, et al. Persuasive system design does matter: a systematic review of adherence to web-based interventions. *J Med Internet Res* 2012;14(6):e152. doi: 10.2196/jmir.2104 [published Online First: 2012/11/16]

25. Lip GY, Nieuwlaat R, Pisters R, et al. Refining clinical risk stratification for predicting stroke and thromboembolism in atrial fibrillation using a novel risk factor-based approach: the euro heart survey on atrial fibrillation. *Chest* 2010;137(2):263-72. doi: 10.1378/chest.09-1584

26. Hwang J, Han S, Bae HJ, et al. NOAC Adherence of Patients with Atrial Fibrillation in the Real World: Dosing Frequency Matters? *Thromb Haemost* 2020;120(2):306-13. doi: 10.1055/s-0039-1697954 [published Online First: 20191028]

27. Morrissey EC, Casey M, Glynn LG, et al. Smartphone apps for improving medication adherence in hypertension: patients' perspectives. *Patient Prefer Adherence* 2018;12:813-22. doi:

For peer review only

10.2147/PPA.S145647 [published Online First: 2018/05/23]

28. Hamilton SJ, Mills B, Birch EM, et al. Smartphones in the secondary prevention of cardiovascular disease: a systematic review. *BMC Cardiovasc Disord* 2018;18(1):25. doi: 10.1186/s12872-018-0764-x [published Online First: 2018/02/09]

29. Haynes RB, Ackloo E, Sahota N, et al. Interventions for enhancing medication adherence. *Cochrane Database Syst Rev* 2008(2):CD000011. doi: 10.1002/14651858.CD000011.pub3

30. Gadkari AS, McHorney CA. Unintentional non-adherence to chronic prescription medications: how unintentional is it really? *BMC Health Serv Res* 2012;12:98. doi: 10.1186/1472-6963-12-98

31. Dayer L, Heldenbrand S, Anderson P, et al. Smartphone medication adherence apps: potential benefits to patients and providers: response to Aungst. *J Am Pharm Assoc (2003)* 2013;53(4):345. doi: 10.1331/JAPhA.2013.13121

32. Dayer L, Heldenbrand S, Anderson P, et al. Smartphone medication adherence apps: potential benefits to patients and providers. *J Am Pharm Assoc (2003)* 2013;53(2):172-81. doi: 10.1331/JAPhA.2013.12202

For peer review only

Figure legends

Fig. 1 Schematic diagram of expected improvement in NOAC adherence by regular measurement of BP, heart rate and smartphone feedback in patients with atrial fibrillation. NOAC, non-vitamin K oral anti-coagulant; BP, blood pressure; HR, heart rate.

Fig. 2 Study design of AdhereApp study. Patients using edoxaban will be randomized to either an application conditioned-feedback group or a conventional therapy group.

Fig. 3 Operating system and sample display of the smartphone app. When the patient measures BP, the data are automatically transferred to the app via Bluetooth system. A, title page; B, menu bar indicating BP measurement, log-out, alarm setting, manual BP input, and password setting; C, alarm setting page for medication; D, exercise information including interval, exercise type, and duration; E, alarm page to check whether the patient has taken medication; F, BP manual input page; G, page displaying measured BP and HR result; H, feed-back question to check whether the patient has achieved optimal target BP. BP, blood pressure; HR, heart rate.

For peer review only

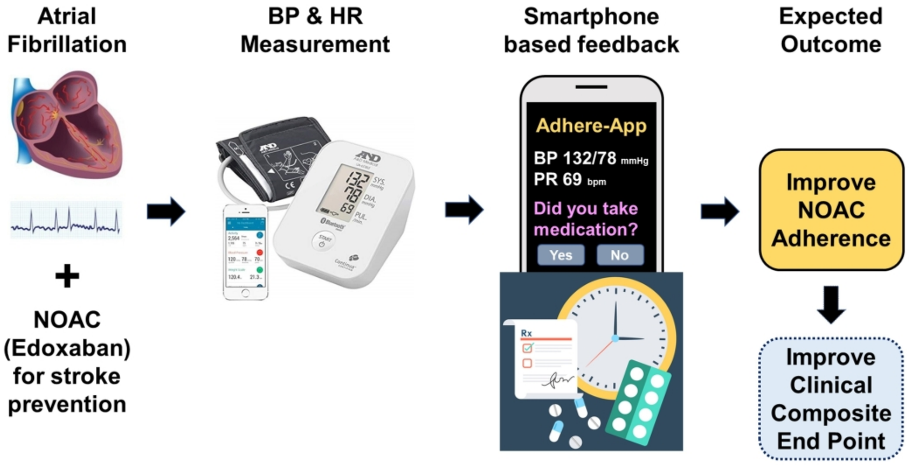

Fig. 1 Schematic diagram of expected improvement in NOAC adherence by regular measurement of BP, heart rate and smartphone feedback in patients with atrial fibrillation. NOAC, non-vitamin K oral anti-coagulant; BP, blood pressure; HR, heart rate.

307x163mm (300 x 300 DPI)

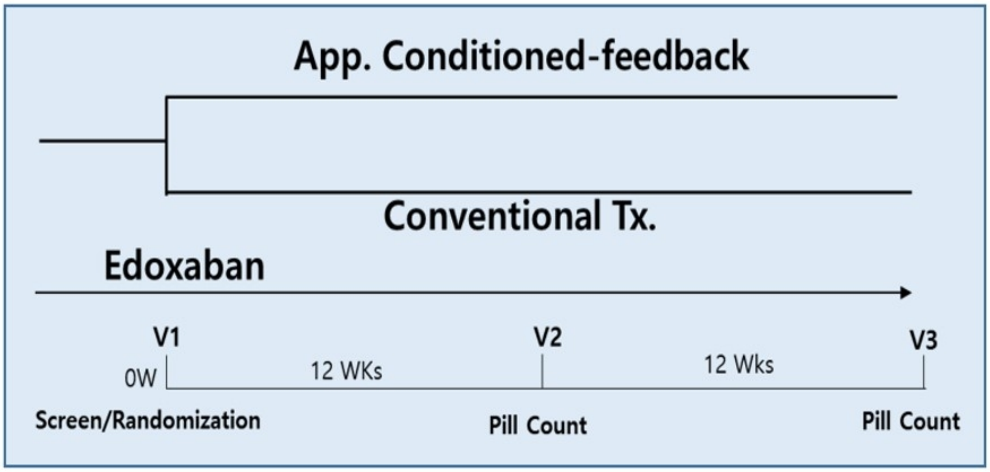

Fig. 2 Study design of AdhereApp study. Patients using edoxaban will be randomized to either an application conditioned-feedback group or a conventional therapy group.

233x114mm (96 x 96 DPI)

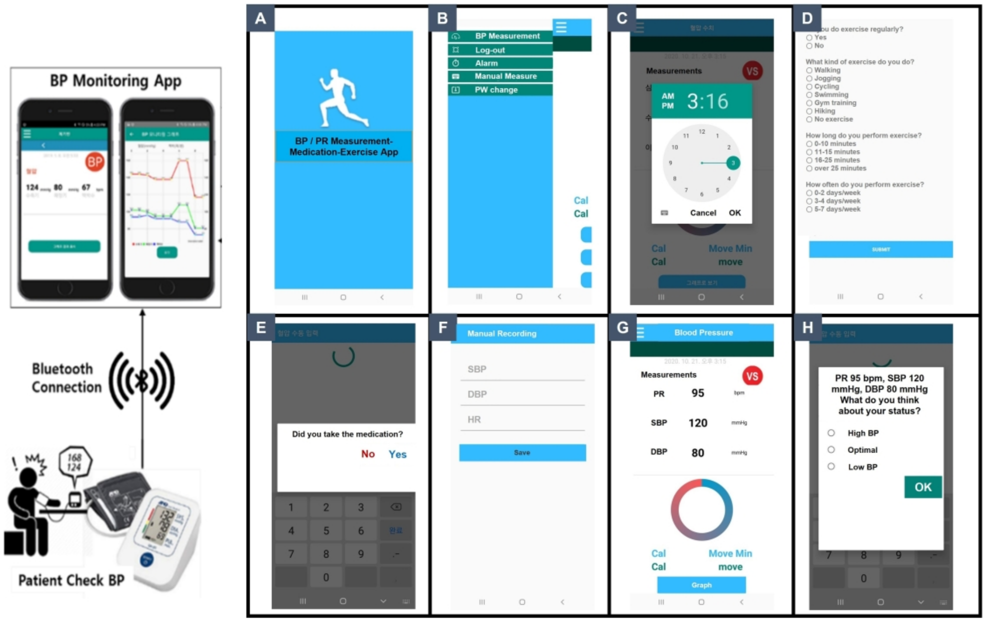

Fig. 3 Operating system and sample display of the smartphone app. When the patient measures BP, the data are automatically transferred to the app via Bluetooth system. A, title page; B, menu bar indicating BP measurement, log-out, alarm setting, manual BP input, and password setting; C, alarm setting page for medication; D, exercise information including interval, exercise type, and duration; E, alarm page to check whether the patient has taken medication; F, BP manual input page; G, page displaying measured BP and HR result; H, feed-back question to check whether the patient has achieved optimal target BP. BP, blood pressure; HR, heart rate.

310x190mm (300 x 300 DPI)

Supplementary Material

Rationale, Design, and Efficacy of a Smartphone Application for Improving Self-Awareness of Adherence to Edoxaban Treatment: A Randomized Controlled Study (Adhere App)

System Development Process

Previously, we described a five-step process to design and develop a standalone application (app) for patient blood pressure (BP) monitoring and physician's assistance. This app is currently undergoing clinical trial. In this study, we changed the last two steps of the previously described process. After a successful clinical trial (WHO ICRTP registry, ID: KCT0004754), we will evaluate the system. In this study, we developed an app with an additional functionality for monitoring the patient's physical activities. The primary functionalities of the system include patient BP and activity monitoring, medication adherence, and recommendations along with alert generation. The developed apps include a BP&Activity monitoring mobile app, electronic case report form (eCRF)-atrial fibrillation (AF), and Rule Editor. The primary users of the mobile app are registered patients, while the other two apps (eCRF-AF and Rule Editor) are mainly used by physicians to monitor the associated patients' activities and for knowledge creation, respectively. The aforementioned apps were designed and developed based on the five-step process. Supplemental Figure 1 shows the complete system design and development process.

For peer review only

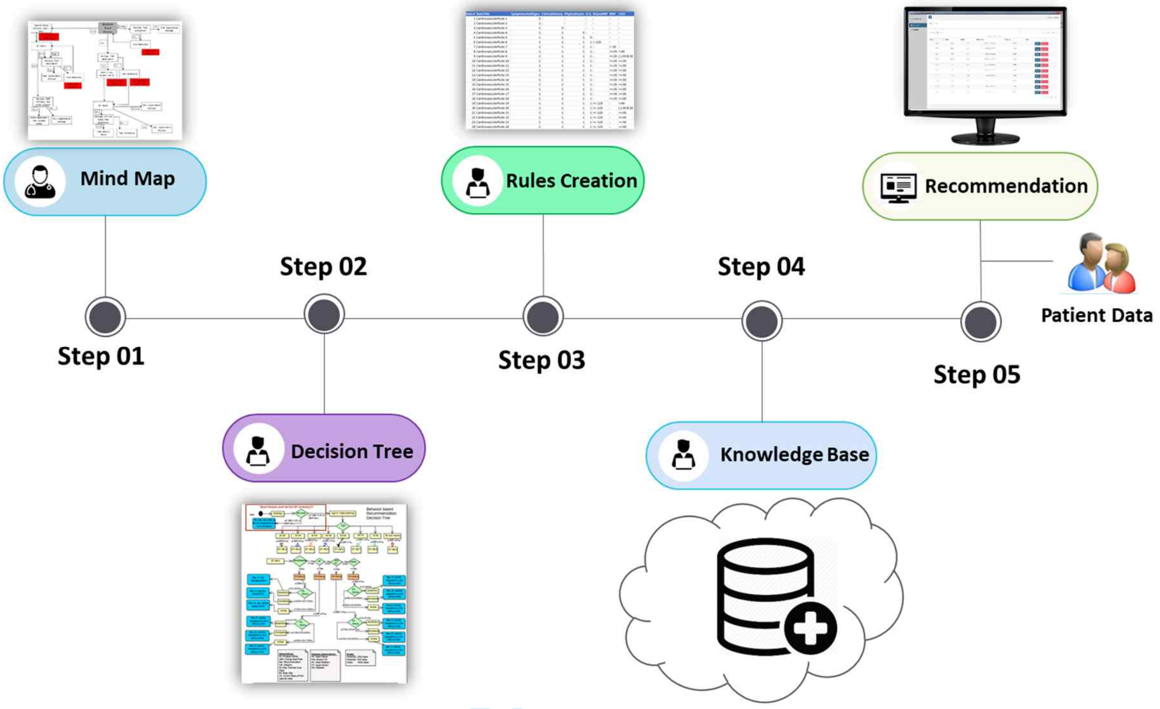

Supplemental Figure 1: System design and development process

- **Mind Map:** First, we represented the physicians' knowledge in an informal format, called the mind map, that is easily understandable to a human but not understandable to a computer. The physicians' knowledge about BP, physical activities, and their correlation was represented in the form of mind map nodes, and the nodes were related among each other based on the conditional relationships. With this analogy, the BP is linked with the age of the patient to identify the recommended activity for the patient. The resultant recommendations about the BP (systolic and diastolic), age, and current status of patient activity are connected to further diagnosis and then treatment of the patient's current condition. In short, physicians' knowledge based on their experience during real practice is represented in the mind map. We modify the initial mind map based on discussion with concerned physicians during a knowledge elicitation phase, and the final mind map is produced after a few knowledge elicitation meetings.
- **Iterative Decision Tree:** In the second phase, we transform the non-formal representation of the mind map into a more formal representation, known as the Iterative Decision Tree (IDT) [1]. The IDT representation is more understandable for physicians and computers; however,

transformation is needed for execution. In the IDT, the contributing factors are highlighted and linked with different tests to cover all the possible paths to reach diagnostic and treatment decisions. In this study, we recommend suitable physical activities to the patients based on their ages. Therefore, the patient first checks their BP, and then the system will generate a recommendation based on the recorded BP and the patient's age. Exercise goal steps (EGS) are calculated and recommended by the system to the patient to achieve the specified goal. Physicians' knowledge regarding BP is correlated with the physical activity, as shown in Supplemental Figure 2. The physicians verified and improved the IDT for all possible cases of that disease in multiple iterations of the knowledge elicitation process.

For peer review only

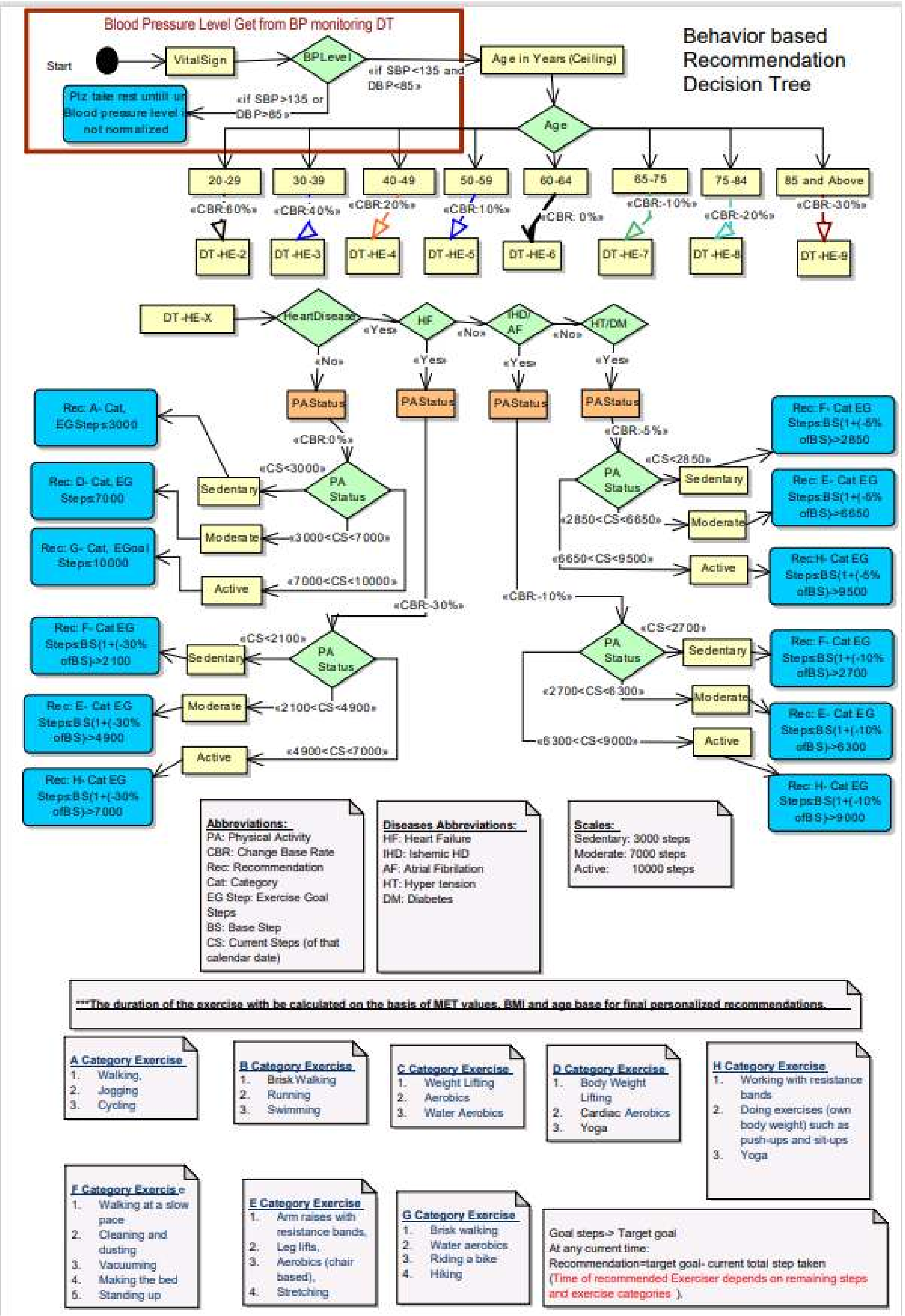

Supplemental Figure 2: Final Iterative Decision Tree (IDT) for blood pressure with physical activity

- **Rule Creation:** As previously mentioned, the IDT representation needs to be transformed into an executable format for the operative recommendations. Therefore, in the rule creation process, we transform the IDT into production rules. The production rules in object-oriented knowledge

bases are an easy way for knowledge execution. In this step, all the IDT branches are transformed into rules by top-down and left-right traversing and reasoning approaches. Our developed knowledge environment, called the Intelligent-Knowledge Authoring Tool (I-KAT), is used to transform rules into production and executable formats.

- **Knowledge Maintenance:** The knowledge maintenance phase provides a sophisticated method for validation and verification of the knowledge base. The transformed rules in the previous step are retrieved for validation and verification using a rule-based reasoning methodology. This aims to find conflict and duplication in the existing rules, if they exist. In this phase, all the rules are stored in the knowledge base in the proper hierarchy of knowledge representation.
- **Recommendation:** The recommendation phase is needed to give recommendations based on the knowledge created in the previous step. Therefore, an app is developed to provide an executable environment, which consists of a rule-based reasoner and a user-friendly interface to handle the input of signs, symptoms, history, and medical test reports to produce a final diagnosis and treatment. A mobile app was developed for BP and physical activity monitoring interventions to support patients. The app prompts the patient to monitor their BP and asks about medication intake. Based on their BP condition and medication intake status, the app recommends a suitable physical activity to perform on right time. If the condition is severe, the app recommends approaching the hospital urgently.

System Architecture and Structure

Supplemental Figure 3 shows the architecture and holistic structure of the system, which consists of four core components: (I) BP&Activity monitoring app, (II) knowledge acquisition and reasoning, (III) database, and (IV) eCRF-AF.

For peer review only

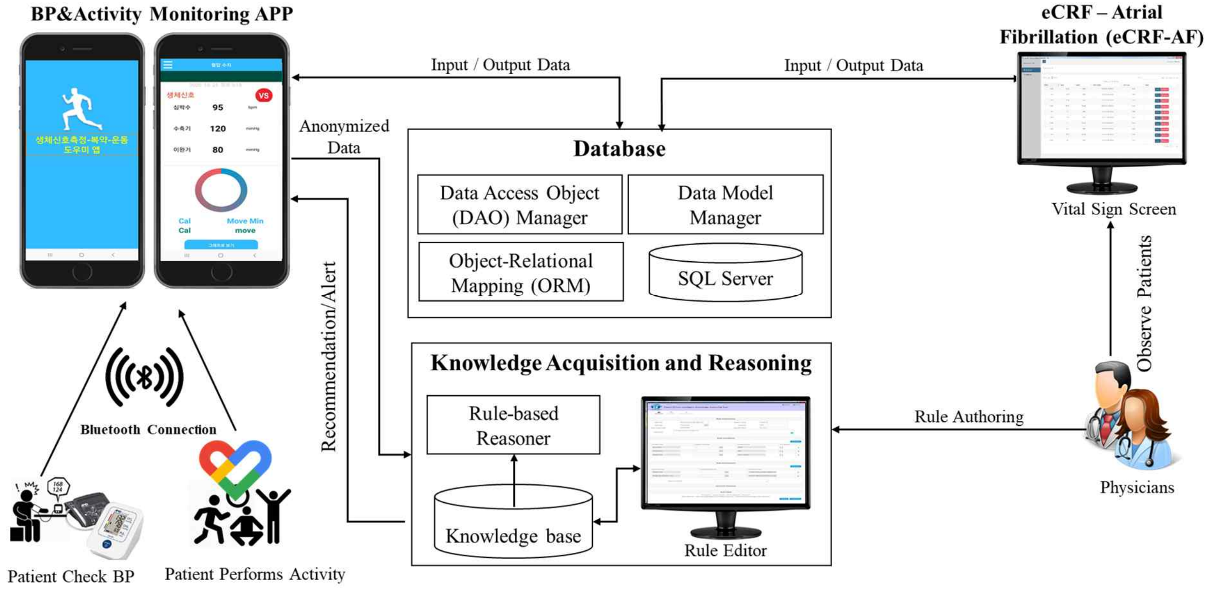

Supplemental Figure 3: System architecture and structural workflow

I. **BP&Activity Monitoring App:** This mobile app is equipped with different functionalities, such as BP and physical activity monitoring, user authentication, user registration, user exercise preferences, dashboard, BP monitoring graph, step-count graph, and recommendation screens. The core screen of the BP&Activity monitoring app is the dashboard, which facilitates users to connect with the BP monitoring device, such as the Bluetooth-enabled BP monitor UA-651BLE (A&D medical, Sydney, Australia). The dashboard screen is responsible for connecting the app with a nearby A&D PB monitoring device via Bluetooth. Similarly, the dashboard has implicit services to count the patients' steps while walking, running, jogging, and performing other physical activities. When the users check their BP, the calculated systolic BP (SBP), diastolic BP (DBP), and heart rate (HR) are sent to the mobile app. The app activates the reasoner to provide recommendations based on the calculated values. In this study, the app also considers patient age and current behavior regarding physical activity.

II. **Knowledge acquisition and reasoning:** In this component, our system provides a knowledge creation environment and reasoning capability. The I-KAT is equipped with a Rule Editor, which facilitates the creation, modification, and management of the knowledge base by physicians. The knowledge base is a core component of the clinical decision support system to

For peer review only - http://bmjopen.bmj.com/site/about/guidelines.xhtml

store and maintain clinical knowledge. We are presenting an authoring environment called a Rule Editor that provides an easy-to-use interface for physicians to create clinical knowledge rules. The tool reduces the burden of memorizing and understanding the data model and vocabulary concepts for the physician because the system is equipped with intelli-sense functionality during rule creation. The reasoning capability also handles knowledge validation and verification to find duplicated and conflicted rules within the knowledge base. Finally, the rules persist in the knowledge base. When it receives a patient's recorded BP, the rule-based reasoner is activated to generate recommendations and alerts based on the recorded BP.

III. **Database:** We designed and implemented the database schema to maintain patient demographic data, vital signs (systolic BP, diastolic BP, and HR), physical activity data, and other patient-related data in the database. We implemented two types of database storage: the one for the mobile app is a lightweight database using SQL Lite, which is the default temporary storage for patient data on the smartphone, when the device is not connected to the internet. When the device is connected to the internet, the data are sent to an SQL server for long-term (permanent) storage. Three internal components, data model manager, data access object (DAO) manager, and object relational mapping (ORM) work collaboratively to perform the persistence functionality. The data model manager is responsible for managing the database schema along with data instances to store in the appropriate model. The data access object (DAO) manager is used to access the data objects of the recorded values and prepare the data model to store in the database. The object relational mapping (ORM) is used to convert data between incompatible type systems using object-oriented programming languages. The App Server/Rest API is designed and developed for storing patient information, with patient consensus, for tele-monitoring by the concerned physicians. The patient data are anonymized during communication and persistence. Therefore, only the authorized and concerned physicians can see and analyze only their assigned patients' data.

IV. **eCRF–atrial fibrillation**: Generally, the eCRF system collects patient data during clinical trials. The eCRF system usually collects patient vital sign information. SBP, DBP, and HR are

For peer review only

important vital signs that are managed within the eCRF system. We designed and developed a special purpose eCRF for patients with AF, called eCRF-AF to monitor vital signs (SBP, DBP, and HR) along with physical activities of patients. Therefore, the BP&Activity monitoring app sends the SBP, DBP, and HR and the step counts along with physical activities (sitting, walking, running, jogging, etc.) to the vital sign and physical activity repositories, respectively. These are shown to the physicians on separate analytical screens depending on the nature of the data. The eCRF-AF systems and HMIS systems of different hospitals can be integrated with the BP&Activity monitoring app. We focused and integrated the BP&Activity monitoring app with a lightweight eCRF-AF system developed for managing clinical trials. Physicians can easily observe the BP trends (daily, weekly, monthly, yearly, etc.) along with exercise, using physical activity trends of the concerned patients. If the physicians find an abnormality in the patient data, they will communicate with the respective patient.

System Implementation Outcome: Process Flow

The system comprises three main apps: one is a mobile app (BP&Activity monitoring app), which is a patient-oriented app, while the other two are web apps (eCRF-AF and Rule Editor), which are physician-oriented apps. Here, we focus on the patient-oriented BP&Activity monitoring app, which comprises 11 different screens with multiple functionalities. The BP&Activity monitoring app and its utilization and process flow are depicted in **Supplemental Figure 4**. The BP&Activity monitoring app has two processes: one process for new user registration and a second process for registered patients to check their BPs and physical activities to obtain suitable and correct recommendations based on the recorded BP, age, profile questionnaire, and exercise preferences.

User Registration Process: To register a new user on the system, we follow a three-step process to obtain user information.

- **Step 1:** The user will add profile information, such as name, gender, date of birth, and email address, and other information to register on the system.
- **Step 2:** The questionnaire screen is used to investigate physical behavior in terms of type of

For peer review only

exercise, duration of exercise, and frequency of exercise. All these questions help to calculate the patients' current behavior in terms of physical activity.

- **Step 3:** The preferences screen gives privileges to the user to select the appropriate exercises from the recommended ones to set the final recommendation as a preference.

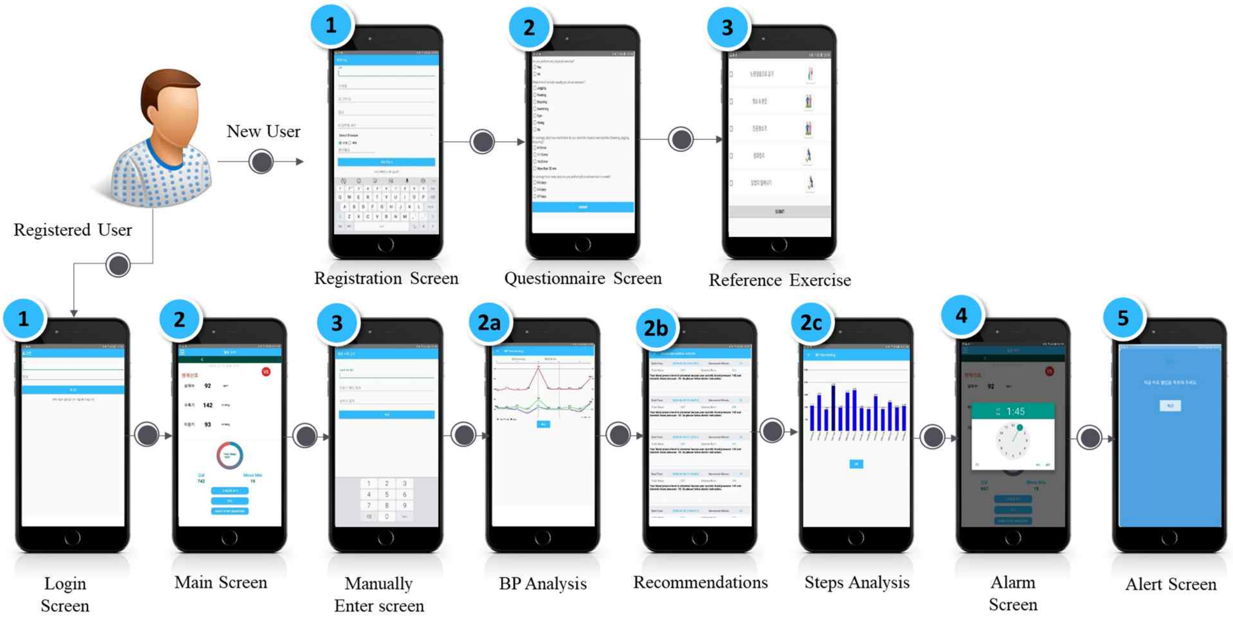

Supplemental Figure 4: Utilization and process flow of BP&Activity monitoring app (Patient-Oriented)

BP and Activity Monitoring Process: After successful registration, the patient can utilize the app to monitor their vital signs (SBP, DBP, and HR) along with their physical activity status through an A&D BP monitoring device and GoogleFit API.

- **Step 1:** The user needs to login to the system with his/her credentials for authentication.
- **Step 2:** After successful authentication, the user can access a dashboard screen. It consists of the current status of their vital signs along with the step count. It also facilitates observation of analytics and recommendation history.
- **Step 3:** The user can enter vital sign details manually when the attached device is not communicating properly. The app accepts SBP, DBP, and HR values.
- **Step 2a:** From the dashboard, the user can visualize historical analytics for vital signs, which

represent the health status in terms of BP and HR.

- **Step 2b:** From the dashboard, the user can visualize historical recommendations regarding exercises based on their physical behavior with respect to time.
- **Step 2c:** From the dashboard, the user can visualize step count historical analytics, with respect to the day, to understand their own physical behavior.
- **Step 4:** The alarm screen facilitates the user to decide and set an alarm for checking their BP. At the selected time, the system will generate the alarm for the user to check their BP and take their medication, as necessary.
- **Step 5:** The system generates recommendations at different stages of BP and activity monitoring based on patient preferences, age, and answers to the questionnaire. The recommendation may issue a message to check BP, walk, run, jog, and rest for a specified time, or to take medication at the specified time.

For peer review only

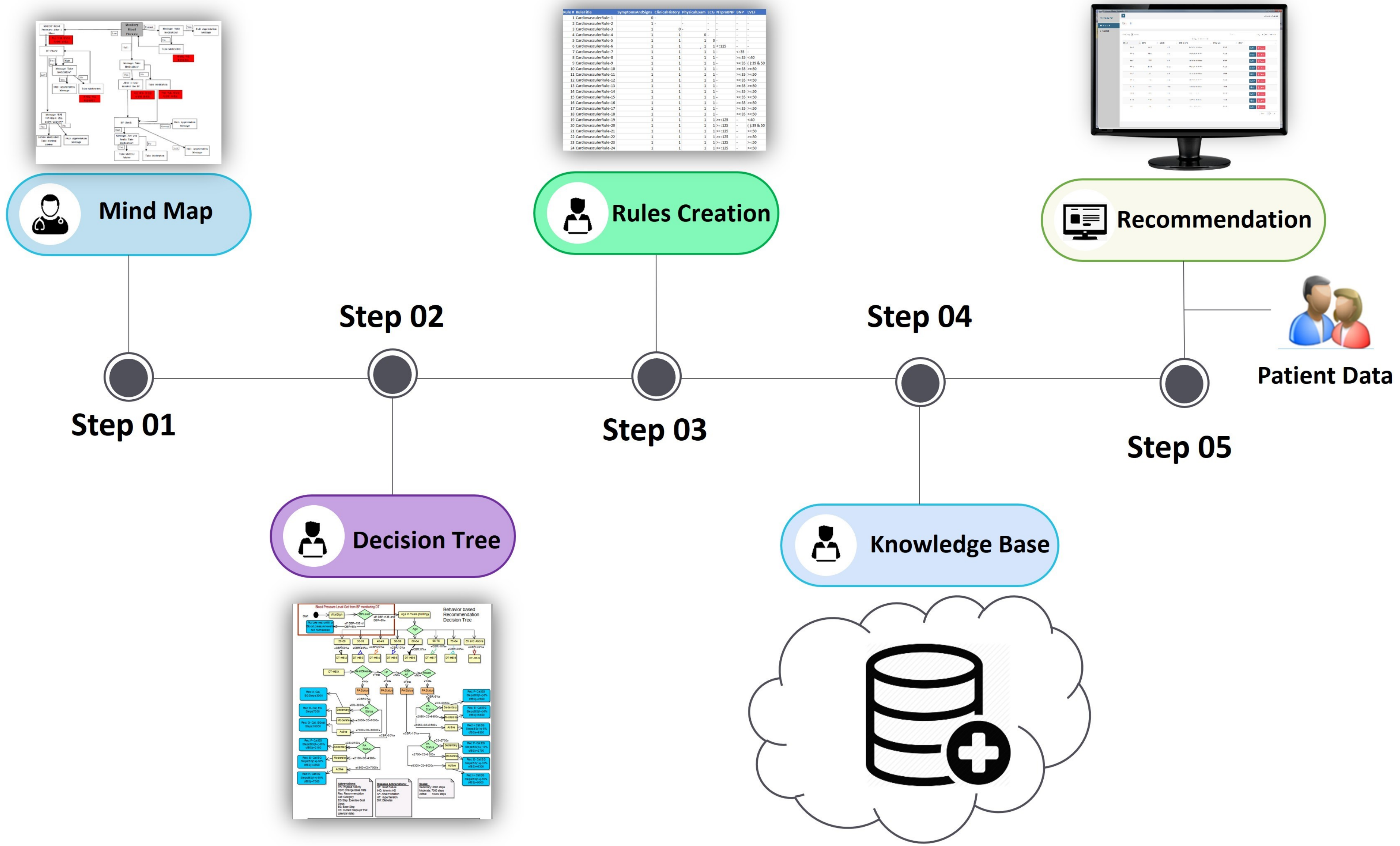

Mind Map
Step 01
Step 02
Decision Tree
Rules Creation
Step 03
Step 04
Knowledge Base
Recommendation
Patient Data
Step 05

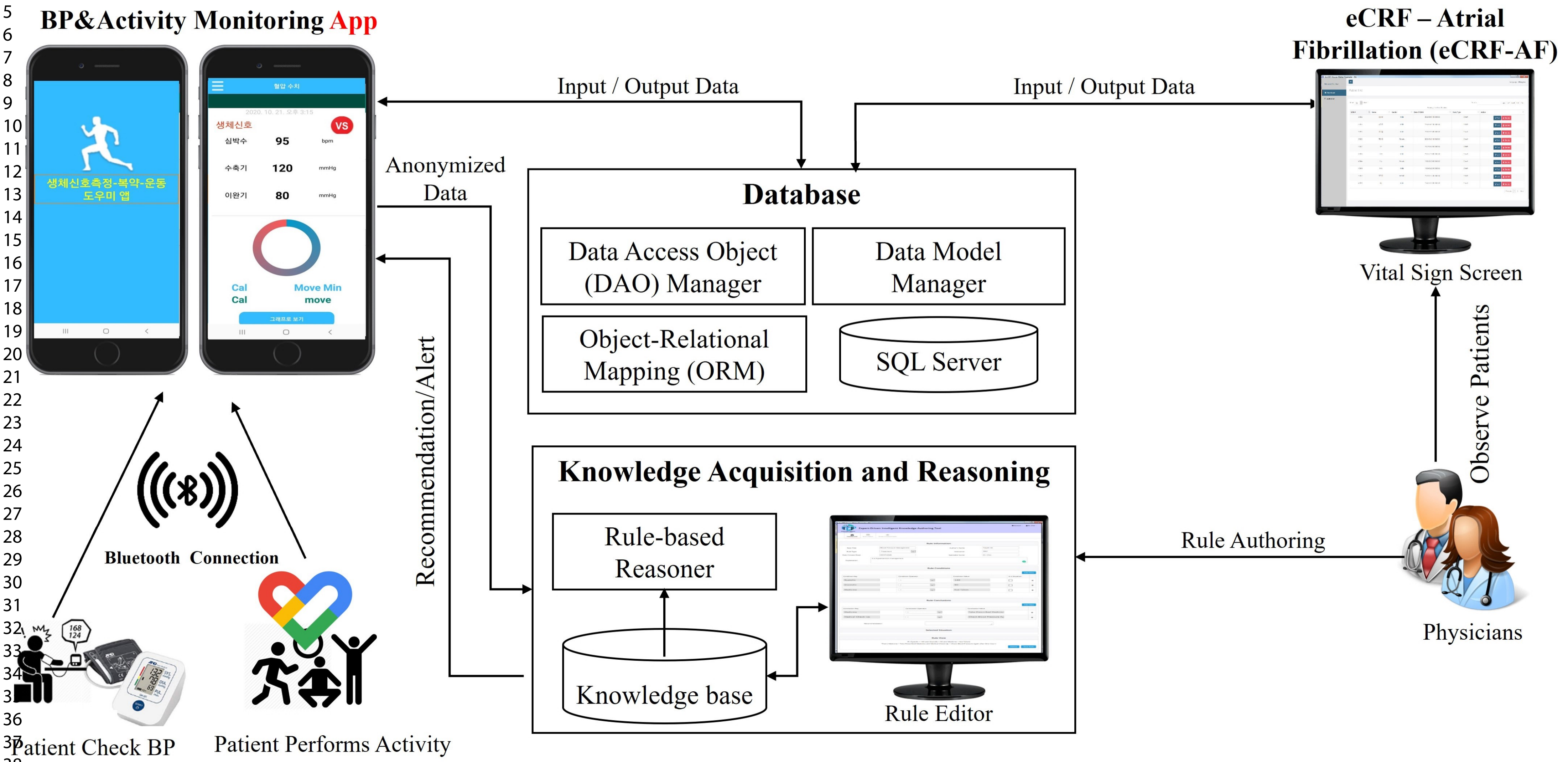
BP&Activity Monitoring App
생체신호측정-복약-운동
도우미 앱
생체신호
심박수 95 bpm
수축기 120 mmHg
이완기 80 mmHg
VS
Cal
Cal
Move Min
move
eCRF – Atrial Fibrillation (eCRF-AF)
Input / Output Data
Input / Output Data
Anonymized Data
Database
Data Access Object (DAO) Manager
Data Model Manager
Object-Relational Mapping (ORM)
SQL Server
Vital Sign Screen
Recommendation/Alert
Observe Patients
Knowledge Acquisition and Reasoning
Rule-based Reasoner
Knowledge base
Rule Editor
Rule Authoring
Physicians
Bluetooth Connection
Patient Check BP
Patient Performs Activity

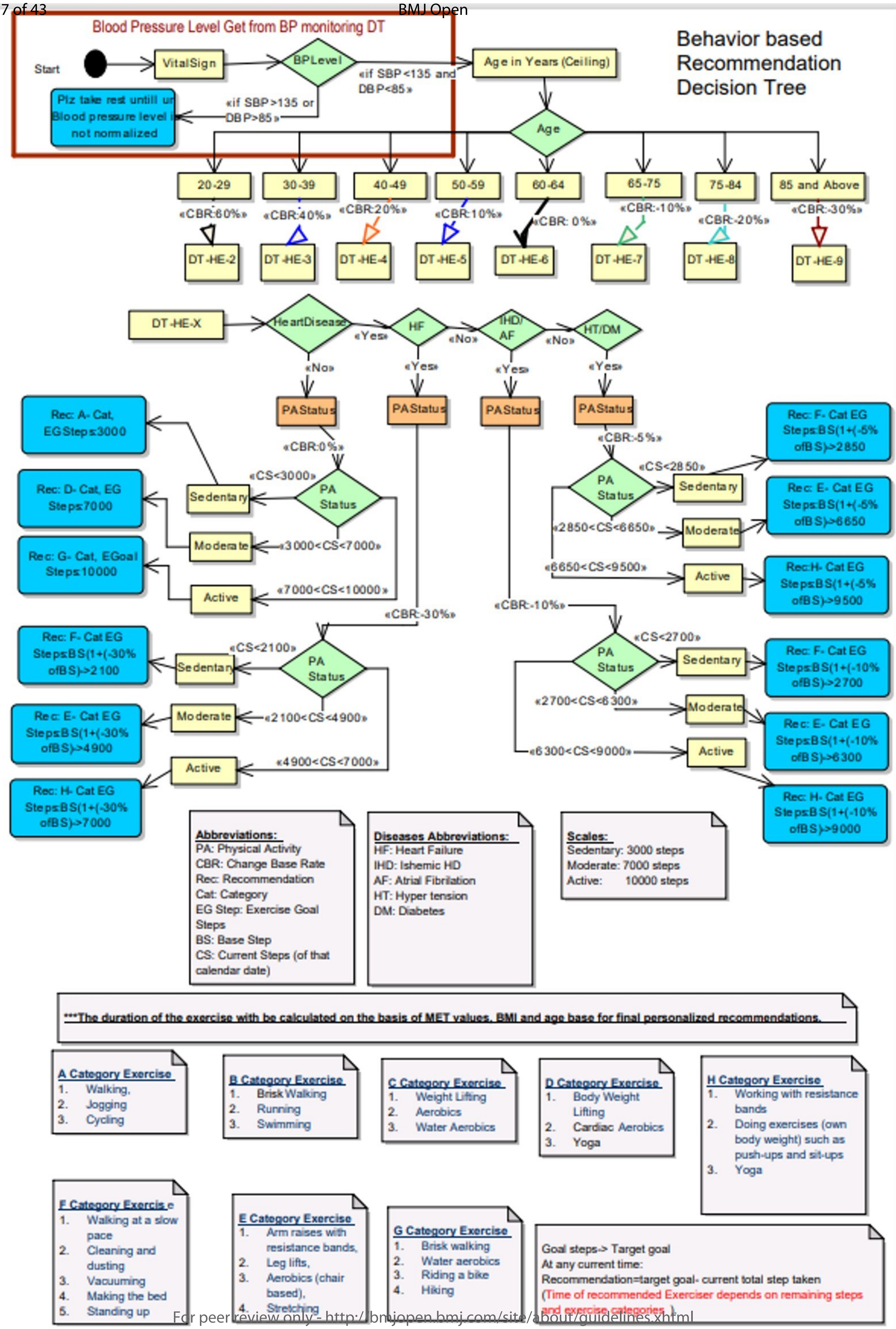
Blood Pressure Level Get from BP monitoring DT
Start
VitalSign
BPLevel
«if SBP<135 and DBP<85»
«if SBP>135 or DBP>85»
Plz take rest untill ur Blood pressure level is not normalized
Age in Years (Ceiling)
Behavior based Recommendation Decision Tree
Age
20-29
30-39
40-49
50-59
60-64
65-75
75-84
85 and Above
«CBR:60%»
«CBR:40%»
«CBR:20%»
«CBR:10%»
«CBR: 0%»
«CBR:-10%»
«CBR:-20%»
«CBR:-30%»
DT-HE-2
DT-HE-3
DT-HE-4
DT-HE-5
DT-HE-6
DT-HE-7
DT-HE-8
DT-HE-9
DT-HE-X
HeartDisease
«Yes»
HF
«No»
IHD/AF
«No»
HT/DM
«No»
«Yes»
«Yes»
«Yes»
PAStatus
PAStatus
PAStatus
PAStatus
«CBR:0%»
PA Status
«CS<3000»
Sedentary
Rec: A- Cat, EGSteps:3000
Moderate
«3000<CS<7000»
Rec: D- Cat, EG Steps:7000
Active
«7000<CS<10000»
Rec: G- Cat, EGoal Steps:10000
«CBR:-30%»
PA Status
«CS<2100»
Sedentary
Rec: F- Cat EG Steps:BS(1+(-30% ofBS)->2100
Moderate
«2100<CS<4900»
Rec: E- Cat EG Steps:BS(1+(-30% ofBS)->4900
Active
«4900<CS<7000»
Rec: H- Cat EG Steps:BS(1+(-30% ofBS)->7000
«CBR:-5%»
PA Status
«CS<2850»
Sedentary
Rec: F- Cat EG Steps:BS(1+(-5% ofBS)->2850
«2850<CS<6650»
Moderate
Rec: E- Cat EG Steps:BS(1+(-5% ofBS)->6650
«6650<CS<9500»
Active
Rec:H- Cat EG Steps:BS(1+(-5% ofBS)->9500
«CBR:-10%»
PA Status
«CS<2700»
Sedentary
Rec: F- Cat EG Steps:BS(1+(-10% ofBS)->2700
«2700<CS<6300»
Moderate
Rec: E- Cat EG Steps:BS(1+(-10% ofBS)->6300
«6300<CS<9000»
Active
Rec: H- Cat EG Steps:BS(1+(-10% ofBS)->9000
Abbreviations:
PA: Physical Activity
CBR: Change Base Rate
Rec: Recommendation
Cat: Category
EG Step: Exercise Goal Steps
BS: Base Step
CS: Current Steps (of that calendar date)
Diseases Abbreviations:
HF: Heart Failure
IHD: Ishemic HD
AF: Atrial Fibrillation
HT: Hyper tension
DM: Diabetes
Scales:
Sedentary: 3000 steps
Moderate: 7000 steps
Active: 10000 steps
***The duration of the exercise with be calculated on the basis of MET values, BMI and age base for final personalized recommendations.
A Category Exercise
1. Walking,
2. Jogging
3. Cycling
B Category Exercise
1. Brisk Walking
2. Running
3. Swimming
C Category Exercise
1. Weight Lifting
2. Aerobics
3. Water Aerobics
D Category Exercise
1. Body Weight Lifting
2. Cardiac Aerobics
3. Yoga
H Category Exercise
1. Working with resistance bands
2. Doing exercises (own body weight) such as push-ups and sit-ups
3. Yoga
F Category Exercise
1. Walking at a slow pace
2. Cleaning and dusting
3. Vacuuming
4. Making the bed
5. Standing up
E Category Exercise
1. Arm raises with resistance bands,
2. Leg lifts,
3. Aerobics (chair based),
4. Stretching
G Category Exercise
1. Brisk walking
2. Water aerobics
3. Riding a bike
4. Hiking
Goal steps-> Target goal
At any current time:
Recommendation=target goal- current total step taken
(Time of recommended Exerciser depends on remaining steps and exercise categories.)

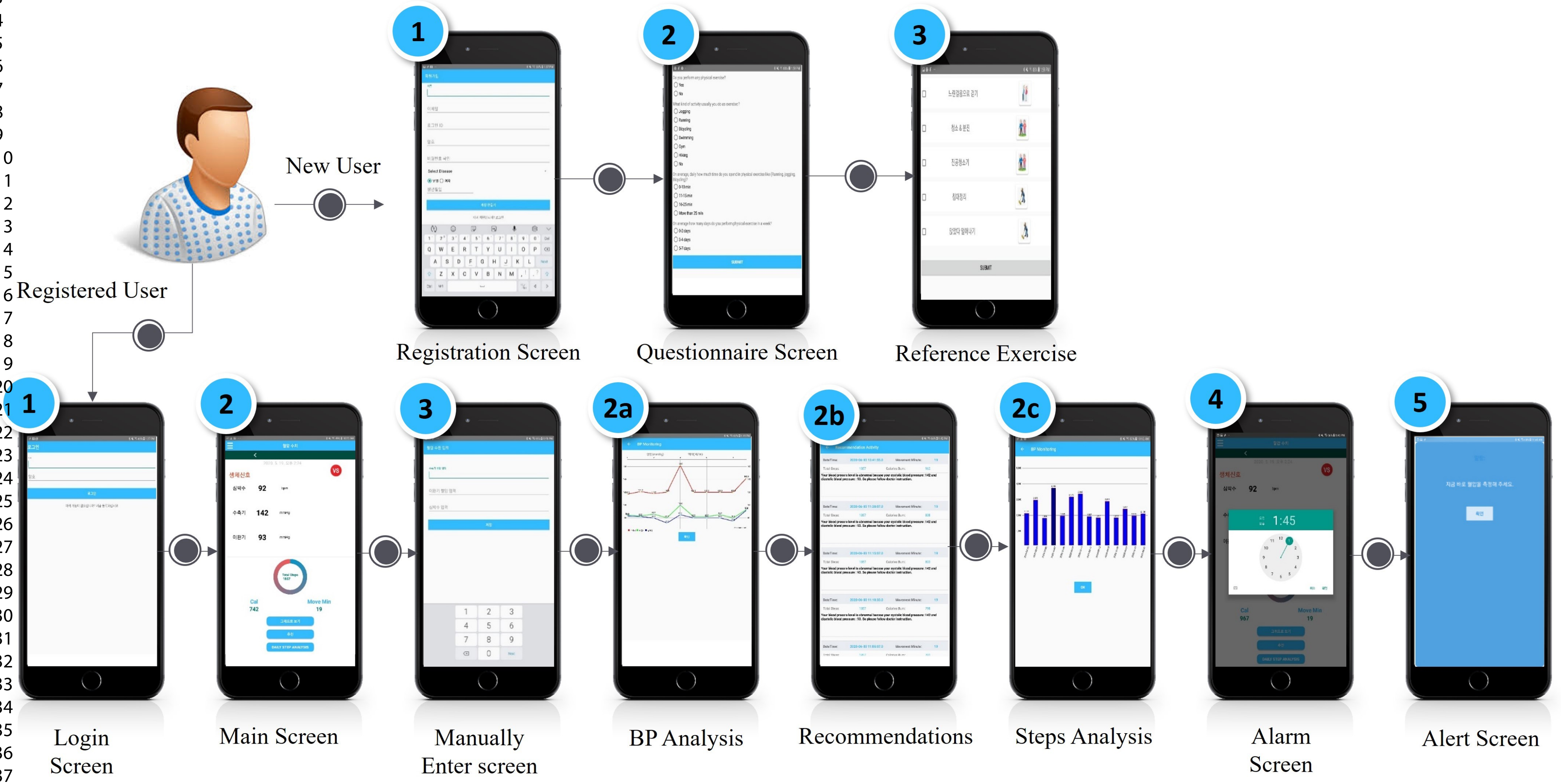
New User
Registered User
1
2
3
Registration Screen
Questionnaire Screen
Reference Exercise
1
2
3
2a
2b
2c
4
5
Login Screen
Main Screen
Manually Enter screen
BP Analysis
Recommendations
Steps Analysis
Alarm Screen
Alert Screen

Reporting checklist for protocol of a clinical trial.

Based on the SPIRIT guidelines.

Instructions to authors

Complete this checklist by entering the page numbers from your manuscript where readers will find each of the items listed below.

Your article may not currently address all the items on the checklist. Please modify your text to include the missing information. If you are certain that an item does not apply, please write "n/a" and provide a short explanation.

Upload your completed checklist as an extra file when you submit to a journal.

In your methods section, say that you used the SPIRITreporting guidelines, and cite them as:

Chan A-W, Tetzlaff JM, Gøtzsche PC, Altman DG, Mann H, Berlin J, Dickersin K, Hróbjartsson A, Schulz KF, Parulekar WR, Krleža-Jerić K, Laupacis A, Moher D. SPIRIT 2013 Explanation and Elaboration: Guidance for protocols of clinical trials. BMJ. 2013;346:e7586

		Reporting Item	Page Number
**Administrative information**			
Title	#1	Descriptive title identifying the study design, population, interventions, and, if applicable, trial acronym	1
Trial registration	#2a	Trial identifier and registry name. If not yet registered, name of intended registry	1
Trial registration: data set	#2b	All items from the World Health Organization Trial Registration Data Set	1
Protocol version	#3	Date and version identifier	8
Funding	#4	Sources and types of financial, material, and other support	16
Roles and responsibilities: contributorship	#5a	Names, affiliations, and roles of protocol contributors	15

For peer review only

Roles and responsibilities: sponsor contact information	#5b	Name and contact information for the trial sponsor	16
Roles and responsibilities: sponsor and funder	#5c	Role of study sponsor and funders, if any, in study design; collection, management, analysis, and interpretation of data; writing of the report; and the decision to submit the report for publication, including whether they will have ultimate authority over any of these activities	16
Roles and responsibilities: committees	#5d	Composition, roles, and responsibilities of the coordinating centre, steering committee, endpoint adjudication committee, data management team, and other individuals or groups overseeing the trial, if applicable (see Item 21a for data monitoring committee)	11
**Introduction**			
Background and rationale	#6a	Description of research question and justification for undertaking the trial, including summary of relevant studies (published and unpublished) examining benefits and harms for each intervention	6
Background and rationale: choice of comparators	#6b	Explanation for choice of comparators	6
Objectives	#7	Specific objectives or hypotheses	6
Trial design	#8	Description of trial design including type of trial (eg, parallel group, crossover, factorial, single group), allocation ratio, and framework (eg, superiority, equivalence, non-inferiority, exploratory)	7
**Methods: Participants, interventions, and outcomes**			
Study setting	#9	Description of study settings (eg, community clinic, academic hospital) and list of countries where data will be collected. Reference to where list of study sites can be obtained	7
Eligibility criteria	#10	Inclusion and exclusion criteria for participants. If applicable, eligibility criteria for study centres and individuals who will	8

For peer review only

		perform the interventions (eg, surgeons, psychotherapists)	
Interventions: description	#11a	Interventions for each group with sufficient detail to allow replication, including how and when they will be administered	10
Interventions: modifications	#11b	Criteria for discontinuing or modifying allocated interventions for a given trial participant (eg, drug dose change in response to harms, participant request, or improving / worsening disease)	11
Interventions: adherance	#11c	Strategies to improve adherence to intervention protocols, and any procedures for monitoring adherence (eg, drug tablet return; laboratory tests)	10
Interventions: concomitant care	#11d	Relevant concomitant care and interventions that are permitted or prohibited during the trial	10
Outcomes	#12	Primary, secondary, and other outcomes, including the specific measurement variable (eg, systolic blood pressure), analysis metric (eg, change from baseline, final value, time to event), method of aggregation (eg, median, proportion), and time point for each outcome. Explanation of the clinical relevance of chosen efficacy and harm outcomes is strongly recommended	11
Participant timeline	#13	Time schedule of enrolment, interventions (including any run-ins and washouts), assessments, and visits for participants. A schematic diagram is highly recommended (see Figure)	22
Sample size	#14	Estimated number of participants needed to achieve study objectives and how it was determined, including clinical and statistical assumptions supporting any sample size calculations	12
Recruitment	#15	Strategies for achieving adequate participant enrolment to reach target sample size	10
**Methods: Assignment of interventions (for controlled trials)**			
Allocation: sequence generation	#16a	Method of generating the allocation sequence (eg, computer-generated random numbers), and list of any factors for stratification. To reduce predictability of a random sequence, details of any planned restriction (eg, blocking) should be provided in a separate document that is unavailable to those who enrol participants or assign interventions	10

For peer review only

Allocation concealment mechanism	#16b	Mechanism of implementing the allocation sequence (eg, central telephone; sequentially numbered, opaque, sealed envelopes), describing any steps to conceal the sequence until interventions are assigned	10
Allocation: implementation	#16c	Who will generate the allocation sequence, who will enrol participants, and who will assign participants to interventions	10
Blinding (masking)	#17a	Who will be blinded after assignment to interventions (eg, trial participants, care providers, outcome assessors, data analysts), and how	10
Blinding (masking): emergency unblinding	#17b	If blinded, circumstances under which unblinding is permissible, and procedure for revealing a participant's allocated intervention during the trial	10
**Methods: Data collection, management, and analysis**			
Data collection plan	#18a	Plans for assessment and collection of outcome, baseline, and other trial data, including any related processes to promote data quality (eg, duplicate measurements, training of assessors) and a description of study instruments (eg, questionnaires, laboratory tests) along with their reliability and validity, if known. Reference to where data collection forms can be found, if not in the protocol	9
Data collection plan: retention	#18b	Plans to promote participant retention and complete follow-up, including list of any outcome data to be collected for participants who discontinue or deviate from intervention protocols	11
Data management	#19	Plans for data entry, coding, security, and storage, including any related processes to promote data quality (eg, double data entry; range checks for data values). Reference to where details of data management procedures can be found, if not in the protocol	11
Statistics: outcomes	#20a	Statistical methods for analysing primary and secondary outcomes. Reference to where other details of the statistical analysis plan can be found, if not in the protocol	11
Statistics: additional analyses	#20b	Methods for any additional analyses (eg, subgroup and adjusted analyses)	12

For peer review only

Statistics: analysis population and missing data	#20c	Definition of analysis population relating to protocol non-adherence (eg, as randomised analysis), and any statistical methods to handle missing data (eg, multiple imputation)	12
**Methods: Monitoring**			
Data monitoring: formal committee	#21a	Composition of data monitoring committee (DMC); summary of its role and reporting structure; statement of whether it is independent from the sponsor and competing interests; and reference to where further details about its charter can be found, if not in the protocol. Alternatively, an explanation of why a DMC is not needed	15
Data monitoring: interim analysis	#21b	Description of any interim analyses and stopping guidelines, including who will have access to these interim results and make the final decision to terminate the trial	15
Harms	#22	Plans for collecting, assessing, reporting, and managing solicited and spontaneously reported adverse events and other unintended effects of trial interventions or trial conduct	13
Auditing	#23	Frequency and procedures for auditing trial conduct, if any, and whether the process will be independent from investigators and the sponsor	13
**Ethics and dissemination**			
Research ethics approval	#24	Plans for seeking research ethics committee / institutional review board (REC / IRB) approval	13
Protocol amendments	#25	Plans for communicating important protocol modifications (eg, changes to eligibility criteria, outcomes, analyses) to relevant parties (eg, investigators, REC / IRBs, trial participants, trial registries, journals, regulators)	13
Consent or assent	#26a	Who will obtain informed consent or assent from potential trial participants or authorised surrogates, and how (see Item 32)	13
Consent or assent: ancillary studies	#26b	Additional consent provisions for collection and use of participant data and biological specimens in ancillary studies, if applicable	13
Confidentiality	#27	How personal information about potential and enrolled participants will be collected, shared, and maintained in order to protect confidentiality before, during, and after the trial	9

For peer review only

Declaration of interests	#28	Financial and other competing interests for principal investigators for the overall trial and each study site	16
Data access	#29	Statement of who will have access to the final trial dataset, and disclosure of contractual agreements that limit such access for investigators	13
Ancillary and post trial care	#30	Provisions, if any, for ancillary and post-trial care, and for compensation to those who suffer harm from trial participation	13
Dissemination policy: trial results	#31a	Plans for investigators and sponsor to communicate trial results to participants, healthcare professionals, the public, and other relevant groups (eg, via publication, reporting in results databases, or other data sharing arrangements), including any publication restrictions	13
Dissemination policy: authorship	#31b	Authorship eligibility guidelines and any intended use of professional writers	13
Dissemination policy: reproducible research	#31c	Plans, if any, for granting public access to the full protocol, participant-level dataset, and statistical code	13
**Appendices**			
Informed consent materials	#32	Model consent form and other related documentation given to participants and authorised surrogates	10
Biological specimens	#33	Plans for collection, laboratory evaluation, and storage of biological specimens for genetic or molecular analysis in the current trial and for future use in ancillary studies, if applicable	11

The SPIRIT Explanation and Elaboration paper is distributed under the terms of the Creative Commons Attribution License CC-BY-NC. This checklist was completed on 21. July 2021 using https://www.goodreports.org/, a tool made by the EQUATOR Network in collaboration with Penelope.ai

For peer review only
